# Panorama des pathologies infectieuses et non infectieuses de Polynésie française en 2025

**DOI:** 10.48327/mtsi.v5i3.2025.714

**Published:** 2025-07-03

**Authors:** Erwan OEHLER, Rémi MAYAN, Stéphane LASTÈRE, Jean-Marc SÉGALIN, Bertrand REMAUDIÈRE, Lam NGUYEN, Jérôme DEBACRE, Bertrand CONDAT, Van-Mai CAO-LORMEAU, Hervé BOSSIN, Clémence GATTI HOWELL, Marine JULLIEN, Sébastien NUNEZ, Ronan DELAVAL, Raphaël BUON, Rainui RICHAUD, Éric PARRAT, Stéphane SAUGET, Pierre GUSTIN, Shari-Lane BOTCHE, Philippe GENET, Johan SEBTI, Moerani REREAO, Loïc DURAND, Philippe DUPIRE, Jean-François BUTAUD, Cristel THOMAS, Loïc EPELBOIN

**Affiliations:** 1 Service de médecine interne et polyvalente, Centre hospitalier de Polynésie française, Tahiti, Polynésie française; 2 Département de Santé publique, Direction de la Santé, Polynésie française; 3 Département de biologie médicale, Centre hospitalier de Polynésie française, Tahiti, Polynésie française; 4 Direction de la Santé, Centre du RAA, Pirae, Tahiti, Polynésie française; 5 Service d’aide médicale urgente (SAMU), Centre hospitalier de Polynésie française, Tahiti, Polynésie française; 6 Consultation de maladies infectieuses et tropicales (CMIT), Direction de la Santé, Polynésie française; 7 Direction de la Santé, Centre de lutte contre la tuberculose (CLCT), Polynésie française; 8 Service de gastro-entérologie, Centre hospitalier de Polynésie française, Tahiti, Polynésie française; 9 Laboratoire de recherche sur les infections virales émergentes, Institut Louis Malardé, Tahiti, Polynésie française; 10 Laboratoire d’entomologie médicale, UMR 241 SECOPOL, Institut Louis Malardé, Tahiti, Polynésie française; 11 Institut Louis Malardé, UMR 241 SECOPOL, Tahiti, Polynésie française; 12 Service de diabétologie-endocrinologie, Centre hospitalier de Polynésie française, Tahiti, Polynésie française; 13 Service de néphrologie, Centre hospitalier de Polynésie française, Tahiti, Polynésie française; 14 Service de neurologie, Centre hospitalier de Polynésie française, Tahiti, Polynésie française; 15 Service de cardiologie, Centre hospitalier de Polynésie française, Tahiti, Polynésie française; 16 Service de pneumologie, Centre hospitalier de Polynésie française, Tahiti, Polynésie française; 17 Service d’obstétrique, Centre hospitalier de Polynésie française, Tahiti, Polynésie française; 18 Service d’oncologie-radiothérapie, Centre hospitalier de Polynésie française, Tahiti, Polynésie française; 19 Institut polynésien du cancer, responsable registre et recherche, Tahiti, Polynésie française; 20 Service d’hématologie, Centre hospitalier de Polynésie française, Tahiti, Polynésie française; 21 Département de psychiatrie, Centre hospitalier de Polynésie française, Tahiti, Polynésie française; 22 MOODS Team, Inserm UMR 1018, CESP, Le Kremlin-Bicêtre; 23 Caisson hyperbare, Centre hospitalier de Polynésie française, Tahiti, Polynésie française; 24 Pharmacie, Centre hospitalier de Polynésie française, Tahiti, Polynésie française; 25 Consultant en foresterie et botanique polynésienne, Correspondant du Muséum national d’histoire naturelle (PatriNat), Tahiti, Polynésie française; 26 Unité des maladies infectieuses et tropicales et CIC Inserm 1424, Centre hospitalier de Cayenne, Cayenne, Guyane

**Keywords:** Médecine des voyages, Médecine tropicale, Épidémiologie, Polynésie française, Océan Pacifique, Travel medicine, Tropical medicine, Epidemiology, French Polynesia, Pacific Ocean

## Abstract

Tahiti ou le « mythe du Paradis », Bora Bora ou la « Perle du Pacifique ». Qui n’a jamais voulu prendre l’avion et venir se poser sur les plages paradisiaques de la Polynésie, territoire français aux antipodes de la France hexagonale perdu au milieu du Pacifique ? Et pourtant, l’on ne s’imagine pas que 60 % des Polynésiens vivent en dessous du seuil médian de bas revenu métropolitain ou que l’espérance de vie est inférieure à celle de la métropole en raison de l’importante prévalence des maladies cardiovasculaires avec les trois quarts de la population en surpoids.

Outre les maladies métaboliques non transmissibles, différentes pathologies communes aux pays tempérés présentent en Polynésie des spécificités dont la prise en charge et le raisonnement médical diffèrent parfois. En effet, en Polynésie où les îles s’étendent sur un territoire grand comme l’Europe, les retards de prise en charge sont fréquents et il peut parfois sembler difficile de renvoyer des patients malades dans leur île isolée. Certaines pathologies autrefois courantes en France comme le rhumatisme articulaire aigu y sévissent toujours, d’autres, comme la goutte, présentent un caractère rarement vu ailleurs en matière de prévalence ou de gravité.

Même si l’éloignement géographique l’a préservée de nombre de maladies tropicales dont le paludisme, la rage ou les animaux dangereux, la Polynésie présente un large éventail de maladies infectieuses comme les arboviroses, la leptospirose, la tuberculose, la lèpre ou l’angiostrongylose. Les infections cutanées sont très fréquentes et, avec elles, leur corollaire de complications dont les endocardites et les infections ostéoarticulaires. La mer, qui est omniprésente, expose aussi à quelques dangers comme l’intoxication par la ciguatéra et expose aussi à certains organismes marins.

La prise en charge se fait selon les standards modernes de la médecine grâce aux ressources de niveau européen permettant des possibilités diagnostiques et thérapeutiques inexistantes dans les autres états insulaires du Pacifique.

L’objectif de ce panorama est d’orienter dans leur pratique quotidienne les soignants en exercice ou voulant venir exercer en Polynésie française et également les praticiens prenant en charge des personnes au retour de Polynésie.

## Abstract (en)

Tahiti or the “myth of Paradise”, Bora Bora, “the Pearl of the Pacific”. Who has never wanted to take a plane and come and land on the heavenly beaches of Polynesia, a French territory at the antipodes of mainland France lost in the middle of the Pacific? However, we do not imagine that 60% of Polynesians live below the metropolitan low-income threshold or that life expectancy is lower than that of the mainland due to the high prevalence of cardiovascular diseases with three quarters overweight population.

In addition to non-transmissible metabolic diseases, various pathologies common to temperate countries present specificities in Polynesia, leading to sometimes different management and medical reasoning. Indeed, in Polynesia where the islands extend over an area of the size of Europe, delays in treatment are frequent and it can sometimes seem difficult to send sick patients back to their isolated island. Certain pathologies that were once common in France, such as acute rheumatic fever, are still prevalent there, while others, such as gout, are rarely seen elsewhere in terms of prevalence or severity. Even if the geographical distance has protected Polynesia from a number of tropical diseases including malaria or dangerous animals, this territory presents a range of varied infectious diseases including arboviruses, leptospirosis, tuberculosis and leprosy or angiostrongylosis. Skin infections are very common with their corollary of complications including endocarditis and osteoarticular infections. The sea, which is omnipresent, also poses certain dangers such as ciguatera poisoning and exposure to certain marine organisms.

Care is provided according to current medical standards thanks to European-level resources allowing diagnostic and therapeutic possibilities that do not exist in other Pacific island states.

The objective of this overview is to guide health care providers coming to or practicing in French Polynesia in their daily practice, but also practitioners taking care of people returning from Polynesia.

## Abréviations

**Table:** 

**AAC**	Autorisation d’accès compassionnel
**AAP**	Autorisation d’accès précoce
**ADEM**	Encéphalomyélite aiguë disséminée
**AFS**	Agence française du sang
**AgHBs**	Antigène HBs
**AIPS**	Association internationale pour la prévention du suicide
**AMM**	Autorisation de mise sur le marché
**ANSM**	Agence nationale de sécurité du médicament
**APF**	Assemblée de Polynésie française
**APHP**	Assistance publique - hôpitaux de Paris
**ASN**	Autorité de sûreté nucléaire
**ARASS**	Agence de régulation de l’action sanitaire et sociale
**ASAT/ALAT**	Aspartate aminotransférase/Alanine aminotransférase
**ATA**	Atmosphère absolue
**AVC**	Accident vasculaire cérébral
**CAR-T cells**	Lymphocytes T modifiés génétiquement
**CCSMIT**	Centre de consultation spécialisé en maladies infectieuses et tropicales
**CDC**	*Centers for disease control and prévention*
**CE**	Communauté européenne
**CHPf**	Centre hospitalier de Polynésie française
**CHIKV**	Virus chikungunya
**CHU**	Centre hospitalo-universitaire
**CIPAM & Cos**	Colloque international des plantes aromatiques, médicinales et cosmétopées
**CLCT**	Centre de lutte contre la tuberculose
**CNR**	Centre national de référence
**CPS**	Caisse de prévoyance sociale
**CRP**	Protéine C-réactive
**CTX**	Ciguatoxine
**DDB**	Dilatation des bronches
**DENV**	Virus de la dengue
**DOM-TOM**	Départements doutre-mer - Territoires doutre-mer
**DS**	Direction de la Santé
**EB**	Engagement de Berlin
**ECMO**	Oxygénation par membrane extracorporelle
**EHPAD**	Établissement d’hébergement pour personnes âgées dépendantes
**ESPER**	Évaluation et suivi des psychoses émergentes
**Évasan**	Évacuation sanitaire
**FAPF**	Forces armées de Polynésie française
**FPSS**	Fonds de prévoyance sanitaire et sociale
**HAS**	Haute autorité de santé
**HPLC**	Chromatographie en phase liquide haute performance
**HPV**	Papillomavirus humain
**HSH**	Hommes ayant des rapports sexuels avec des hommes
**HTA**	Hypertension artérielle
**IACS**	*International alliance for the control of scabies*
**ICPF**	Institut du cancer de Polynésie française
**IDE**	Infirmier(e) diplômé(e) d’État
**IgG**	Immunoglobulines G
**ILM**	Institut Louis Malardé
**IMV**	Intoxication médicamenteuse volontaire
**INR**	Rapport normalisé international
**INSEE**	Institut national de la statistique et des études économiques
**IRM**	Imagerie par résonance magnétique
**ISPF**	Institut de la statistique de la Polynésie française
**IST**	Infection sexuellement transmissible
**LAM**	Leucémie aiguë myéloïde
**MB**	Multibacillaire
**MI**	Médecine intégrative
**MOGAD**	Maladie du spectre des anticorps anti-MOG
**MTN**	Maladie tropicale négligée
**NMOSD**	Maladie du spectre de la neuromyélite optique
**MRC**	Maladie rénale chronique
**OMS**	Organisation mondiale de la santé
**OR**	Odds ratio
**ORL**	Oto-rhino-laryngologie
**PacELF**	*Pacific programme for the elimination of lymphatic filariasis*
**PB**	Paucibacillaire
**(RT) PCR**	Réaction en chaîne par polymérase quantitative après transcription inverse
**PET**	*Positron emission tomography*
**Pf**	Polynésie française
**POD**	Prise observée directe
**PrEP**	Prophylaxie pré-exposition
**PUI**	Pharmacie à usage intérieur
**RAA**	Rhumatisme articulaire aigu
**RCP**	Réunion de concertation pluridisciplinaire
**ROM**	Rifampicine, ofloxacine, minocycline
**SA**	Semaines d’aménorrhée
**SAMU**	Service d’aide médicale urgente
**SARM**	*Staphylococcus aureus* résistant à la méticilline
**SCA**	Syndrome coronarien aigu
**SGB**	Syndrome de Guillain-Barré
**SMUR**	Structures mobiles d’urgence et de réanimation
**SNC**	Système nerveux central
**SMPG**	Santé mentale en population générale
**(N)STEMI**	Infarctus du myocarde sans sus-décalage du segment ST
**TB (MDR)**	Tuberculose multirésistante
**TDS**	Travailleur(euse) du sexe
**TPHA**	Test d’hémagglutination pour *Treponema pallidum*
**TS**	Tentative de suicide
**UNV**	Unité neurovasculaire
**VDRL**	*Venereal Disease Research Laboratory*
**VHA, VHB, VHC, VHD**	Virus hépatotrope du groupe A, B, C, delta
**VIH/sida**	Virus de l’immunodéficience humaine/syndrome d’immunodépression acquise
**ZIKV**	Virus Zika

## Préambule

L’objectif de ce panorama est de présenter de façon large les pathologies tropicales infectieuses et non infectieuses les plus fréquentes et/ou les plus originales de Polynésie française, et de partager cette expérience avec les professionnels de santé de Polynésie ou d’ailleurs.

Après une première partie destinée à dresser le contexte général de la Polynésie française et de son système de santé, seront abordées les pathologies infectieuses regroupant les infections bactériennes, virales, mycobactériennes et parasitaires, puis les pathologies tropicales non infectieuses telles que le rhumatisme articulaire aigu, la ciguatéra puis les agressions par la faune. En raison de leur importante prévalence en Polynésie française, un chapitre sur les maladies non transmissibles et leurs spécificités y sera consacré puis des paragraphes concernant les particularités polynésiennes de la prise en charge de diverses pathologies seront proposés portant sur les pathologies pulmonaires, neurologiques, onco-hématologiques, psychiatriques, la grossesse ou la médecine hyperbare. Un chapitre relatif à la pharmacie et aux plantes polynésiennes d’intérêt médical termineront ce panorama avant que ne soient proposés des conseils aux voyageurs.

## Contexte général de la Polynésie française

**Rémi Mayan, Erwan Oehler**

### Aspects géographiques

**Figure 1:** Les trois grandes régions culturelles de l Océanie / ***The three major regions of Oceania*** 
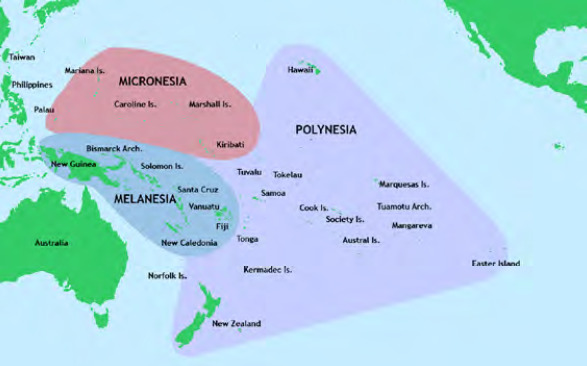

L’océan Pacifique est une vaste région qui couvre près d’un tiers du globe terrestre. Cet espace englobe de nombreux pays et états insulaires communément répartis en trois grandes régions décrites par les géographes européens du 19^e^ siècle en fonction de points communs linguistiques mais aussi d’opinions colonialistes largement démenties de nos jours [223]. Ces territoires sont la Mélanésie qui comprend la Papouasie-Nouvelle-Guinée, les îles Salomon, la Nouvelle-Calédonie, le Vanuatu et Fidji, la Micronésie qui comprend environ deux mille îles, dont les États fédérés de Micronésie, Palau, les îles Marshall et Kiribati et la Polynésie formant le « triangle polynésien » dont les sommets sont Hawaï (*Hawai’i* en polynésien) au nord, Rapa Nui (île de Pâques) à l’est et la Nouvelle-Zélande à l’ouest. Ce triangle polynésien comprend la Polynésie française (Pf), les îles Samoa, Tonga et Cook, Tuvalu, Tokelau, Niue, Wallis et Futuna (Fig. 1). On comprendra ainsi que la Polynésie française ne constitue qu’une partie de la Polynésie au sens large du terme.

La Pf se situe entre la ligne de l’équateur et le tropique du Capricorne sur une étendue équivalente au continent européen (Fig. 2). Elle est formée d’un ensemble de cent-dix-huit îles, dont 76 habitées, regroupées en cinq archipels (Fig. 3) :

- la Société, regroupant les îles du Vent (Tahiti, Moorea, Maiao) et les îles Sous-le-Vent (Raiatea, Tahaa, Huahine, Bora Bora et Maupiti);
- les Marquises au nord-est, divisées en deux groupes avec Nuku Hiva, Ua Pou et Ua Huka pour le groupe nord et Hiva Oa, Tahuata et Fatu Hiva pour le groupe sud;
- les Tuamotu constituées majoritairement d’îles basses (atolls) dont les principales sont Rangiroa, Fakarava, Makemo et Hao;
- les Gambier au sud est;
- les Australes, au sud, allant de Rimatara à Rapa.

La capitale, Papeete, est à 6 200 km de Los Angeles, à 7 950 km de Santiago du Chili, à 9 500 km de Tokyo et à presque 18 000 km de l’Hexagone.

Ces îles bénéficient de climats tropicaux et subtropicaux et de températures pouvant varier de 21 à 28 °C pendant la saison fraîche et sèche (hiver austral de mai à octobre) et de 24 à 31 °C pendant la saison chaude et humide (été austral de novembre à avril). Le climat généralement chaud et humide toute l’année invite à se baigner dans l’océan dont la température moyenne est de 27°C.

Il existe deux types d’îles : les îles hautes (dont certaines sont entourées d’un lagon et d’un récif) et les îles basses ou atolls, composées d’une bande corallienne circulaire séparant le lagon de l’océan (Fig. 4 et Fig. 5). Ces îles prennent toutes naissance au niveau d’un *hot spot* sous-marin à partir duquel naît un volcan donnant naissance à une île haute qui dérive du sud-est vers le nord-ouest. Ces îles hautes sont progressivement entourées d’une frange corallienne et, par des mécanismes d’érosion et d’enfoncement dans la plaque terrestre, le cône volcanique ne laisse plus place qu’à un atoll. L’image de l’archipel de la Société suivant cet axe est particulièrement édifiant depuis Tahiti culminant à 2 242 m jusqu’à Motu One qui n’a que quelques mètres de haut. Il y a donc une unité géologique à tous les archipels du Pacifique sud.

**Source : Https://commons.wikimedia.org/wiki/File:Pacific_Culture_Areas.png**

**Figure 2:** La Polynésie française dans la région du Pacifique / *French Polynesia in the Pacific région* 
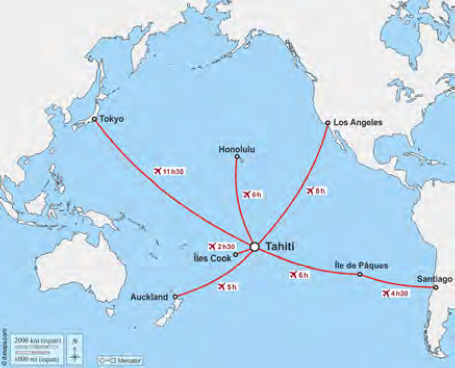

**Source : http://www.d-maps.com**

**Figure 3:** Répartition géographique des îles de Polynésie française, mise en perspective de la taille de l’Europe / *Geographical distribution of the islands of French Polynesia, putting the size of Europe into perspective* 
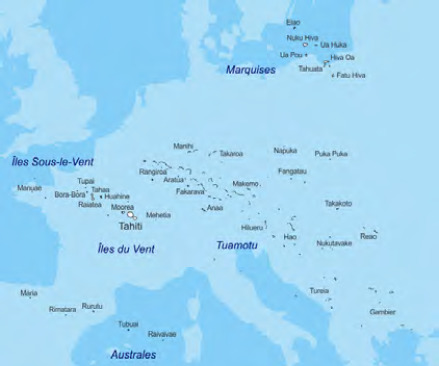

**Source**: **http://www.d-maps.com**

**Figure 4:** Rangiroa, atoll / *Rangiroa, atoll* (© E. Oehler) 
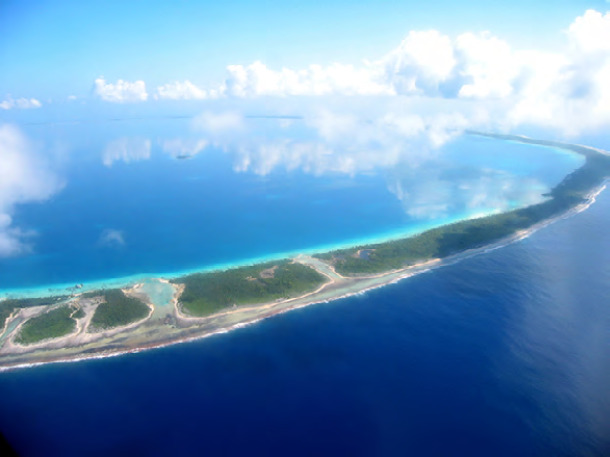

**Figure 5:** Tahiti, île haute / *Tahiti, high island* (© R. Boivin) 
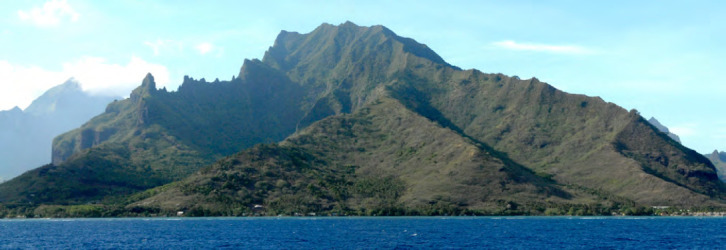

### Données démographiques

Sur une population totale de 278 786 habitants en 2022 (recensement ISPF, Institut statistique de la Polynésie française), 68 % vit sur l’île de Tahiti, le reste étant inégalement réparti entre les autres îles habitées (notamment sur l’archipel de la Société).

Cette population est plus jeune que celle de l’Hexagone (les 75 ans et plus représentaient 3,3 % de la population en 2021 contre 9,8 % en métropole) et l’espérance de vie moyenne est de 78,5 ans pour les femmes et 75,1 ans pour les hommes (contre 85,6 et 78,5) (Fig. 6) [126].

Sur la base de données déclaratives réalisées lors du recensement démographique de 1988, la population pouvait être divisée en trois principaux groupes ethniques : Polynésiens *(Maohi^1^)* (83 %), Européens (Popaa) (12 %) et Asiatiques *(Tinito)* (5 %) [155]. Un métissage est cependant observé dans chacun de ces groupes ethniques, le terme « demis » étant utilisé localement pour désigner les métis. Le français est la langue officielle et il existe sept langues polynésiennes *(reo maOhi)* dont certaines comportent plusieurs dialectes.

**Figure 6:** Pyramide des âges des habitants de Polynésie française en 1983 (A) et 2023 (B) / ***Age pyramid of inhabitants of French Polynesia in 1983 (A) and 2023 (B)*** 
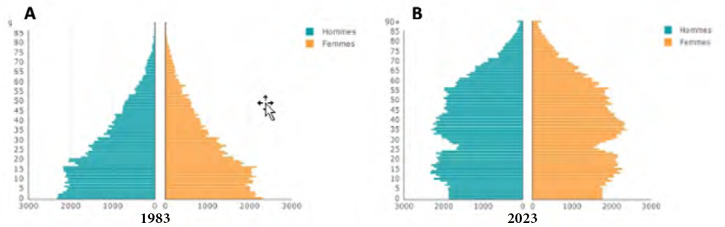

**Source: https://www.ispf.pf/themes/population**

Les premières mentions des îles polynésiennes remontent au 16^e^ siècle et à de grands navigateurs tels que Magellan, Mendaña ou Quiróqui furent parmi les premiers Européens à atteindre les Tumaotu et les îles Marquises. Wallis puis Bougainville posent le pied à Tahiti en 1767 et 1768 respectivement alors que Cook explore l’archipel de la Société dès 1769. L’arrivée des Européens fut marquée par un déclin rapide de la population polynésienne lié au contact avec des maladies infectieuses contre lesquelles elle n’était pas immunisée et sévissant sous la forme d’épidémies. Les bouleversements politiques occasionnés par les nouveaux arrivants seront également sources de guerres et de famines contribuant à la diminution de la population. C’est dans l’archipel des Marquises que cette diminution a été la plus intense et la plus durable, faisant craindre, à l’époque, une disparition complète de la population marquisienne. Une lente croissance démographique commencera entre le milieu et la fin du 19^e^ siècle en lien avec l’accroissement naturel de la population et de courants migratoires venus d’Europe et de Chine [128].

### La santé, une compétence propre du Pays

La Polynésie française est une collectivité d’outre-mer dotée d’un statut d’autonomie en application de l’article 74 de la Constitution française. La loi organique du 27 février 2004 a renforcé ce statut d’autonomie introduisant le concept de Pays d’outre-mer (POM) et l’usage est dorénavant dans le langage courant de parler du Pays pour désigner la collectivité politique polynésienne^2^. Depuis cette date, et pour simplifier la compréhension du statut d’autonomie, il est précisé que la Pf est compétente dans toutes les matières à l’exception de celles expressément attribuées à l’État (missions régaliennes). **La santé est par conséquent une compétence propre du Pays**.

Le financement de la santé repose principalement sur les cotisations prélevées par la Caisse de prévoyance sociale (CPS) aux salariés et patentés et sur des prélèvements indirects (impôts, versements au budget général du Pays, subventions de l’État, dons, legs et produits de participations). Il existe cinq régimes de sécurité sociale : le régime des salariés, le régime des non-salariés, le régime de solidarité, le régime militaire et l’Assurance maladie France pour les fonctionnaires d’État. Les soins curatifs sont rémunérés sur la base des soins prodigués à hauteur de 60 % par la CPS sauf en cas de longue maladie où la prise en charge est de 100 % (celle-ci est dénommée « carnet rouge », selon la couleur des « carnets de santé » à leur création). Les soins préventifs et les actions de promotion de la santé sont pris en charge *via* le Fonds de prévention sanitaire et sociale (FPSS), dotation globale annuelle votée à l’Assemblée de Polynésie française (APF) et issue de produits d’impôts indirects. Les établissements hospitaliers bénéficient d’une enveloppe annuelle globale fixée par l’APF (pas de financement selon l’activité).

L’organisation de la santé repose sur les orientations stratégiques portées par le ministère de la Santé en charge de la prévention et de la protection sociale généralisée, avec deux bras effecteurs : l’Agence de régulation de l’action sanitaire et sociale (ARASS) pour la planification, le contrôle, l’analyse financière et la réglementation, et la Direction de la Santé (DS).

Les deux acteurs majeurs de l’offre de soins sont la DS et le Centre hospitalier de Polynésie française (CHPf) :

- la DS emploie 1 200 agents dont 104 praticiens en 2022. Elle assure deux missions principales^3^ :
  -élaboration et mise en œuvre des politiques publiques de bien-être, de promotion et de prévention de la santé, priorisées selon les orientations du ministère de la Santé en charge de la prévention et de la protection sociale généralisée^45^. Pour exemple concret, certaines maladies comme l’angiostrongylose, la filariose ou le rhumatisme articulaire aigu sont inscrites sur la liste des maladies à déclaration obligatoire en Pf alors qu’elles ne le sont pas dans l’Hexagone;
  -gestion de 122 structures publiques de santé (quatre hôpitaux « de proximité », sept centres de consultations spécialisées, le Centre de la mère et de l’enfant, le Centre de santé environnementale, 20 centres dentaires, 13 centres médicaux, 17 dispensaires, 23 infirmeries et 43 postes de secours) répartis sur les cinq archipels en fonction de la densité de population et dont la localisation peut être consultée sur le site https://www.service-public.pf/dsp- direction-de-la-sante-publique/;
- le CHPf, hôpital d’environ 450 lits, emploie 150 praticiens et offre un plateau technique de qualité (seuls la chirurgie cardiaque et le PET-scanner n’y sont pas disponibles) et un soutien en soins spécialisés à la population, soit par accès direct *via* les urgences (40 000 visites par an), soit par transfert médical depuis des structures de soins primaires ou secondaires.

Trois établissements publics spécifiques complètent l’offre de soins :

- l’Institut Louis Malardé (ILM), laboratoire de biologie (analyses médicales et recherches en entomologie, biotoxines marines, maladies non transmissibles et maladies infectieuses à transmission vectorielle);
- l’Institut du cancer de Polynésie française (ICPF) (dépistage, anatomopathologie, registre du cancer);
- le *Fare Tama Hau* (maison de l’enfance et de l’adolescence).

Sur l’île de Tahiti, trois cliniques privées étoffent l’offre de soins en médecine, chirurgie et obstétrique ainsi que deux établissements de soins de suite et de réadaptation. Des centres de dialyse privés répartis sur les archipels (APAIR-APURAD et Dial’Isis) complètent le secteur de dialyse.

Le taux de rotation médicale moyen est important dans les archipels, celui-ci ayant été estimé à 251 % en 2015 [65]. L’isolement professionnel dans les îles éloignées associé à des astreintes continues, sont des facteurs de risque identifiés de l’absence de pérennisation de l’offre de soins sur le territoire.

### Évacuations sanitaires et SAMU

**Bertrand Remaudière, Rainui Richaud**

Dans chacune des îles habitées de Pf, une organisation sanitaire est mise en place grâce à la présence d’auxiliaires de santé, parfois d’infirmiers et plus rarement de médecins. Cette organisation gérée par la DS assure au quotidien quel que soit l’éloignement, une prise en charge globale des populations en santé primaire, secondaire et gestion des urgences. La télémédecine permet également de pallier ces contraintes géographiques par l’intermédiaire de téléconsultations réalisées principalement par téléphone mais aussi par des moyens plus modernes (consultations en visio) lorsque les connexions internet le permettent. En cas de nécessité médicale, les patients peuvent bénéficier d’une « évasan » (évacuation sanitaire), vers Tahiti principalement, pour le suivi de leur pathologie chronique ou en urgence en cas de problème aigu. L’évacuation sanitaire se fait par vol régulier pour le suivi des pathologies chroniques ou pour des pathologies aiguës compatibles avec un vol non médicalisé. Les plannings des vols réguliers sont variables selon les îles (de plusieurs vols par jour pour certaines îles à un vol par mois pour les plus isolées). Dans les îles non dotées d’un aéroport, les patients doivent d’abord rejoindre une île plus grande par bateau *(speedboat* ou bonitier - bateaux de pêche - ou navette maritime) avec des conditions de transport qui peuvent vite se dégrader en fonction des conditions météorologiques et de la durée du trajet. Certains patients nécessitant une aide technique ou humaine ont la possibilité d’avoir un accompagnant familial lors de ces évasans. La venue d’un patient des îles vers Tahiti nécessite le plus souvent de « regrouper » les consultations et examens et de lui proposer un hébergement dans une structure hôtelière financée par la CPS quand il n’a pas de famille sur place. Pour limiter le nombre d’évasans et assurer le suivi spécialisé des patients résidant en dehors de Tahiti, un système de « consultations spécialisées avancées » issu d’un partenariat tripartite entre la CPS, la DS et le CHPf a été créé, consistant à faire déplacer dans les îles un (ou des) spécialiste(s) du secteur libéral ou du CHPf. Plusieurs disciplines réalisent ces missions : spécialités médicales (cardiologie, angiologie, pneumologie, endocrinologie, néphrologie), spécialités chirurgicales (ophtalmologie, ORL, urologie) mais aussi gynéco-obstétricales (obstétricien et/ou sage-femme). Le praticien se déplace avec le matériel nécessaire (mallette et instruments médicaux, ordinateur, échographe…) et consulte dans une structure d’accueil (centre médical, infirmerie, poste de soins) selon un calendrier établi par la DS et l’ensemble des praticiens.

En cas de pathologies aiguës graves médicales, chirurgicales, pédiatriques, psychiatriques ou obstétricales nécessitant une prise en charge rapide et médicalisée, les décisions d’évasans urgentes sont prises par le médecin régulateur du Centre 15, souvent après discussion avec le demandeur et le médecin spécialiste. Ces évasans représentent un véritable défi humain, sanitaire, social et économique et impliquent une vraie chaîne de soins voire de survie qui nécessite de peser le bénéfice d’une surveillance sur place *versus* un transfert en urgence. Ces évacuations se font avec les moyens civils ou les moyens aériens des Forces armées de Polynésie française (FAPF). L’équipe médicale prenant en charge ces SMUR aériens se compose d’un médecin urgentiste et d’un infirmier (IDE) des urgences du CHPf, parfois une sage-femme ou un IDE spécialisé en néonatologie. Les délais de départ d’une évasan et le temps d’évacuation lui-même peuvent être très longs (de 1h à plus de 24h) en fonction des moyens disponibles, des autres demandes et de l’isolement de l’île. Ces prises en charge à grande distance et en vol sont donc très spécifiques et parfois périlleuses.

Les vecteurs de transfert sont multiples et choisis selon le degré d’urgence, la distance à Tahiti, les conditions de transfert, la disponibilité, l’horaire de vol, l’état des pistes, l’équipement des aérodromes et les conditions météorologiques. La dimension économique ne doit bien entendu pas être négligée, les coûts d’un rapatriement pouvant varier de 5 000 à plus d’un million de Francs Pacifique^6^ de l’heure. Sont ainsi disponibles comme vecteurs (Fig. 7):

- navette maritime : évasan en journée entre Tahiti et Moorea (sinon avion ou hélicoptère);
- vol par avion commercial : évasan entre les îles et Tahiti de patients ne nécessitant pas une prise en charge médicalisée;
- vol SAMU par avion : évasan de patients nécessitant une prise en charge médicalisée entre les îles éloignées et Tahiti, temps d’armement de 45 à 60 minutes, avec un prestataire privé (Beechcraft), l’Armée de l’air (CASA, C295) ou la Marine (Guardian);
- vol SAMU par hélicoptère : évasan de patients nécessitant une prise en charge médicalisée sur de courtes distances (<100 km) et des conditions météorologiques correctes avec un prestataire privé ou la Marine (Dauphin) pour les îles isolées sans aéroport;
- vol hélicoptère inter-îles aux Marquises : évasan des îles Marquises vers le Centre hospitalier de Nuku Hiva, disponible depuis 2020.
  Le Centre 15 reçoit ainsi près de 59 000 appels par an pour plus de 38 000 dossiers, permettant le déclenchement de près de 1 500 sorties SMUR et 1 000 évasans annuelles. L’organisation des évasans permet donc un maillage territorial efficace et unique avec les structures sanitaires insulaires dont la Direction de la Santé dispose, permettant un accès aux soins à l’ensemble des habitants.

**Figure 7:** Hélicoptère sur la *drop zone* du CHPf (A), Intérieur du Casa C295 (B), Casa C295 au-dessus d’un atoll des Tuamotu (C) / *Helicopter on the CHPf drop zone (A), Interior of the Casa C295 (B), Casa C295 above a Tuamotu atoll (C)*(© SAMU CHPf (A) Haut-Commissariat de Polynésie française / FAPF (B, C) 
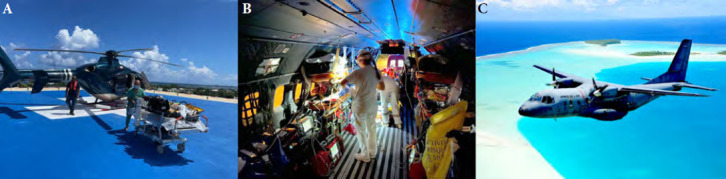

Des évacuations sanitaires internationales vers une structure de référence peuvent également être déclenchées pour la prise en charge des patients relevant d’activités non réalisées au CHPf. Ces évasans ne dépendent pas du Centre 15 mais des médecins de spécialité. Ce sont ainsi les patients nécessitant une chirurgie cardiaque, une chimiothérapie d’induction de leucémie aiguë fortement aplasiante, les grands brûlés ou encore les patients requérant la réalisation d’un PET-scan qui sont évasanés par des compagnies commerciales en vols réguliers vers Auckland (Nouvelle-Zélande, 5 heures de vol) ou l’Hexagone (22 heures de vol avec une escale technique aux États-Unis). Un accompagnement médical, paramédical et/ou familial est réalisé durant ces transferts. *A contrario,* en raison de la lourdeur logistique et financière de ces évasans internationales, des missions se sont progressivement développées au CHPf depuis plus de 30 ans, permettant de faire venir des praticiens experts dans leur domaine au sein d’une équipe et d’une infrastructure dotées du matériel et des équipements adéquats. Ainsi, des praticiens métropolitains ou étrangers (Néozélandais en cardiologie) viennent régulièrement réaliser des consultations et procédures complexes au CHPf dans le double but de limiter le nombre d’évasans internationales et de transmettre progressivement les compétences aux praticiens de Polynésie. Chaque année sont ainsi réalisées des missions de cardiologie interventionnelle (structurelle et rythmologique) et pédiatrique, de chirurgie orthopédique, urologique, ORL, de néphrologie, pédiatrie, génétique ou hématologie en provenance de métropole.

## Pathologies Infectieuses

### Infections bactériennes nfections de la peau et des tissus mous

**Erwan Oehler**

Les infections de la peau et des tissus mous sont sans doute les plus fréquentes des infections bactériennes en Polynésie française. Une étude réalisée en 2014 estimait l’incidence annuelle de l’érysipèle au minimum à 287/100 000, contre 10 à 100/100 000 en France hexagonale [139]. Les abcès et furoncles *(fēf^ē7^)* sont également fréquents sans que l’on ait de données épidémiologiques précises.

La prévalence de facteurs tels que l’obésité, le diabète, l’infection par la gale ainsi que le mode de vie (shorts, tongs) sont des éléments favorisant ces infections.

Les bactéries généralement en cause sont les streptocoques spp et le staphylocoque doré. Les streptocoques sont constamment sensibles aux pénicillines et présentent moins de 1 % de résistance aux macrolides; le staphylocoque doré est résistant à la méticilline (SARM) dans près de 30 % des cas traités à l’hôpital mais reste sensible aux autres antibiotiques antistaphylococciques [143]. Une étude non publiée réalisée au CHPf avait montré que près de 93 % des S. *aureus* impliqués dans des infections nécrosantes produisaient de la leucocidine de Panton-Valentine.

En milieu tropical, il n’est pas rare de voir des dermohypodermites bactériennes non nécrosantes à germes atypiques d’origine environnementale tels *Aeromonas hydrophila* (eau douce) ou *Vibrio vulnificus* (eau de mer) auxquels il faut penser en cas de situation évocatrice et/ou de mauvaise évolution sous antibiothérapie probabiliste antistreptococcique ou antistaphylococcique. ^<2<^

### Endocardites infectieuses

**Erwan Oehler, Rainui Richaud**

Les endocardites infectieuses ont une incidence annuelle trois fois plus élevée en Pf que dans l’Hexagone d’après l’analyse rétrospective de 105 endocardites infectieuses hospitalisées au CHPf entre 2015 et 2018 [26]. Dans cette étude, les patients étaient plus jeunes qu’en métropole et un patient sur trois présentait des antécédents de valvulopathie rhumatismale. Les staphylocoques (38 %) et streptocoques (30 %) étaient les agents étiologiques les plus fréquents. Soixante-treize patients avaient une indication théorique de prise en charge chirurgicale mais seuls 38 étaient « évasanés », les autres ne l’étant pas en raison d’une instabilité hémodynamique et/ou d’un terrain médical défavorable. Cette absence de prise en charge chirurgicale était corrélée à la mortalité qui était plus élevée que dans les pays occidentaux (37 % vs 24-26 %) [26].

### Leptospirose (ma’i mimi ‘iore)

**Erwan Oehler, Stéphane Lastère**

La leptospirose est une zoonose cosmopolite causée par un spirochète du genre *Leptospira,* dont il existe plusieurs espèces (notamment *Leptospira interrogans var Icterohaemorragiae* et *Australis* qui prédominent en Pf) et dont le principal réservoir est essentiellement représenté par les rongeurs [111]. Après une incubation de sept jours en moyenne, apparaît un syndrome pseudogrippal marqué par une fièvre et une polyalgie (céphalées, arthralgies et myalgies) avec des signes digestifs (diarrhées et vomissements). Un ictère et une suffusion conjonctivale peuvent également être observés de même qu’une atteinte rénale, hépatique, cardiaque (myocardite) ou pulmonaire (hémorragie intra-alvéolaire) dans les cas graves [23].

La leptospirose est une maladie à déclaration obligatoire en Pf. Une étude rétrospective avait montré une incidence annuelle de 42/100 000 habitants entre 2007 et 2017 en Polynésie française, contre 0,1 à 1/100 000 dans les zones tempérées. En 2023, 117 cas ont été déclarés avec 71 hospitalisations, 19 passages en réanimation et 2 décès [23,29,111].

**Figure 8:** Répartition de la leptospirose dans les îles de la Société [23] / ***Distribution of leptospirosis in the Society Islands [23].*** 
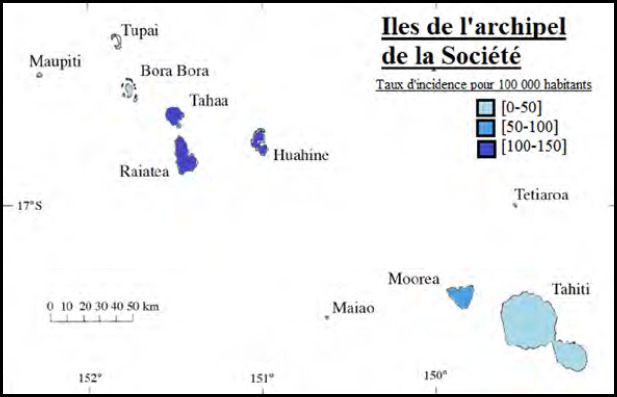

Le risque de leptospirose est plus élevé pendant la saison des pluies et dans certaines zones rurales et agricoles notamment Raiatea, Tahaa et Huahine dans les îles de la Société, toutes les îles Marquises et la presqu’île de Tahiti (Fig. 8 et Fig. 9).

**Figure 9:** Répartition des cas de leptospirose à Tahiti [23] / *Distribution of leptospirosis cases in Tahiti [23]* 
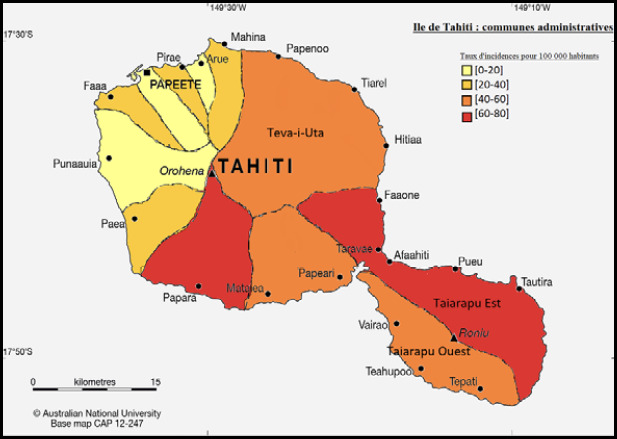

En Pf, les facteurs de risque de leptospirose sont le sexe masculin, l’âge et les conditions environnementales telles que l’élevage familial de porcs, la marche pieds nus, la randonnée et les activités nautiques dans les rivières [118]. Une étude sur 154 patients hospitalisés au CHPf entre 2018 et 2019 a montré que les leptospiroses sévères étaient associées à la présence d’un ictère, d’une hémoptysie, d’une suffusion conjonctivale, à l’absence d’hématurie et sur le plan biologique à une charge bactérienne élevée, à l’importance de l’augmentation de la CRP, de la procalcitonine et du rapport ASAT/ALAT. La réaction de Jarisch-Herxheimer n’était en revanche pas associée à la sévérité de l’infection. Cette étude montrait également que le score SPIRO était une alternative au score qSOFA pour l’évaluation précoce du risque de gravité y compris dans les régions de forte incidence [98].

### Brucellose

**Erwan Oehler**

La brucellose est une zoonose due à *Brucella* spp, bacille à gram négatif qui peut affecter la plupart des espèces de mammifères, notamment les ruminants et les suidés, et également les amphibiens et les cétacés. Du fait de la virulence et de la résistance dans l’environnement de *Brucella* (agent possible du bioterrorisme), les humains peuvent se contaminer par voie cutanéomuqueuse (contact avec des animaux ou carcasses d’animaux infectés), digestive (produits laitiers non pasteurisés) ou respiratoire (inhalation d’aérosols). La forme aiguë est marquée par une altération de l’état général, une fièvre ondulante avec sueurs profuses et des arthromyalgies. Une brucellose secondaire peut se voir en l’absence de traitement ou lors d’un traitement insuffisant avec formation de foyers infectieux isolés ou multiples formant des tableaux variés et non spécifiques donnant à la brucellose le nom de « maladie aux cent visages » [115].

En Pf, la brucellose est une maladie à déclaration obligatoire. Une vingtaine de cas ont été déclarés depuis 2000, la plupart en lien avec des abattoirs ou élevages de porcs industriels ou traditionnels. Une enquête a montré qu’en 2010 et en 2022 était observée une séroprévalence de 11 % dans les élevages et que la brucellose porcine y était épizootique *(B. suis* biovar 1) sans brucellose déclarée dans les autres espèces animales notamment chez les ruminants [97,121].

## Infections mycobactériennes

### Tuberculose (ma’i tūto’o)

**Lam Nguyen, Jérôme Debacre**

La tuberculose (TB) est endémique en Pf. En 1972, son incidence atteignait 180/100 000 habitants par an. Sa forte diminution au cours de la décennie suivante a probablement été multifactorielle, notamment grâce aux vastes campagnes de dépistage de la population par radioscopie mobile. L’incidence était d’environ 50/100 000 en 1983 et a ensuite fluctué entre 25 et 50/100 000 de 1983 à 1999. Une nouvelle baisse a été obtenue vers 2000 grâce à l’augmentation des ressources consacrées à la lutte contre la tuberculose. Depuis, l’incidence est restée aux alentours de 20/100 000 soit environ 50 nouveaux cas par an [105]. L’incidence a chuté en 2021 à 13,6/100 000, en lien probablement avec l’épidémie de Covid-19, et cette incidence est restée stable en 2023 à 12,4/100 000 avec 35 nouveaux cas [38]. La majorité des nouveaux cas est détectée à Papeete et dans sa banlieue (Tahiti Nui sur la Figure 10).

La lutte contre la tuberculose implique différents acteurs : le Centre de lutte contre la tuberculose (CLCT), le Centre de consultation spécialisé en maladies infectieuses et tropicales (CCSMIT), le CHPf, le secteur médical privé, les laboratoires, les services sociaux, les municipalités… Le CLCT voit en consultation le cas index pour faire le point sur la pathologie et les traitements et établit la liste des cas contact pour la réalisation d’un dépistage. La tuberculose est une maladie à déclaration obligatoire en Pf, les soins et le traitement sont gratuits pour la population ainsi que le dépistage des sujets contact. Les facteurs de risque d’acquisition sont les logements surpeuplés, les mauvaises conditions de vie et la mauvaise observance des traitements (CLCT, données non publiées).

La stratégie globale est principalement basée sur les directives de l’Organisation mondiale de la santé (OMS). Les principaux éléments de cette stratégie sont présents en Pf : engagement politique, laboratoires (microscopie et techniques moléculaires), approvisionnement en médicaments, système de surveillance assorti de traitements efficaces et traitement sous observation directe. Cependant, pour réduire davantage l’incidence de la tuberculose, des ressources supplémentaires semblent nécessaires. L’apparition de la tuberculose multirésistante (TB-MDR) obère depuis peu le succès de cette stratégie. Ce phénomène était plutôt rare et sporadique avant 2015, date à laquelle la première TB-MDR a été détectée dans une zone surpeuplée de Tahiti. Elle était liée à une mauvaise observance chez un patient jeune, difficile à tracer et à suivre. Un traitement approprié lui avait été proposé selon les recommandations du Centre national de référence (CNR) des mycobactéries à Paris utilisant fluoroquinolone, linézolide, aminoside, clofazimine et pyrazinamide pour une durée de 12 à 14 mois. Plusieurs fois défaillant dans son suivi médical, ce patient est décédé à son domicile de complications pulmonaires et est resté contagieux jusqu’à la fin. D’autres cas de la même famille ont été détectés et ont nécessité des efforts considérables pour leur traitement et une surveillance étroite mais en vain, puisque vingt cas de TB-MDR ont par la suite été diagnostiqués entre 2015 et 2022, non forcément liés épidémiologiquement au *cluster* initial, mais liés phylogénétiquement à la souche initiale [36,159]. Le fait que la circulation de cette souche n’ait pu être contrôlée depuis 2015 demeure un grave problème de santé publique en Pf.

**Figure 10:** Distribution géographique des nouveaux cas de tuberculose, 2018 / *Geographical* *distribution of new tuberculosis cases, 2018* 
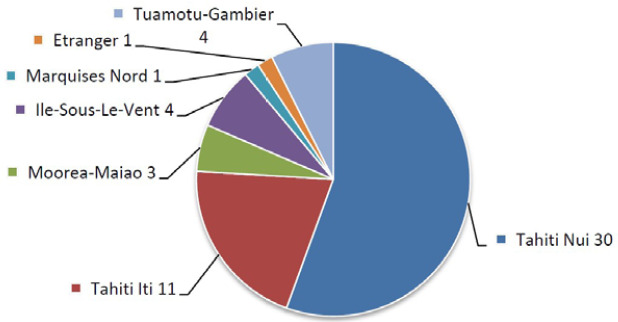

Source : https://www.service-public.pf/dsp/wp-content/uploads/sites/12/2019/04/Rapport- tuberculose-2018.pdf

### La lèpre ou maladie de Hansen (ma’i ‘ō‘ovi)

**Lam Nguyen, Erwan Oehler**

Bien que la maladie de Hansen (Fig. 11 et Fig. 12) existât avant l’arrivée des Européens dans les îles polynésiennes, c’est à partir du début du 19^e^ siècle et jusqu’en 1976 que les léproseries de Hiva Oa (Marquises), Reao (Tuamotu) et Orofara (Tahiti) ont accueilli les malades loin des zones surpeuplées.

**Figure 11:** Main de singe - lèpre tuberculoïde / *Monkey hand - tuberculoid leprosy* (© E. Oehler) 
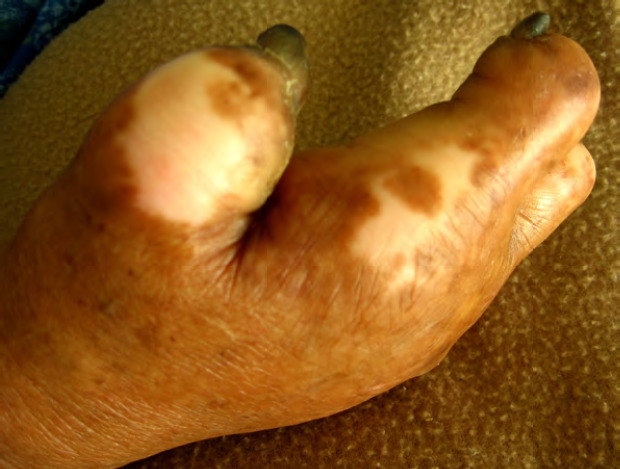

**Figure 12:** Lépromes de la jambe - lèpre lépromateuse / *Lepromas of the leg - lepromatous leprosy* (© E. Oehler) 
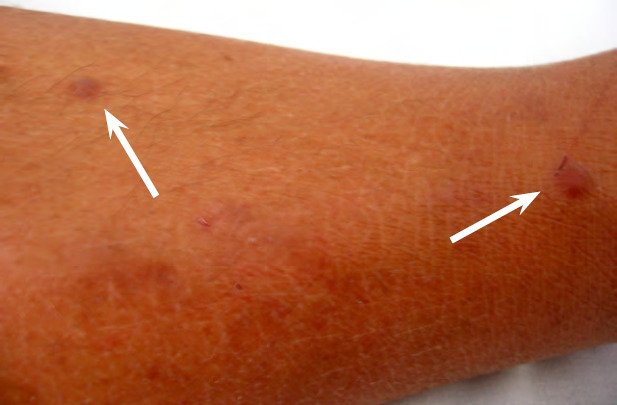

En Pf, la prévalence de la lèpre est inférieure au seuil d’élimination défini par l’OMS (< 1/10 000) depuis 1991. Cependant, de 1991 à 2022, aucune baisse supplémentaire de cette prévalence n’a été observée : elle est restée stable et a varié entre 0,14 et 0,87/10 000 (CCSMIT, données non publiées). En 2022, 10 cas ont été diagnostiqués, soit une incidence de 3,6/100 000, contrastant avec les 0,14/100 000 de 2021 et les 0,29/100 000 de 2020 [34]. Cette augmentation est probablement due à l’impact de la pandémie de Covid-19 sur l’accès aux services de santé et aux retards diagnostiques qui en ont résulté.

La plupart des patients sont traités et suivis en ambulatoire par le CCSMIT, l’unité référente pour la lèpre en Pf. Les traitements des patients multibacillaires (MB) et paucibacillaires (PB) utilisés en Pf sont légèrement différents de celui recommandé par l’OMS : la rifampicine est administrée quotidiennement en raison des contraintes géographiques qui rendent difficile la supervision mensuelle. Pour la forme MB, la rifampicine et la dapsone sont utilisées quotidiennement pendant 24 mois et la clofazimine quotidiennement pendant 12 mois. Pour la forme PB, le régime de traitement est basé sur la rifampicine et la dapsone quotidiennement pendant 6 mois. Dans la forme à lésion unique, le schéma ROM (dose unique de rifampicine, d’ofloxacine et de minocycline) recommandé par l’OMS est utilisé [171].

La chimioprophylaxie des foyers et des contacts proches a été mise en place en Pf en 2021, conformément aux recommandations de l’OMS. Il est intéressant de noter qu’historiquement, l’un des premiers essais de chimioprophylaxie de la lèpre avec une dose unique de rifampicine a été réalisé dans l’archipel des Marquises au milieu des années 1980 [48]. La prévalence de la lèpre au sein de la population marquisienne semble actuellement moins importante puisque les nouveaux cas viennent actuellement de l’archipel de la Société mais il est difficile d’en tirer des conclusions causales.

La lèpre est une maladie à déclaration obligatoire en Pf. Les soins et traitements ainsi que le dépistage des contacts et la chimioprophylaxie sont gratuits pour les bénéficiaires.

## Infections virales

### Hépatites virales chroniques B et C

**Bertrand Condat, Rémi Mayan, Stéphane Lastère**

La Pf est considérée comme une région de prévalence intermédiaire pour l’hépatite B. Une enquête réalisée en 1988 avait montré une prévalence du portage de l’AgHBs de 10,5 % dans l’archipel des Australes (avec un maximum de 27 % à Rapa, une île de cet archipel) [30]. Alors que la prévalence dans la population générale était estimée à 3 % en 2007 par le bureau régional de l’OMS pour le Pacifique sud-ouest, et 4,5 % sur les données de dépistage entre 2008 et 2016 (CHPf, données non publiées), une étude réalisée en 2019 avait montré une prévalence en baisse, de 1 % pondérée sur la population âgée de 18 à 69 ans mais qui restait très élevée (4 % ou plus) pour les archipels des Australes et des Marquises (3,8 et 6,5 % respectivement) et élevée (1,9 %) dans le groupe d’âge de 45-69 ans, justifiant un dépistage de ces populations [225]. La recherche d’anticorps anti-virus de l’hépatite delta (VHD) chez les porteurs de l’AgHBs était systématiquement négative dans l’étude de Lascols et la recherche d’ARN du VHD chez les porteurs d’AgHBs ne montrait aucun résultat positif dans l’étude de Cao-Lormeau *et al.* [136,225].

Depuis 1992, la stratégie de vaccination systématique inclut le vaccin contre l’hépatite B dès la naissance, l’objectif principal étant de réduire la transmission dans les premières années de vie. En 2014, le Bureau des programmes de prévention en maladies infectieuses, en collaboration avec l’OMS et le CDC d’Atlanta, a mené une enquête sérologique en milieu scolaire auprès de 1 176 enfants âgés de 6 ans : aucun d’eux n’a été testé positif à l’AgHBs [189]. Il s’agissait de la première étape vers la certification de l’absence de transmission parmi les enfants vaccinés contre l’hépatite B. Cette certification a finalement été reçue de l’OMS en 2018. La couverture vaccinale réelle en 2022 était de 99,6 % car le vaccin contre l’hépatite B est obligatoire pour les enfants scolarisés (Rémi Mayan, données non publiées).

Le fardeau de l’hépatite B en Pf reste encore lourd parmi les populations nées avant le programme de vaccination : l’étude menée par Lascols concernant 139 cas de carcinome hépatocellulaire en Pf, a montré que les facteurs de risque les plus fréquemment observés étaient l’obésité (60 %), la consommation d’alcool (56 %) et le virus de l’hépatite B (VHB) (51 %) alors que le virus de l’hépatite C (VHC) ne représentait que 4 % des cas totaux dont aucun cas autochtone. Par comparaison, en métropole, l’hépatite B était un facteur de risque de carcinome hépatocellulaire beaucoup plus rare (9 %) et l’hépatite C beaucoup plus fréquent (15 %) [136]. Pour l’archipel des Australes, le taux très élevé de CHC, un des plus élevés au monde, est associé dans près de 100 % des cas au VHB et notamment au génotype C qui explique en grande partie une telle incidence [224,225]. Seuls quelques cas d’hépatite C ont donc été rapportés à ce jour, la plupart acquis hors territoire. Sur les 1 857 prélèvements testés dans le cadre de l’étude *Mata‘ea*, la recherche d’IgG anti-VHC était systématiquement négative [225]. Cette prévalence quasi nulle, et ce malgré la culture du tatouage, est vraisemblablement liée à l’absence de consommation de drogues par voie intraveineuse et au nombre très limité de procédures invasives lourdes à l’époque où le risque transfusionnel existait.

### Hépatites virales aiguës A et E

**Erwan Oehler, Stéphane Lastère**

Le premier cas d’hépatite E autochtone a été décrit en 2015 [138]. Une enquête réalisée en 2018 auprès des donneurs de sang a indiqué une prévalence des IgG plus faible en Pf (7,7 %) qu’en France hexagonale (22,4 %), en lien possible avec les habitudes alimentaires polynésiennes [78]. Une épidémie d’hépatite A a été déclarée en 1995 avec, en deux ans, 2 072 cas signalés (1 % de la population), dont quatre décès par insuffisance hépatique fulminante [108]. La majorité des patients actuellement testés est cependant séropositive en IgG anti-VHA, reflet d’une probable circulation à bas bruit (CHPf, données non publiées).

Signalons que le diagnostic d’une hépatite virale aiguë A, B, C, E ou chronique B ou C doit obligatoirement être déclaré aux médecins de l’ARASS.

**Figure 13:** Évolution des cas déclarés de VIH par année en Polynésie française (réalisation : L. Nguyen, données non publiées) / *Evolution of reported cases of HIV per year in French Polynesia (production: L. Nguyen, unpublished data)* 
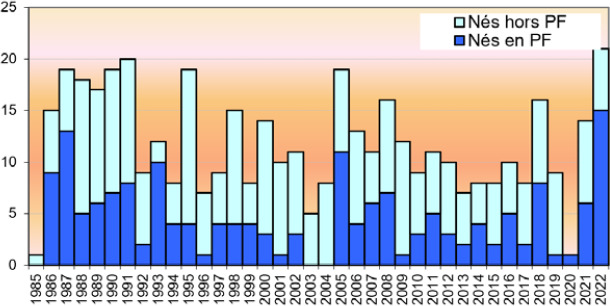

### VIH et IST

**Lam Nguyen, Rémi Mayan**

L’infection par le VIH a été introduite en Pf au début des années 1980. Les premières personnes infectées étaient des hommes ayant des relations sexuelles avec d’autres hommes (HSH) et des patients évacués vers la France pour une chirurgie spécialisée qui y ont été infectés par du sang contaminé (Lam Nguyen, données non publiées). Certaines de ces personnes font encore partie de la cohorte actuelle de patients séropositifs.

En 2022, 20 nouveaux^3^ cas ont été déclarés au système de santé et intégrés dans le registre polynésien du VIH. Parmi eux, 13 étaient nouvellement diagnostiqués, tous Polynésiens, les 7 autres venaient de l’Hexagone et étaient déjà connus séropositifs pour le VIH (Fig. 13). Fin 2022, 169 patients (sex-ratio 1,72) étaient suivis par le système médical polynésien et la cohorte principale (95 %) par le CCSMIT, unité référente pour le VIH/sida en Pf. Ces patients sont suivis à Papeete, principalement en ambulatoire ou au CHPf en cas de complication, par un médecin et trois IDE qui effectuent également des maraudes dans les lieux de prostitution de la zone urbaine de Tahiti. L’âge moyen de cette cohorte était de 50 ans (tant chez les hommes que chez les femmes), 23 % étaient HSH, 94 % étaient traités par antirétroviraux et, parmi eux, 92 % avaient obtenu un succès virologique (charge virale < 40 copies/ml).

L’incidence du VIH en Pf est estimée à 5/100 000 et la prévalence à 55/100 000 (9,7/100 000 chez les 15-24 ans) (CCSMIT, données non publiées). En moyenne sur les dix dernières années, approximativement la moitié des nouveaux cas annuels venaient de l’extérieur de la Pf, principalement de l’Hexagone, et se savaient déjà infectés.

À ce jour, comme pour l’hépatite C, aucune transmission par voie intraveineuse n’a été recensée en Pf du fait de la quasi-absence ou du très faible usage de drogues injectables sur le territoire. Par ailleurs, en dehors des premiers patients infectés dans l’Hexagone au début des années 80 *via* la transfusion lors des chirurgies lourdes, notamment valvulaires, il n’y a pas eu par la suite de contamination par transfusion recensée sur le territoire.

La prophylaxie préexposition (PrEP) a été mise en place en Pf à partir de 2017, notamment chez les HSH et les travailleurs/ses du sexe (TDS), sur la zone urbaine de Tahiti. Une soixantaine de personnes en ont bénéficié en 2022, bien qu’elle ne soit pas prise en charge financièrement par les services du Pays.

La prise en charge des personnes vivant avec le VIH en Pf présente quelques spécificités :

- les populations des îles, notamment des plus petites où « tout le monde se connaît », souffrent d’un manque de confidentialité pouvant affecter probablement le dépistage « anonyme »;
- la transidentité est une particularité de la région polynésienne qui se manifeste tôt, dès l’adolescence. Le terme māhū désigne les personnes du troisième genre qui ont un rôle spirituel et social au sein de la culture traditionnelle. Personnes de sexe masculin à la naissance avec une expression de genre féminine, ils sont souvent confondus avec les *raerae,* personnes transgenres parfois hormonées et/ou opérées, fortement associées à la prostitution [219];
- la prostitution est soumise à la même réglementation qu’en France et les TDS y sont plus difficilement accessibles à la prévention (via les réseaux sociaux…). Son « organisation » y est cependant différente, avec l’absence de réseau en lien avec la pègre ou d’autres organisations criminelles. La prostitution y est donc plutôt « artisanale » avec peu de proxénètes et des TDS travaillant pour leur propre compte. Il existe également des liens entre addictions (surtout méthamphétamine ou « ice ») et prostitution, cette activité permettant de financer cette consommation (250 000 XPF soit 2 000 € le gramme).

**Tableau I:** Données du système de surveillance des centres de dépistage des IST (réalisation : R. Mayan, données non publiées) / ***Data from fhe STI screening center surveillance system (production: R. Mayan, unpublished data)***

Données collectées CCSMIT et Institut Louis Malardé	2018 [25,26]	2021	2022 (1/01 - 31/07)
TOTAL de l’échantillon	ND	6 644 (100 %)	4780 (100%)
Nombre de PCR multiplex + (% des échantillons totaux)	2 324	3 850 (58 %)	2 697 (56 %)
*Chlamydia trachomatis* - *Ct* (% d’échantillons positifs)	13,1 %	459 (12 %)	323 (12 %)
*Mycoplasma genitalium* - *Mg* (% d’échantillons positifs)	ND	207 (5,3 %)	155 (5,7 %)
*Trichomonas vaginalis* - *Tv* (% d’échantillons positifs)	ND	185 (4,8 %)	131 (4,8%)
*Neisseria gonorrheae - Ng* (% d’échantillons positifs)	2,7 %	56 (1,4 %)	61 (2 %)
Co-infections		*Ng/Ct* : 30	*Ng/Ct* : 27
		*Ng/Mg* : 10	*Ng/Mg* : 15
		*Ng/Tv* : 3	*Ng/Tv* : 7
		*Ct/Tv* : 43	*Ct/Tv* : 31
		*Ct/Mg* : 60 (1,6 %)	*Ct/Mg* : 48 (1,8 %)
		*Ng/Ct/Tv* : 2	*Ng/Ct/Tv* : 6
		*Ng/Ct/Tv/Mg* : 1	*Ng/Ct/Tv/Mg* : 4
Sérologies de la syphilis (% des échantillons totaux)	3 626	2 800 (100 %)	2 096(100 %)
TPHA +	ND	155 (5,5 %)	119 (5 %)
TPHA + VDRL +	3,4 %	71 (2,5 %)	68 (3 %)

Il y a deux associations de lutte contre le VIH en Pf : « Agir contre le sida » et « Cousins/Cousines de Tahiti » regroupées sous la même bannière. Les bénévoles participent à la communication et à des animations dans les écoles, universités, évènements sportifs…

De façon plus générale, le nombre d’infections sexuellement transmissibles (IST)

diagnostiquées est plus élevé que le nombre de nouveaux cas de VIH (Tableau I). La prévalence de l’infection à *Neisseria gonorrheae* est de 2,8 % avec une absence de résistance aux fluoroquinolones et un taux de résistance à la tétracycline de 18 % contre 52-62,5 % en métropole. L’antibiorésistance de *Mycoplasma genitalium* est également plus faible que dans l’Hexagone : 14,7 % pour l’azithromycine et 1,32,6 % pour les fluoroquinolones (contre 42,1 % et 16,1 % respectivement) [24]. Des données plus récentes non publiées ont montré que les taux de résistance de *M. genitalium* aux macrolides étaient de l’ordre de 30 % (données de résistance aux fluoroquinolones en cours d’analyse).

En 2020, 3,5 % des personnes fréquentant les centres de dépistage avaient un test de syphilis positif (sex-ratio à 3). Les principaux facteurs de risque étaient les HSH et les personnes ayant déjà eu une IST. L’incidence était également plus élevée (8 %) chez les TDS, mais aucune conclusion n’a pu être tirée en raison de la faible taille de l’échantillon; elle atteignait cependant 35 % en 2018 dans cette même population [172].

### Covid-19

**Erwan Oehler, Rémi Mayan, Stéphane Lastère**

En 2019, a débuté la pandémie mondiale de Covid-19 qui n’épargna pas la Polynésie française où la fermeture des liaisons entre les îles et l’étranger du 11 mars au 3 juillet 2020 a permis de décaler l’arrivée du virus et de voir arriver la première vague épidémique avec la souche originale en août 2020. Durant cette vague, on notera un pic de 103 hospitalisations (dont 24 en réanimation alors que ce service dispose habituellement de 18 lits et 6 lits de soins intensifs).

La seconde vague due au variant Delta a débuté en août 2021 et a été la plus meurtrière : le pic d’incidence était de 3 300/100 000 habitants, le maximum de patients hospitalisés au même moment a été de 294 (60 % des lits du CHPf) dont 48 en réanimation au mois de septembre. Une étude de séroprévalence réalisée à l’issue de cette vague a montré que 57 % de la population polynésienne adulte entre 18 et 69 ans avait été en contact avec le Sars-CoV-2 et qu’un nombre important (71,6 %) n’avait jamais reçu de diagnostic d’infection par le Covid-19, illustrant une importante sous-estimation du nombre de cas. Les individus de sexe masculin, célibataires, fumeurs, présentant un antécédent d’allergie respiratoire ou vaccinés avaient par ailleurs moins de risque d’être séropositifs [154]. Il faut noter que pendant cette vague, le 19 septembre 2021, le plus long vol direct pour raisons médicales jamais rapporté a permis l’évacuation sanitaire de huit patients de réanimation vers Paris et de désengorger le service *ad hoc* [226].

La troisième vague liée au variant Omicron, a induit un pic d’incidence de 2 249/100 000 habitants le 20 février 2022. Le taux d’hospitalisation a été fortement réduit grâce à un taux de vaccination élevé dans la population générale (81 % de la population éligible avait reçu au moins deux doses) [193]. En complément, une campagne de vaccination obligatoire a concerné les travailleurs de santé et les professionnels du tourisme.

Fin juin 2022, une nouvelle augmentation de l’incidence a été liée à l’introduction des sous-variants Omicron BA.4 et BA.5 et moins d’une dizaine d’hospitalisations hebdomadaires a été enregistrée (Fig. 14). En fin d’année 2023, les sous-variants Omicron JN.1 et EG.5.1 ont été responsables d’une nouvelle augmentation de l’incidence et du nombre d’hospitalisations (Fig. 15). En milieu d’année 2024, les deux sous-lignages KP.2 et KP.3 issus de JN.1 étaient dominants [39].

**Figure 14:** Nombre de tests, nombre de cas confirmés et taux de positivité, semaine 29 2020 à semaine 32 2022 / *Number of tests, number of confirmed cases and positivity rate, week 29 2020 to week 32 2022* Source: https://www.service-public.pf/dsp/wp-content/uploads/sites/12/2022/08/BEH_N%C2%B0105.pdf 
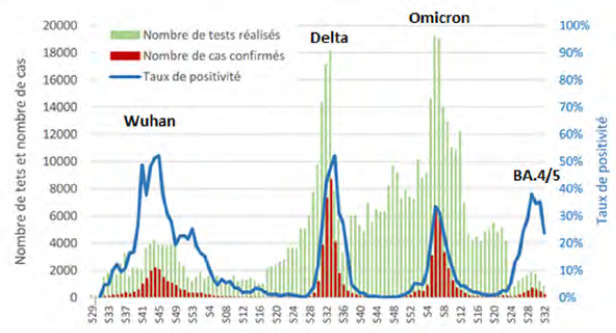

**Figure 15:** Nombre de cas confirmés, d’hospitalisations et de décès, semaine 19 2022 à semaine 01 2024 / *Number of confirmed cases, hospitalizations and deaths, week 19 2022 to week 01 2024* 
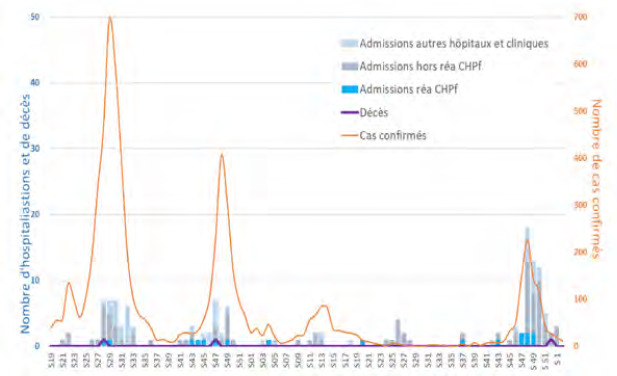

Source: https://www.service-public.pf/dsp/wp-content/uploads/sites/12/2025/04/ BSS12-S13-2025.pdf

La Pf adhère depuis novembre 2022 aux recommandations de l’OMS, faisant bénéficier la population d’un vaccin bivalent Covid en dose de rappel, pour les personnes vulnérables ou à risque, en parallèle du vaccin contre la grippe.

### Grippe

**Erwan Oehler, Stéphane Lastère, Rémi Mayan**

En Pf, la grippe présente comme particularité d’avoir deux périodes annuelles de circulation : décembre-janvier qui est une période de quatre semaines de vacances scolaires pendant lesquelles nombreux sont les voyageurs partant vers l’hémisphère nord et qui reviennent avec les souches de la grippe saisonnière sévissant dans cet hémisphère; juillet-août qui est une période de six semaines de vacances scolaires pendant laquelle on voit apparaître les variants de l’hémisphère sud.

En 2009, la pandémie grippale H1N1 a été introduite au mois de juin par un touriste venu des États-Unis d’Amérique avant d’essaimer à quasi l’ensemble des états insulaires du Pacifique. Durant cette épidémie dont le pic d’incidence a été observé durant le mois d’août avec un taux d’attaque de 16-18 %, 35 000 personnes ont consulté pour un syndrome grippal (soit un nombre de cas estimé entre 42 et 48 000), 13 patients ont été hospitalisés en réanimation et 7 patients sont décédés (taux de mortalité de 2,6/100 000) [87,133].

La campagne de vaccination annuelle contre la grippe saisonnière se déroule d’octobre à mars, le vaccin retenu par les autorités sanitaires étant celui de l’hémisphère nord en raison des données épidémiologiques annuelles et des liaisons commerciales plus fréquentes avec les pays de l’hémisphère nord (États-Unis et France).

Le vaccin est gratuit et prioritaire pour les personnes de 60 ans et plus, celles en longue maladie, les femmes enceintes, les personnes souffrant d’obésité (IMC ≥ 40), les personnels navigants (secteurs aérien et maritime), les agents touristiques accompagnant les groupes et tous les professionnels de santé.

## Arboviroses et infections transmises par les moustiques

**Van-Mai Cao-Lormeau**

### Principaux moustiques vecteurs en Polynésie française

**Hervé Bossin**

À ce jour, 15 espèces de moustiques ont été répertoriées en Pf. Six espèces sont endémiques, quatre espèces sont répandues à travers le Pacifique (espèces dites natives à l’instar *dAedes polynesiensis)* et d’autres très envahissantes (notamment *Aedes aegypti* et *Culex quinquefasciatus)* sont arrivées tardivement dans les îles du Pacifique. L’exploration et l’essor du commerce maritime ont en effet contribué à la propagation de ces deux dernières espèces à travers les régions tropicales et subtropicales du 17^e^ au 19^e^ siècle et, pour la région Pacifique, l’industrie baleinière a certainement contribué à la propagation de ces moustiques dès la fin du 18^e^ siècle [145]. Particulièrement abondantes à travers les îles peuplées du Pacifique, les espèces vectorielles *Aedes polynesiensis, Aedes aegypti, Aedes albopictus* et *Culex quinquefasciatus* jouent un rôle particulièrement important sur le plan médical, vétérinaire et économique :

• *Aedes polynesiensis* (Fig. 16 A) est aujourd’hui largement répandu à travers le Pacifique sud. Cette espèce serait originaire des Fidji ou des Samoa et aurait été disséminée plus à l’est, d’île en île, par le peuple polynésien il y a environ mille ans [21]. Présent dans tous les archipels de Pf, ce moustique peut pulluler de manière très intense dans les milieux végétalisés peu ou pas anthropisés des îles volcaniques (forêts en vallées et sur les collines) aux atolls les plus reculés. Les femelles de ce moustique sont exophiles et pondent leurs œufs dans une grande diversité de gîtes larvaires d’origine naturelle (cocos percés, trous d’arbres, terriers de crabes terrestres…) comme domestique (pneus usagés, fûts, bâches.). La diversité de ces gîtes larvaires souvent très dispersés rend la lutte contre ce moustique particulièrement difficile. Très actives au lever du jour et en fin de journée, les femelles *Ae. polynesiensis* se gorgent volontiers sur l’humain, mais aussi sur d’autres mammifères (chiens, rats, cochons) et sur les oiseaux. Vecteur de la filariose lymphatique *(Wuchereria bancrofti* var. pacifica) et de la filariose du chien *(Dirofilaria immitis),* cette espèce transmet également la dengue et d’autres arboviroses [203];

• *Aedes aegypti* (Fig. 16 B) est probablement l’un des moustiques le plus répandu au monde. Originaire d’Afrique de l’Est, il est aujourd’hui présent dans toutes les zones tropicales et subtropicales. Très inféodé aux humains, ce moustique anthropophile pond ses œufs dans des gîtes créés par ceux-ci. Il est reconnaissable à ses pattes zébrées de blanc et un profil en forme de lyre sur son thorax. Décrit pour la première fois à Tahiti sous le nom de *Ae. argenteus* à partir de spécimens collectés en 1904 l’espèce, alors introduite depuis peu, n’était observée que dans les zones les plus développées de Tahiti, Raiatea et Bora Bora [42,43]. Sa propagation dans les autres archipels de la Pf a été documentée au cours des décennies suivantes à travers diverses campagnes entomologiques [128]. Le moustique *Ae. aegypti* est aujourd’hui présent dans toutes les îles peuplées de la Pf. Il est le vecteur prépondérant des virus de la dengue, du Zika et du chikungunya dans le Pacifique et de bien d’autres arbovirus ailleurs dans le monde comme le virus de la fièvre jaune;

- *Culex quinquefasciatus* (Fig. 16 C) présente une robe marron-beige. Comme tous les *Culex*, les œufs sont pondus sous forme de nacelles flottantes et les larves se développent dans de l’eau parfois très chargée en matière organique (gouttières encrassées, fosses septiques.). Espèce envahissante, elle est présente dans toutes les îles de Pf et peut être très abondante en zone urbanisée comme en forêt. D’activité nocturne, les femelles piquent à l’extérieur comme à l’intérieur des habitations. Comme nombre d’espèces de moustiques qui ont envahi les îles du Pacifique, *Culex quinquefasciatus* est un vecteur connu de maladies infectieuses affectant les humains et la faune sauvage. Il a tout récemment été identifié comme le vecteur du paludisme aviaire (*Plasmodium relictum)* aux Marquises, affectant le Monarque de Fatu Hiva, une espèce d’oiseaux endémique en danger critique d’extinction (ILM/IRD, données non publiées). Même si cette espèce ne transmet actuellement aucune maladie humaine en Pf, c’est un vecteur potentiel d’autres agents comme le virus de la fièvre du Nil occidental, de la fièvre de la vallée du Rift, de l’encéphalite japonaise ou de la fièvre de Ross River [135] *;*
- *Aedes albopictus* ou moustique tigre est originaire d’Asie (Chine, Inde, îles de l’océan Indien) et déjà bien établi à Hawaï depuis le début du 20^e^ siècle. Ce n’est qu’à partir de la seconde moitié de ce siècle et notamment depuis ces 40 dernières années qu’il a connu une propagation planétaire facilitée semble-t-il par le commerce des pneus usagés, à travers l’Amérique du Nord, l’Amérique centrale et l’Amérique du Sud, les Caraïbes, le Pacifique et l’Europe dont la France hexagonale depuis 2004. Malgré sa propagation à travers la région Pacifique depuis la seconde guerre mondiale, ce moustique vecteur de la dengue, du chikungunya et de la dirofilariose est pour l’instant absent de la Pf et des autres territoires français du Pacifique et sa présence dans d’autres îles du Pacifique reste indéterminée faute de surveillance régulière [114]. Une surveillance accrue s’impose donc dans les principaux points d’entrée du territoire polynésien (ports et aéroport international) pour détecter et intercepter d’éventuels incidents d’introduction de cette espèce.

Par ailleurs, alors que le paludisme est un problème majeur de santé publique à travers toute la Mélanésie (à l’exception des îles Fidji), les îles polynésiennes en restent pour l’essentiel indemnes. Ceci est lié à une barrière écologique délimitée dans le Pacifique sud par la ligne de Buxton (courbe dont l’extrémité orientale est située par 170° de longitude est et 20° de latitude sud) qui passe au sud des îles Vanuatu et plus récemment au sud de la Nouvelle-Calédonie depuis que la présence de l’espèce *Anopheles bancroftii* a été découverte sur ce territoire [158,194]. Aucune espèce d’anophèles, vecteurs du paludisme, n’est présente dans les îles situées à l’est de cette ligne, pour des raisons semble-t-il écologiques liées à l’incapacité des anophèles de s’établir dans les écosystèmes insulaires polynésiens. Ainsi, en l’absence d’anophèles, les rares cas de paludisme décrits dans ces îles sont systématiquement le fait de cas importés.

La propagation des moustiques *Aedes* et des maladies vectorielles associées représente pour les prochaines décennies un risque sanitaire majeur pour la Pf comme pour l’ensemble du territoire français. Face à cet enjeu, les pouvoirs publics n’ont qu’un éventail d’outils limité, mis en œuvre en réaction aux épidémies et reposant le plus souvent sur l’usage de biocides qui présentent des risques pour l’environnement et la biodiversité et sont confrontés à des problèmes de biorésistances et de moindre acceptabilité sociale.

**Figure 16:** *Aedes polynesiensis* (A), *Aedes aegypti* (B), *Culex quinquefasciatus* (C) / Aedes polynesiensis *(A),* Aedes aegypti *(B),* Culex quinquefasciatus *(C)* (© Institut Louis Malardé, J. Marie) 
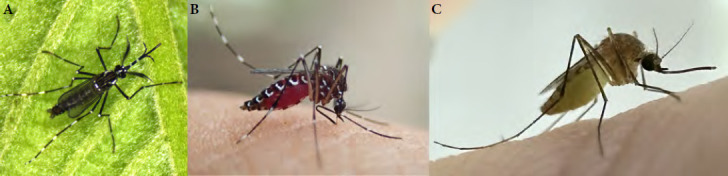

Durant la dernière décennie, la Pf a résolument investi, à travers les travaux pionniers menés par le laboratoire d’entomologie médicale de l’Institut Louis Malardé (ILM), dans le développement et l’évaluation de stratégies prometteuses en matière de surveillance et de lutte contre les moustiques vecteurs. Un réseau de surveillance de ces moustiques a ainsi été déployé conjointement avec la Direction de la santé à Tahiti (plus grand bassin de population en Pf) et Huahine (l’une des quelques îles de la Société où la transmission de la filariose lymphatique est encore active). Ce réseau a pour but d’actualiser les connaissances sur la diversité des espèces culicidiennes et de caractériser la répartition et l’abondance des espèces vectorielles les plus importantes sur le plan médical, vétérinaire et économique. Des succès ont par ailleurs été obtenus sur l’atoll de Tetiaroa avec la « Technique de l’insecte incompatible » par des lâchers de moustiques mâles porteurs de la bactérie *Wolbachia* qui ont permis d’éliminer de façon durable et respectueuse de l’environnement les nuisances causées par le moustique *Ae. polynesiensis.* L’ILM travaille également au développement et à l’évaluation régionale (Polynésie française, îles Cook, île de Pâques) de l’efficacité opérationnelle et épidémiologique d’approches intégrées associant mobilisation communautaire (élimination des gîtes à moustiques autour des habitations) et lâchers de moustiques mâles stériles pour lutter durablement contre *Ae. aegypti.* Ces travaux sont soutenus par l’OMS, le Pays et l’État. Le déploiement de ces stratégies de lutte innovantes à plus grande échelle (communes, îles entières) est aujourd’hui possible grâce au nouveau centre INNOVENTOMO de l’ILM, dont la construction vient de s’achever. Cette installation dédiée à la production industrielle de moustiques mâles stériles et financée au titre du contrat de projets État/Pays est la première installation de cette envergure en France et dans le Pacifique. Le centre INNOVENTOMO contribuera ainsi au développement des compétences et des capacités des pays et territoires insulaires du Pacifique en matière de lutte antivectorielle intégrée et innovante [179,235].

### Arboviroses

**Erwan Oehler, Rémi Mayan, Stéphane Lastère**

Les virus de la dengue (DENV), Zika (ZIKV) et chikungunya (CHIKV) sont transmis par les moustiques du genre *Aedes,* représentés principalement par les espèces *Ae. aegypti* et *Ae. polynesiensis*, ce dernier permettant une expansion des virus vers les îles les plus reculées où il est prédominant [204,205].

L’infection virale donne un tableau pseudogrippal non spécifique rendant le diagnostic difficile en cas de co-circulation virale. Le diagnostic est établi par RT-PCR avant J5 et par sérologie après.

La dengue, arbovirose la plus fréquente, est causée par un flavivirus dont les quatre sérotypes sont régulièrement présents en Pf, en alternance ou parfois en co-circulation (Fig. 17) [10]. La dernière épidémie remonte à 2019 avec l’introduction en Pf d’une souche de DENV-2 par des voyageurs en provenance de Nouvelle-Calédonie [12]. Au cours de cette épidémie, 3 330 cas ont été déclarés (dont 132 hospitalisations et 2 décès), dont plus de la moitié concernait des patients jeunes; le virus DENV- 2 avait en effet été responsable d’une épidémie entre août 1996 et avril 1997, puis avait circulé à un faible niveau de transmission jusqu’en décembre 2000 [37]. Depuis juillet 2021, il n’y avait plus de circulation active du virus de la dengue en Pf [11]. En 2023, un cas de dengue 3 importé en provenance d’Asie du Sud-Est a été confirmé en juin, puis en juillet un autre cas non sérotypé en raison d’une trop faible virémie, les deux sans cas secondaire [35]. Un premier cas autochtone de sérotype 2 a été identifié le 27 novembre 2023 déclenchant, comme lors des cas précédents, des actions de prévention et de lutte antivectorielle (recherche et élimination des gîtes larvaires et utilisation de traitements insecticides dans la zone où a séjourné le cas durant sa période de contagiosité) de la part du Centre de santé environnementale de la DS. Ces actions ont pour le moment permis d’éviter une diffusion épidémique du virus puisqu’au milieu du mois de juillet 2024, il n’était recensé que 95 infections dont 12 hospitalisations [40].

**Figure 17:** Circulation des quatre virus de la dengue, du Zika et du chikungunya en Polynésie française de 1944 à 2021 (les périodes épidémiques et interépidémiques sont respectivement foncées et claires) [11] */ Circulation of the four dengue viruses, Zika and chikungunya in French Polynesia from 1944 to 2021 (the epidemic and interepidemic periods are dark and light respectively) [11]* 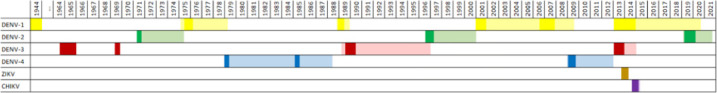

ZIKV est également un flavivirus qui avait peu retenu l’attention des scientifiques avant 2013. En effet, c’est à cette date qu’une épidémie de grande ampleur s’est déclarée en Pf entre octobre 2013 et mars 2014 (Fig. 17), dans une population immunologiquement naïve, avec 32 000 cas symptomatiques répondant à la définition de cas suspects, soit 11,5 % de la population [148,166]. Des études ultérieures ont montré une séroprévalence de 49 % dans la population et jusqu’à 66 % chez les enfants scolarisés [13,116].

L’intensité des symptômes était globalement légère à modérée sans nécessité de consultation médicale dans la plupart des cas. Cette vague polynésienne a, par contre, été marquée par une « épidémie » de syndromes de Guillain-Barré (SGB) avec 42 patients atteints en l’espace de quatre mois, contre un ou deux cas par an habituellement. La lourdeur de la prise en charge de ces patients a entraîné la saturation du service de réanimation du CHPf [45,178]. Des anomalies de l’embryogenèse avec notamment une majoration du risque de microcéphalie ont également été constatées lors de cette épidémie avec huit cas de microcéphalie observés au cours de cette période en Pf. Une étude réalisée à la suite de cette épidémie a permis de modéliser le risque de microcéphalie qui était estimé à 95 cas pour 10 000 femmes infectées au cours du premier trimestre, tandis que la pré valence était de 2/10 000 nouveau-nés avec un rapport de risque de 53,4 [49]. D’autres anomalies du développement cérébral fœtal ont par la suite été mises en évidence avec une probable sous-estimation du nombre des cas observés en Pf [129,220]. Aucun cas de Zika n’a été signalé en Pf depuis l’épidémie de 2013-2014.

CHIKV est un alphavirus de la famille des *Togaviridae* dont la présentation clinique est marquée par des douleurs articulaires intenses à la phase aiguë et pouvant persister plusieurs mois. La Pf a subi une épidémie entre 2014 et 2015 (Fig. 17) avec près de 69 000 cas (un quart de la population), plus de 900 hospitalisations dont 64 en réanimation et 32 décès [15,132]. Comme pour l’épidémie de ZIKV, celle-ci a été marquée par une augmentation de l’incidence du SGB (9 cas en six mois), également dans une population immunologiquement naïve [176]. À la fin de cette épidémie, une enquête de séroprévalence a montré la présence d’anticorps anti-CHIKV chez près de 75 % de la population, ce qui explique probablement l’absence de nouveaux cas depuis 2015 [14].

La lutte antivectorielle fait partie de l’arsenal stratégique contre les arbovirus. Elle fait l’objet d’une information de la population pour la lutte contre les gîtes larvaires et la protection individuelle. En période épidémique, la déclaration obligatoire des cas confirmés d ’arbovirus permet de pulvériser des insecticides dans les quartiers sources.

### Filariose lymphatique (ma’i marin)

**Rémi Mayan, Jean-Marc Ségalin, Erwan Oehler**

La filaire du genre *Wuchereria bancrofti* est responsable de 90 % des cas de filariose lymphatique à travers le monde. Ce parasite existe sous deux formes morphologiquement identiques qui se distinguent par la périodicité de circulation des microfilaires dans le sang périphérique. La forme classique, de périodicité nocturne, est transmise par des moustiques d’activité nocturne, généralement du genre *Culex,* notamment *Cx. quinquefasciatus.* En Pf et dans les îles du Pacifique sud, seule la variété pacifica de *W. bancrofti* circule, transmise par le moustique diurne *Ae. polynesiensis*. Cette variété, apériodique, permet de s’affranchir d’horaires spécifiques pour la réalisation de bilans biologiques.

La filaire adulte ou macrofilaire, qui vit dans le réseau lymphatique, est seule responsable des symptômes; ses larves ou microfilaires participent à la transmission de la maladie. Les manifestations aiguës quant à elles sont liées aux phénomènes inflammatoires provoqués par les macrofilaires : lymphangite centrifuge des membres ou des seins, adénite superficielle ou profonde, épisodes génitaux (orchi-épididymite, funiculite, lymphangite du scrotum, du pénis ou de la vulve). Les manifestations chroniques apparaissent après dix à quinze ans d’évolution et sont liées à la perturbation du système lymphatique par les macrofilaires : adénite chronique, adénolymphocèle, lymphœdème pouvant aller jusqu’à l’éléphantiasis des membres (essentiellement des membres inférieurs) ou des organes génitaux externes (Fig. 18), chylurie en cas de fistule entre les canaux lymphatiques et les voies urinaires… [53].

La sérologie est le premier test à se positiver mais elle ne permet pas de distinguer une infection active, une infection ancienne ou un simple contact. Elle est peu spécifique et donc peu utilisée en pratique en dehors d’études épidémiologiques. La recherche de l’antigène filarien circulant permet le diagnostic de filariose chez un patient présentant des macrofilaires, que le patient soit symptomatique ou non. Il est actuellement recherché lors d’un bilan d’hyperéosinophilie, chez les patients présentant des manifestations chroniques ou dans leur entourage, ou lors d’un séjour prolongé (> 3 mois) dans les zones où la prévalence reste élevée. C’est également l’examen de référence pour le pilotage des campagnes de distribution de masse contre la filariose lymphatique. La recherche de microfilaires sanguines (microfilarémie) constitue l’examen de référence avec une très bonne spécificité mais une faible sensibilité en microscopie optique. Lorsqu’il est positif, ce test est associé au risque de transmission de la maladie, à condition que le vecteur soit présent. De façon plus exceptionnelle, le diagnostic peut être réalisé sur une biopsie ganglionnaire effectuée en cas de suspicion d’hémopathie ou sur une échographie scrotale qui permet d’observer les vers adultes en cas d’épanchement (signe de la « danse filarienne ») [53,167].

**Figure 18:** Éléphantiasis filarien, Tahiti, 2019 / *Filarial elephantiasis Tahiti, 2019* (© E. Oehler) 
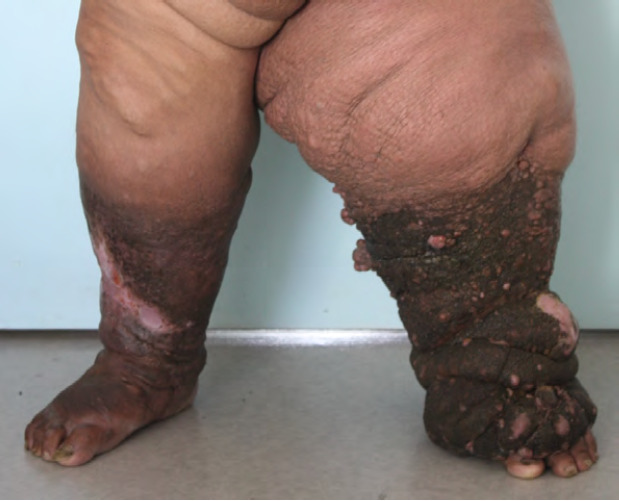

L’arsenal thérapeutique comprend des molécules microfilaricides et une molécule macrofilaricide. Les traitements microfilaricides permettent la destruction des microfilaires et ainsi de casser la chaîne de transmission dans la population. Les trois molécules utilisées sont la diéthylcarbamazine (DEC), l’albendazole et l’ivermectine, avec des combinaisons qui varient selon les régions car la DEC ne doit pas être utilisée en zone d’endémie de loase ou d’onchocercose. Si la combinaison DEC/ albendazole a aussi un effet macrofilaricide partiel, l’effet le plus significatif est obtenu en utilisant la doxycycline. Du fait de la présence de *Wolbachia,* bactérie endosymbiotique, l’utilisation de cet antibiotique permet la destruction des vers adultes moyennant un traitement de plusieurs semaines qui est incompatible avec une stratégie de masse. Compte tenu de la prévalence mondiale (120 millions de personnes atteintes selon l’OMS), un programme d’élimination a été initié avec la mise en place en 1999 d’un Programme régional pour l’élimination de la filariose lymphatique dans le Pacifique (PacELF). Ce programme a permis la distribution d’un traitement prophylactique de masse sans supervision (DEC et albendazole) chaque année en Pf à partir de 2000. En 2008, une étude de prévalence à l’échelle de la Pf a montré la persistance d’une prévalence de l’antigène filarien de 11,3 % amenant, à partir de 2010, à une nouvelle stratégie de distribution de masse basée sur le traitement supervisé (prise directe observée ou POD) avec pour objectif une prévalence inférieure à 1 % qui correspond au seuil d’élimination de la filariose lymphatique défini par l’OMS [164]. Depuis, la diminution de l’aire de distribution de *l’Ae. polynesiensis* (principalement dans les fonds de vallées de Tahiti où vivent peu de personnes et dans quelques rares îles) et la bonne couverture médicamenteuse ont permis, à partir de 2016, d’arrêter la distribution de masse dans la majorité des archipels, à l’exception de Huahine (îles Sous-le-Vent, Société) et les îles Marquises du sud où la prévalence de l’antigénémie filarienne restait supérieure à 1 % [81].

Le protocole de traitement de masse a finalement été modifié en 2022 avec l’introduction de l’ivermectine en plus de la DEC et de l’albendazole, conformément aux directives de l’OMS de 2018 [181]. On s’attend à ce que la combinaison des trois molécules et la campagne de lutte antivectorielle favorisent l’élimination de la filariose.

La filariose lymphatique est responsable d’une importante morbidité avec des patients qui consultent devant l’augmentation de volume d’un membre ou plus fréquemment pour des complications infectieuses telles que des dermohypodermites bactériennes compliquant ces lymphœdèmes. La prévention des complications infectieuses repose sur des mesures d’hygiène (lavage au savon et séchage soigneux, lutte contre l’œdème) et la surveillance clinique à la recherche d’infections bactériennes et/ou fongiques. La compression veinolymphatique est le traitement le plus efficace au stade précoce pour éviter

**Figure 19:** Gale profuse avec éléments hyperkératosiques / *Profuse scabies with hyperkeratotic elements* (© E. Oehler) 
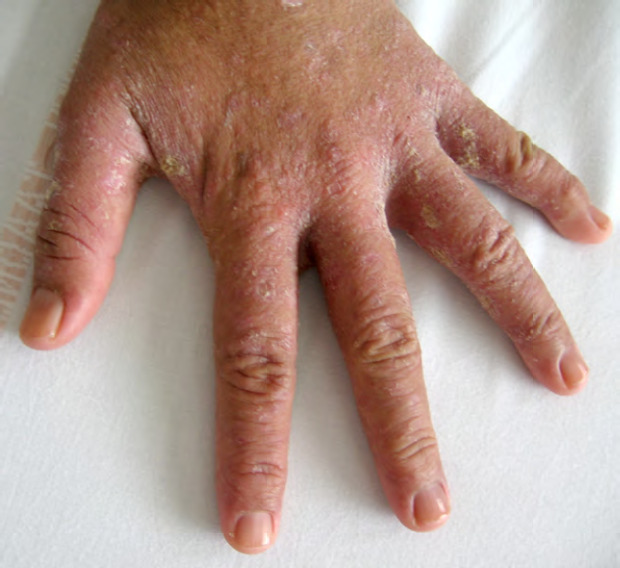

l’évolution vers l’éléphantiasis qui constitue le plus souvent une impasse thérapeutique. Les traitements médicamenteux antiparasitaires sont généralement inutiles à ce stade car ces complications tardives induites par des lésions des canaux lymphatiques surviennent souvent après la mort naturelle des parasites. Quant au traitement chirurgical du lymphœdème, il est efficace sur les formes scrotales mais les résultats au niveau des membres sont souvent décevants car très délabrants et à risque de difficulté de cicatrisation en lien avec l’hyperdébit lymphatique. Le traitement des anastomoses entre les gros vaisseaux lymphatiques et l’arbre urinaire, responsables d’une chylurie, peut également être accessible sous certaines conditions à la chirurgie.

## Infections parasitaires

### Gale

**Erwan Oehler, Rémi Mayan**

Comme dans de nombreux pays, la gale représente un problème de santé publique notable en Pf. Fort heureusement, le Département des maladies tropicales négligées de l’OMS a officiellement reconnu la gale comme une maladie tropicale négligée (MTN) en 2017. En outre, 2019 a vu la création de l’Alliance internationale pour le contrôle de la gale (IACS) puis en 2021 a été créé le Programme mondial de contrôle de la gale *(World Scabies Program).* En Pf, l’incidence de la gale est inconnue. En 2013, une étude estimait le taux d’incidence dix fois plus élevé que dans l’Hexagone (Direction de la veille sanitaire de Pf, données non publiées). Les gales diffuses ou hyperkératosiques sont également fréquentes (Fig. 19). Une enquête organisée et financée par le Programme mondial de lutte contre la gale a été réalisée en novembre 2023 afin d’établir sa prévalence en Pf ainsi que dans d’autres États insulaires du Pacifique (données soumises pour publication). En parallèle, des données non publiées relatives aux consommations d’antiscabieux topiques et oraux en 2023 ont permis d’estimer une fourchette d’incidence entre 4 et 9 % de la population.

La stratégie de contrôle doit, par ailleurs, comporter le diagnostic et le traitement des maladies bactériennes de la peau fréquemment associées à la gale et liées au rhumatisme articulaire aigu et/ou à la cardiopathie rhumatismale.

### Méningite à éosinophiles

**Erwan Oehler, Stéphane Lastère, Raphaël Buon**

L’angiostrongylose liée à *Angiostrongylus cantonensis* est la méningite à éosinophiles d’origine parasitaire la plus fréquente.

Le Livre jaune du CDC définit la transmission comme suit : « Diverses espèces de rats sont les hôtes définitifs du parasite, connu sous le nom de ‘ver pulmonaire du rat’. Les parasites des rats n’infectent que les escargots et les limaces, qui sont les hôtes intermédiaires. Des larves infectieuses ont également été trouvées chez des hôtes paraténiques (de transport), tels que des crevettes d’eau douce, des crabes et des grenouilles, qui s’infectent en consommant des escargots et des limaces infectés. La transmission aux humains se produit par l’ingestion d’hôtes intermédiaires ou paraténiques infectés ou par la consommation de produits crus ou de jus de légumes contaminés » [50].

Le tableau clinique est aspécifique et débute par un syndrome pseudogrippal avec un syndrome méningé inconstant et souvent bénin. Les signes subjectifs (sensations de brûlure, paresthésies diffuses et non systématisées des extrémités), présents dans un tiers des cas, sont parfois évocateurs du diagnostic en zone d’endémie. La ponction lombaire permet d’orienter le diagnostic en montrant un liquide hypertendu et clair avec une hypercellularité comprenant 20 à 70 % d’éosinophiles. La PCR et/ou la sérologie *Western blot* sur le liquide cérébrospinal le confirment [175].

Signalée dans tous les territoires français d’outre-mer, son incidence est la plus élevée en Pf [17,68,69,70,75,86,153].

Une étude rétrospective polynésienne avait recueilli 42 cas d’infection avec sérologie positive entre 2000 et 2012, mais ce chiffre a significativement augmenté à partir de 2015 (entre 5 et 15 cas déclarés chaque année) depuis la mise en place d’une technique de PCR maison au CHPf. La majorité des cas diagnostiqués sont des adultes (80 %) d’origine polynésienne avec un sex-ratio de 1,5 [41,177]. Les formes sévères, définies par des troubles de la conscience, des convulsions ou la présence de signes neurologiques déficitaires, concernaient un tiers des patients avec une récupération complète dans tous les cas dans un délai variable et aucun décès signalé. Aucun facteur de risque prédictif de forme grave n’avait été identifié dans l’analyse multivariée [177].

Le traitement est symptomatique et la ponction lombaire diagnostique est également thérapeutique en diminuant la pression intracrânienne tandis que les corticoïdes semblent réduire la sévérité des céphalées, la durée des symptômes et la nécessité de réitérer les ponctions lombaires soustractives. Les antihelminthiques comme l’albendazole pourraient aussi être associés même si leur utilisation est controversée en raison du risque théorique d’inflammation du système nerveux central par libération d’antigènes parasitaires pro-inflammatoires [177].

En Pf, les facteurs de risque de contamination sont la consommation de plats locaux à base de *taioro* ou *mitihue* (noix de coco fermentée à l’eau de mer et têtes de crevettes d’eau douce crues) (Fig. 20), la consommation de crabes et d’escargots crus (en particulier l’escargot géant africain *Lissachatina fulica* (Fig. 21) introduit en Pf à la fin des années 1960 et porteur du parasite) ainsi que la consommation de salades et de plantes non lavées [93,177].

En Pf, des mesures préventives sont recommandées : les crevettes peuvent être consommées crues à condition d’avoir été préalablement congelées pendant au moins 48 heures, les légumes du jardin, potentiellement en contact avec les escargots infestés, doivent être lavés soigneusement [175].

**Figure 20:** Poisson cru fermenté et mitihue / *Raw fermented and mitihue fish* (© E. Oehler) 
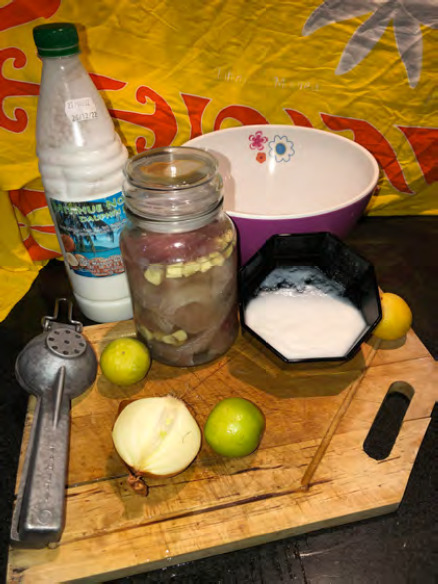

**Figure 21:** *Lissachatina fulica* / Lissachatina fulica (© E. Oehler) 
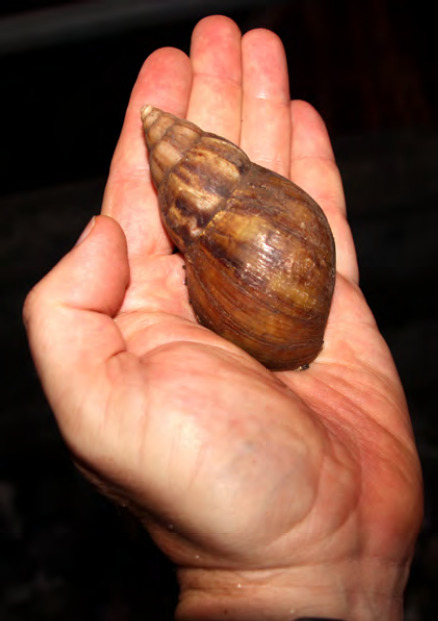

### Autres parasitoses

**Erwan Oehler**

*Tropical Diseases Annual Report Form)* qui est un document standardisé utilisé par les pays participant au programme mondial de l’OMS sur les MTN. La parution en 2024 du rapport sur « Les maladies tropicales négligées en Polynésie française » (Rémi Mayan) permet d’avoir une vision objective de la situation en Pf de ces maladies (dengue, chikungunya, gale, lèpre, filariose lymphatique et géohelminthiases).

**Figure 22:** *Larva migrans* / Larva migrans (© H Pujol) 
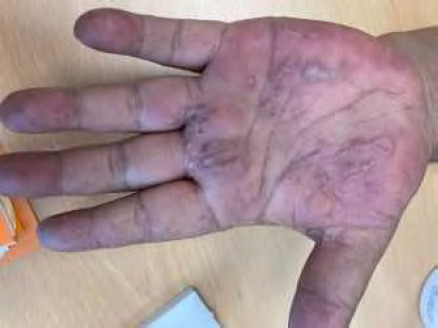

L’amœbose (ou amibiase) est une infection parasitaire cosmopolite associée au péril fécal dont la prévalence est plus élevée dans les régions tropicales et intertropicales. Elle est liée à des amibes du genre *Entamoeba* dont le seul pathogène est *Entamoeba histolytica histolytica.* L’incubation est variable mais habituellement de plusieurs mois voire années. On distingue généralement les formes coliques subaiguës voire chroniques, associées à des douleurs abdominales et des selles pâteuses et la forme hépatique d’apparition brutale avec une fièvre élevée et une hépatomégalie douloureuse en lien avec le développement d’abcès (des signes d’irritation pleurale peuvent également être observés lorsque l’abcès hépatique est à développement supérieur). Le diagnostic de l’amœbose intestinale repose sur l’examen parasitologique des selles ou la PCR qui est plus sensible. La sérologie est utile dans l’amœbose hépatique dans laquelle le parasite n’est qu’exceptionnellement mis en évidence lors de la ponction d’un abcès hépatique qui ramène un pus amicrobien couleur chocolat, pathognomonique de l’infection. Il n’existe aucune donnée épidémiologique récente relative à l’amœbose en Pf mais des publications anciennes rapportaient respectivement une série de 16 patients en 2 ans en 1984 et 42 patients en 6 ans en 1992, alors qu’une autre publication de 1992 montrait que 43 % des abcès hépatiques ponctionnés étaient d’origine amibienne [102,103,151]. Depuis 2012, le nombre médian d’hospitalisation pour amœbose est de 7,5 patients par an (de 1 à 18) (CHPf, données non publiées).

Les helminthiases intestinales sont également rencontrées mais il existe peu de données épidémiologiques sur ces parasitoses liées au péril fécal. Il est probable que la distribution à grande échelle d’albendazole liée au programme de lutte contre la filariose lymphatique ait contribué à diminuer la prévalence de celles-ci sans toutefois les éradiquer totalement. En effet, l’anguillulose, l’ascaridiase ou l’oxyurose sont encore diagnostiquées en Pf. De la même façon, la toxocarose et la *larva migrans* cutanée (Fig. 22) sont présentes, liées à l’importance des chiens errants. La Polynésie française, grâce au Bureau de veille sanitaire, a transmis ses données *via* le formulaire GNARF *(Global Neglected*

## Pathologies tropicales non infectieuses

### Rhumatisme articulaire aigu (rūmati tu’i māfatu)

**Jean-Marc Ségalin, Rainui Richaud, Erwan Oehler**

Le rhumatisme articulaire aigu (RAA) est une pathologie inflammatoire à médiation immune liée à une réponse inadaptée de l’organisme 2 à 4 semaines après une infection par un streptocoque du groupe A (SGA). Son incidence a considérablement diminué depuis les années 1940 dans les pays qui ont amélioré de façon significative leurs conditions sanitaires. Elle reste cependant un problème de santé publique majeur dans les pays à revenu faible ou intermédiaire, notamment en Afrique subsaharienne, en Asie du Sud et en Océanie, y compris en Pf [88]. Il est plus fréquent chez les enfants que chez les adultes. Pendant longtemps, on a considéré que le RAA succédait à une infection d’origine ORL à SGA. Désormais, il faut considérer que le RAA est aussi une conséquence des infections cutanées streptococciques. Les symptômes les plus fréquents sont la cardiopathie et l’arthrite mais des manifestations neurologiques (chorée) et dermatologiques (nodules sous-cutanés, érythème marginé) peuvent également être observées [85]. La cardiopathie rhumatismale est la principale complication du RAA. Elle constitue une maladie chronique dont la gravité et le coût justifient les programmes de contrôle de cette pathologie. En effet, l’atteinte valvulaire, lorsqu’elle évolue défavorablement, menace la fonction cardiaque et peut nécessiter une prise en charge chirurgicale.

Une cardite est qualifiée de légère lorsque l’insuffisance mitrale (IM) ou l’insuffisance aortique (IAo) n’ont ni signes cliniques évidents d’insuffisance cardiaque ni dilatation évidente des cavités cardiaques en échographie. Une cardite est qualifiée de modérée lorsque l’on observe cliniquement une cardiomégalie ou une insuffisance cardiaque légère ou en échographie une IM et IAo légères, ou une valvulopathie pulmonaire ou tricuspidienne associée à une valvulopathie gauche. Une cardite est qualifiée de grave lorsque la valvulopathie est cliniquement et/ ou échographiquement importante (insuffisance cardiaque modérée ou grave) avec une chirurgie valvulaire préalable ou imminente [46].

En Pf, le Centre du rhumatisme articulaire aigu de la DS a réuni des cardiologues, des infectiologues et des médecins généralistes pour développer des stratégies de prévention et de contrôle, d’abord au sein du ministère de la Santé de 1983 à 1999, puis au CHPf [160]. Il a été rétabli en 2019 au sein du Centre de consultation de la DS et est désormais chargé de coordonner la lutte contre le RAA, la cardiopathie rhumatismale chronique (CRC) et le dépistage scolaire.

Le RAA est une maladie à déclaration obligatoire en Pf et les données de déclaration font l’objet d’un registre nominatif. Les patients atteints de RAA/CRC sont pris en charge au titre de « longue maladie » par la CPS. Les deux principales sources de données épidémiologiques sont le Centre du RAA et la CPS. En 2021, 3 804 personnes étaient atteintes de RAA ou CRC en Pf, soit un taux de prévalence de 1 350/100 000 habitants. La même année, 249 nouveaux cas étaient déclarés à la CPS, soit un taux d’incidence annuelle de 90/100 000. Selon les critères de la *World Heart Federation,* un pays de haute endémie pour le RAA ou CRC est défini par une incidence annuelle de RAA chez les 5-14 ans supérieure à 30/100 000 ou une prévalence de CRC tous âges confondus supérieure à 200/100 000. Le Centre du RAA de Pf évalue l’incidence du RAA ou CRC chez les 5-4 ans à 309/100 000 et le taux de prévalence des CRC à 1 210/100 00. Par ailleurs, conformément aux recommandations du Programme de lutte contre le RAA 20192023, un dépistage systématique des CRC par échoscopie est organisé en Pf. La cible est la population des élèves scolarisés en sixième âgés de 12 ans en moyenne. En 2020-2023, dans cette population, la prévalence des CRC était évaluée à 2 000/100 000 selon les archipels. Parmi les 174 cas confirmés sur 3 511 enfants dépistés, 5 étaient classés « cardite grave » (Centre du RAA, données non publiées).

Les principaux facteurs de risque étant connus, les médecins doivent se méfier du RAA particulièrement chez les patients d’origine polynésienne ou mélanésienne, ceux ayant des antécédents personnels ou familiaux de RAA, de précarité sociale ou de logement surpeuplé [160]. La prévention du RAA repose sur une injection intramusculaire de pénicilline G toutes les 3 à 4 semaines avec une surveillance clinique et échocardiographique ainsi que la vérification régulière de l’état dentaire afin de prévenir le risque d’endocardite [198]. Le test de détection rapide du streptocoque du groupe A n’est pas encore utilisé en Pf mais une étude médico-économique sur son utilisation en Pf est en cours.

## Ciguatéra (ma’i ta’ero i’a)

**Clémence Gatti Howell, Erwan Oehler**

La ciguatéra est un ichtyosarcotoxisme lié à la consommation d’organismes marins tropicaux contaminés par des neurotoxines (ciguatoxines, CTXs) produites par des microalgues appartenant aux genres *Gambierdiscus* et *Fukuyoa.* Ces microalgues se développent préférentiellement en milieu tropical, au sein de gazons algaux qui prospèrent sur des substrats coralliens dégradés sous l’influence de facteurs de stress naturels (cyclones, augmentation brutale/prolongée de la température de l’eau, tsunami…) et/ou anthropiques (travaux de construction, dynamitage de passes^4^…) [199]. Au moins 7 des 19 espèces de *Gambierdiscus* connues à ce jour sont présentes en Pf dont *G. polynesiensis,* la plus toxique de toutes (Fig. 23 et Fig. 24) [90,173]. Contrairement à d’autres microalgues toxiques dont les efflorescences sont reconnaissables par une coloration de l’eau ou une mortalité massive de poissons, les efflorescences de *Gambierdiscus* sont invisibles à l’œil nu et aucun signe extérieur ne permet de distinguer un organisme marin contaminé d’un organisme sain.

Plus de 425 espèces de poissons impliquées dans des cas de ciguatéra ont été décrites dans la littérature [191]. En Pf, les *Serranidae* (mérous, loches), *Lutjanidae* (lutjans, perches, vivaneaux), *Lethrinidae* (becs de cane), *Balistidae* (balistes), *Scaridae* (poissons-perroquets), *Acanthuridae* (poissons-chirurgiens, nasons), *Sphyraenidae* (barracudas), *Carangidae* (carangues) et *Labridae* (labres, napoléons) font partie des familles de poissons les plus souvent impliquées (Fig. 25). Les espèces peuvent cependant varier d’un archipel à l’autre, voire d’une île à l’autre, en raison des variations de biodisponibilité des individus dans les lagons, des préférences alimentaires des populations ainsi que des stratégies d’évitement qu’elles ont mises en place.

Alors que les poissons lagonaires ont longtemps été considérés comme seuls vecteurs de ciguatéra, des études récentes réalisées en Pf ont montré que certains invertébrés marins tels que les bénitiers (*Tridacna maxima*), les oursins (*Tripneustes gratilla*), les gastéropodes (*Tectus niloticus*) et certains poissons des profondeurs appelés « *Paru* » étaient également susceptibles d’accumuler des CTXs à des concentrations élevées (Fig. 26) [71,72,73,208].

Les CTXs pénètrent dans la chaîne alimentaire par broutage, filtration ou prédation. Les humains se contaminent par consommation de poissons ou d’invertébrés marins, quelle que soit sa place dans la chaîne trophique (Fig. 27). De fait, tout organisme marin ayant été exposé à des efflorescences de la microalgue toxique ou ayant ingéré d’autres organismes eux-mêmes contaminés par des CTXs, doit être considéré comme un vecteur potentiel de la ciguatéra. Signalons qu’en raison de leur très grande stabilité, les CTXs demeurent actives même après congélation, cuisson, salage ou fumage des produits marins.

**Figure 23:** *Gambierdiscus polynesiensis au microscope optique / Gambierdiscus polynesiensis* under optical microscope *(© Institut Louis Malardé)* 
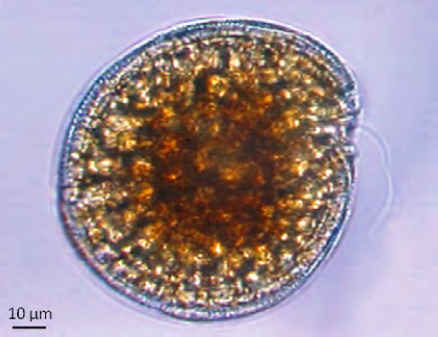

**Figure 24:** Efflorescence de cellules de *Gambierdiscus* fixées à une microalgue-support prélevée dans le lagon de Tahiti */ Efflorescence of* Gambierdiscus *cells attached to a support macroalgae taken from the lagoon of Tahiti* (© Institut Louis Malardé) 
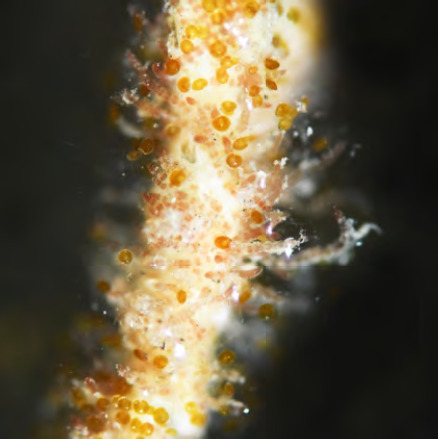

**Figure 25:** Familles de poissons impliquées dans des cas de ciguatéra en Polynésie française. A) Serranidae, B) Lutjanidae, C) Lethrinidae, D) Balistidae, E) Scaridae, F) Acanthuridae, G) sphyraenidae, H) Carangidae et I) Labridae / *Fish families involved in cases of ciguatera in French Polynesia. A) Serranidae, B) Lutjanidae, C) Lethrinidae, D) Balistidae, E) Scaridae, F) Acanthuridae, G) sphyraenidae, H) Carangidae and I) Labridae (© Randall)* 
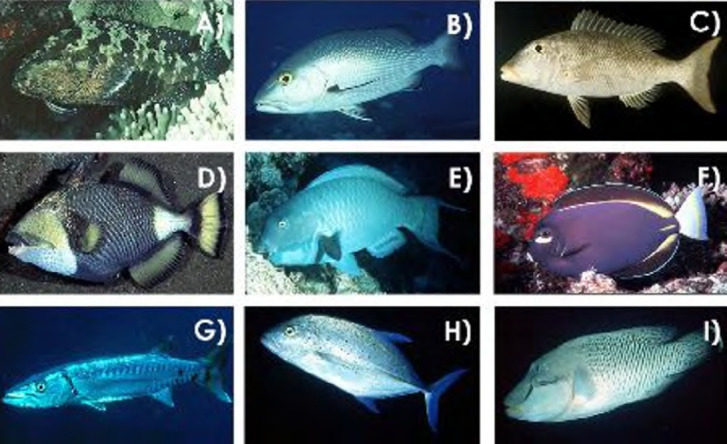

Source: Randall J.E. https://fishbase.se

**Figure 26:** Autres vecteurs possibles de la ciguatéra en Polynésie française A) Bénitier, Tridacna maxima B) Troca, Tectus niloticus C) Oursin, Tripneustes gratilla D) Poisson des profondeur de la famille des « Paru », Eumegistus illustris / *Other possible vectors of ciguatera in French Polynesia; A) Giant clam, Tridacna maxi, B) Trochus*, *Tectus niloticus C) Sea urchin*, *Tripneustes gratilla D) Deep-sea fish of the “Paru” family, Eumegistus illustris (A,B,C : © Institut Louis Malardé D : © Randall)* 
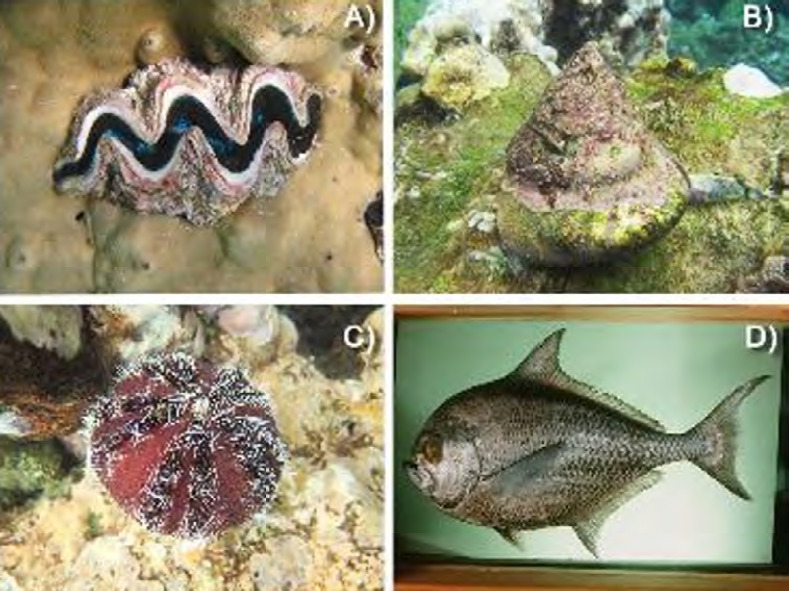

Source: Institut Louis Malardé, Randall J.E. https://fishbase.se

**Figure 27:** A) Biogénèse de la ciguatéra, B) Transfert des ciguatoxines dans la chaîne trophique, C) et D) Impacts des CTXs sur l’organisme humain (réalisation : C. Gatti) / A) Biogenesis of ciguatera, B) Transfer of ciguatoxins in the trophic chain, C) and D) Impacts of CTXs on the human organism (production: C. Gatti) (© Institut Louis Malardé) 
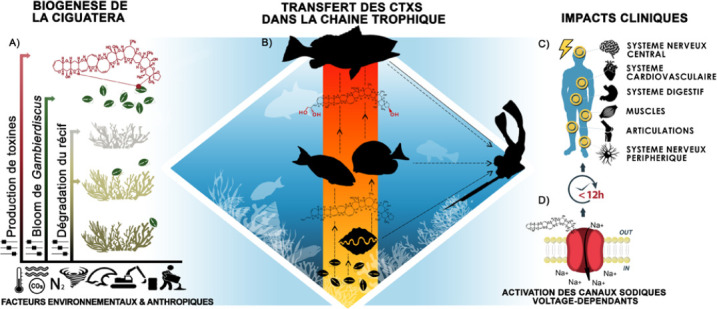

Les CTXs présentes dans les poissons carnivores diffèrent de celles retrouvées chez les herbivores, en raison de biotransformations successives qu’elles subissent au fur et à mesure de leur transfert dans les étages trophiques supérieurs [122]. Si ces transformations les rendent en général plus virulentes, cela ne signifie pas nécessairement que les intoxications par poissons carnivores sont systématiquement plus sévères que celles associées à des herbivores ou invertébrés. La quantité totale de toxines ingérée ainsi que l’état de santé initial du consommateur jouent un rôle primordial dans la nature et la gravité du tableau clinique. En cas de doute sur la potentielle toxicité d’un poisson, il est par ailleurs recommandé d’éviter de consommer la tête, les viscères et les œufs, potentiellement plus concentrés en toxines que la chair. Une hypothèse communément acceptée fait état que les gros poissons seraient plus toxiques que les petits, mais des études réalisées notamment en Pf ont mis en évidence une absence de corrélation entre la toxicité et la taille des spécimens [96].

Une fois ingérées, les CTXs passent rapidement dans la circulation sanguine pour ensuite être distribuées dans l’organisme où elles vont se fixer sur leurs principales cibles biologiques que sont les canaux sodiques voltage-dépendants des cellules excitables [174]. Ces derniers vont alors être maintenus dans un état d’ouverture permanente, conduisant à des décharges spontanées et anarchiques de potentiels d’action, source de perturbation du message nerveux. Du fait de la large distribution des canaux sodiques dans l’organisme, l’exposition aux CTXs s’exprime à travers une symptomatologie évocatrice d’apparition rapide combinant signes digestifs (douleurs abdominales, nausées, vomissements, diarrhées) parfois associés à des signes cardiovasculaires (bradycardie, hypotension), potentiellement responsables d’une déshydratation et/ou de « malaises ». Le tableau clinique est rapidement complété par un prurit féroce (d’où le nom de « gratte » parfois donné à la ciguatéra) et des troubles neurologiques (paresthésies des extrémités et/ou de la région péribuccale, dysesthésies ou allodynie au froid quasi pathognomonique) et des douleurs musculaires/articulaires. Les formes cliniques de l’intoxication semblent varier d’une région du monde à l’autre, les manifestations neurologiques étant plus prépondérantes dans le Pacifique (Fig. 28) [90].

Sur la base des données non publiées collectées par le réseau de surveillance épidémiologique de la ciguatéra entre 2018 et 2021 (n=1 004 cas), les troubles neurologiques prédominaient devant les troubles digestifs, cardiovasculaires et autres symptômes : paresthésies (80 %), diarrhées (75 %), allodynie au froid (64 %), myalgies (55 %), arthralgies (53 %), céphalées (50 %), dysesthésies buccales (49 %), prurit (47 %), asthénie (42 %), dysgueusie (33 %), hypothermie modérée (28 %), hypotension (27 %), bradycardie (22 %), dysesthésies périnéales (18 %), douleurs orofaciales (11 %), hallucinations (4 %). Si les troubles digestifs et cardiovasculaires disparaissent généralement en quelques jours, spontanément ou sous traitement symptomatique, les signes neuropsychologiques (asthénie, paresthésies, dysesthésies, prurit, difficultés de concentration, anxiété, dépression), peuvent parfois persister pendant plusieurs mois voire années [94]. On estime ainsi à plus de 20 % la proportion de patients présentant des troubles durant plus de trois mois [20]. Ces symptômes s’expriment de manière continue ou sous forme de récidives déclenchées par divers facteurs (consommation de produits marins, alcool, fruits à coque, protéines animales/végétales, en réponse à un stress intense ou à la suite d’une activité physique soutenue). Une étude exploratoire menée auprès de 49 patients atteints de ciguatéra hospitalisés au CHPf a permis d’identifier des facteurs de prédisposition potentiels aux formes persistantes dont l’âge et le tabagisme [101]. Les cas mortels liés à la ciguatéra restent anecdotiques, inférieurs à 0,1 % dans la littérature, et aucun cas n’a été signalé au cours des 24 dernières années en Pf.

**Figure 28:** Liste non exhaustive de symptômes pouvant se manifester dans le cadre d’une ciguatéra (réalisation : C. Gatti)/ *Non-exhaustive list of symptoms that can appear in the context of ciguatéra (production: C. Gatti) (© Institut Louis Malardé)* 
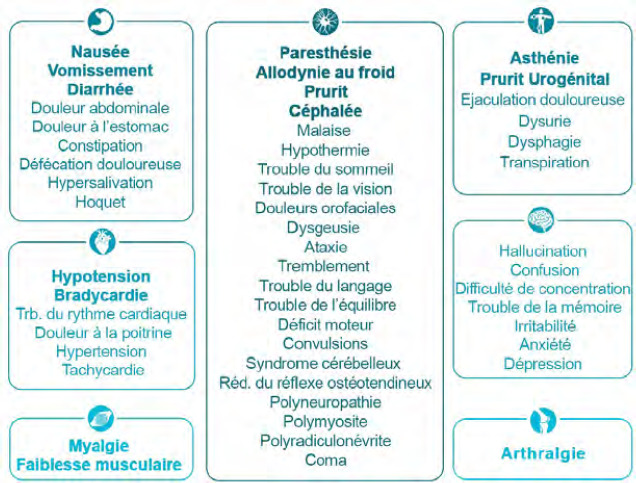

La ciguatéra est une affection non immunisante; parmi les cas incidents recensés annuellement, près de la moitié présente un antécédent d’intoxication (jusqu’à 20 chez certains individus), une situation caractéristique des populations fortement dépendantes des ressources halieutiques. Les hommes dans la quarantaine sont généralement les plus touchés. Parmi les explications à ce phénomène, leur rôle de « testeur » avant que le mets soit distribué au reste du foyer familial, et leur consommation de portions plus importantes ou de morceaux différents comme la tête et les viscères qui sont plus concentrés en CTXs [184].

Les formes pédiatriques (≤ 15 ans) peuvent représenter jusqu’à 8 % des cas signalés chaque année en Pf. Des cas de ciguatéra chez les nourrissons de moins d’un an ont déjà été relevés, le poisson lagonaire étant introduit dans l’alimentation des bébés dès le début de la diversification alimentaire. Des cas rapportés de troubles du comportement chez les nourrissons de mères allaitantes suggèrent également la transmission des CTXs par l’allaitement d’où la recommandation de suspendre l’allaitement pendant une période d’un mois par mesure de précaution [90].

Il n’existe, à ce jour, aucun test biologique ou marqueur spécifique permettant de confirmer le diagnostic de ciguatéra chez un patient. Le diagnostic repose donc sur l’anamnèse d’une consommation récente d’espèce à risque associée à une symptomatologie évocatrice. Seule la recherche de CTXs dans les restes de repas par des tests sur animaux, des tests cellulaires ou immunologiques plus sensibles et spécifiques permet de confirmer l’exposition des patients à ces toxines [184].

La plupart des cas recensés en Pf sont traités en ambulatoire ou par automédication. Peu de patients nécessitent une hospitalisation : une trentaine de cas annuels ont en moyenne été enregistrés au CHPf sur les dix dernières années. La prise en charge médicale des patients est essentiellement symptomatique [94]. L’administration de mannitol dans les cas présentant des troubles neurologiques sévères est parfois réalisée en l’absence de contre-indication, sans preuve formelle de son efficacité [165]. Un régime alimentaire spécifique d’éviction doit être suivi durant plusieurs semaines afin de limiter la réactivation/aggravation transitoire (de quelques heures à quelques jours) de symptômes tels que démangeaisons, paresthésies, dysesthésies, céphalées, douleurs musculaires et malaise général. Ce régime doit être adapté à chaque patient car le niveau de réactivité vis-à-vis de certains produits alimentaires varie considérablement d’une personne à l’autre. Il est ainsi classiquement recommandé aux patients de supprimer de leur alimentation pendant un mois ou jusqu’à disparition des symptômes, tout produit marin, fruit à coque, viande rouge et alcool, qui sont les produits auxquels la majorité d’entre eux sont réactifs. Dans les formes les plus extrêmes, certains patients vont développer une réaction aux produits dérivés de poissons et d’algues (compléments alimentaires à base d’oméga-3 issus d’huile de poisson, de spiruline…), à toute forme de protéines mêmes végétales, aux plats gras, au boissons énergisantes, café, chocolat.

Si les mécanismes biologiques sous-jacents de ce phénomène restent incompris, ce dernier finit généralement par s’atténuer spontanément, jusqu’à disparaître complètement.

La population polynésienne, très attachée à la médecine traditionnelle, fait usage de plusieurs plantes pour traiter la ciguatéra. Certaines d’entre elles ont fait l’objet d’études notamment le faux-tabac *(Heliotropium foertherianum, tahinu* en tahitien) qui est certainement la plante la plus couramment utilisée en Pf mais également celle ayant fait l’objet du plus grand nombre de travaux de recherche [134]. Ses feuilles, utilisées en décoction, contiennent un principe actif, l’acide rosmarinique, qui a montré *in vitro* une action de compétition vis-à-vis des CTXs [207]. Bien que des études complémentaires doivent être réalisées afin d’évaluer son efficacité *in vivo,* son utilisation est communément recommandée en raison du faible risque de toxicité.

Actuellement, il existe peu de textes, qui plus est obsolètes, règlementant la vente des produits de la mer potentiellement toxiques. Ces textes sont en effet difficilement applicables compte tenu de l’impossibilité d’éditer une liste définie d’espèces à proscrire et de l’absence de seuil de salubrité règlementaire. Pourtant, la salubrité des produits marins vis-à-vis du risque de ciguatéra est en passe de devenir un enjeu de santé publique et un défi économique majeur pour la Pf, raison pour laquelle plusieurs initiatives sont actuellement en cours sur le territoire afin de limiter son impact sur la population. En 2007, un réseau de surveillance épidémiologique dédié géré conjointement par l’Institut Louis Malardé et le Bureau de veille sanitaire a été créé. Ce réseau collige les signalements volontaires de cas par le personnel de santé affecté aux établissements de soins disséminés sur l’ensemble du territoire. Pour chaque patient déclaré, un formulaire standardisé est rempli par le personnel médical; les informations recueillies concernent l’île de résidence, l’âge, le sexe, les symptômes, les détails sur le repas toxique (espèces marines impliquées, partie(s) consommée(s) et zone de pêche), le nombre d’intoxications antérieures par la ciguatéra, le nombre de convives affectés. Les données sont centralisées à l’ILM qui publie un rapport annuel largement diffusé *via* le réseau de la Direction de la Santé. Depuis 2022, les statistiques de la ciguatéra sont consultables et téléchargeables en libre accès sur une plateforme dédiée (https://ciguawatch-app.ilm.pf) qui héberge également un système de déclaration en ligne ouvert aux professionnels de la santé et au grand public (Fig. 29). Dès qu’un cas est signalé, les informations sur le lieu de pêche et l’espèce impliquée sont automatiquement répertoriées sur une cartographie dynamique accessible à tous. Un grand nombre d’intoxications signalées (30 % d’après les données collectées en 2022) était associé à la consommation de poissons au restaurant ou de poissons achetés en bord de route ou dans le commerce. Par ailleurs, une Cellule de veille et de gestion du risque ciguatérique a été créée en 2021 [99]. Au sein de celle-ci, l’ILM, le Bureau de veille sanitaire, le Centre de santé environnementale et la Direction des ressources marines de Polynésie française, travaillent conjointement dans l’objectif de limiter les risques d’intoxication chez le consommateur. Dans ce cadre, chaque signalement impliquant un poisson acheté en bord de route, en magasin, au marché, ou consommé dans un restaurant, fait l’objet d’une enquête afin d’identifier la filière dont il est issu et d’informer le fournisseur du risque toxique lié au lot commercialisé. Le cas échéant, les poissons issus du même lot sont retirés des étals publics, afin d’éviter d’autres intoxications.

**Figure 29:** A) Formulaire de déclaration, B) Cartographie des zones à risque et C) Statistiques des cas de ciguatéra signalés en Polynésie française / *A) Déclaration form, B) Mapping of risk areas and C) Statistics of ciguatera cases reported in French Polynesia (© Institut Louis Malardé)* Source : plateforme CIGUAWATCH 
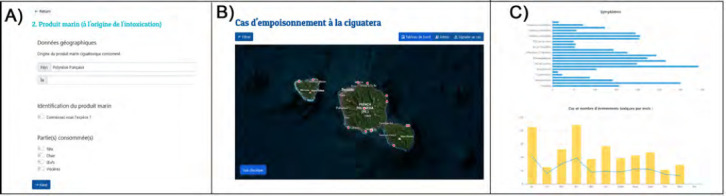

Les données recueillies grâce au réseau de surveillance indiquent un taux d’incidence annuel moyen de 158/100 000 au cours de la dernière décennie pouvant atteindre dans certaines îles plus de 18 000/100 000 habitants (Rapa, Australes) [60]. Si la tendance est à la stabilisation à l’échelle du territoire, d’importantes variations peuvent être observées dans le temps et l’espace. Il est à noter qu’en Pf comme dans la grande majorité des pays endémiques, ces statistiques souffrent d’une sous-déclaration majeure liée au fait que la déclaration n’y est pas obligatoire et, qu’en l’absence de solution thérapeutique efficace, une part importante des malades ne consulte pas. Malgré tout, la Pf reste en tête des territoires qui comptabilisent le plus de cas répertoriés à l’échelle mondiale [99].

Pour répondre à la demande croissante d’informations, un site internet d’informations générales (www.ciguatera.pf), disponible en français et en anglais, a été mis en ligne en 2015 à l’attention du grand public, et des kits d’information à l’attention des médecins et des patients ont été réalisés en collaboration avec les autorités de santé et rendus disponibles en téléchargement sur le site.

La ciguatéra représente enfin un coût non négligeable pour la collectivité, lié à la prise en charge des patients (traitements, hospitalisations, arrêts de travail…) et à la mise en place de campagnes de surveillance et de gestion du risque toxique [163]. Elle constitue par ailleurs un obstacle significatif à l’exploitation durable des ressources lagonaires, en raison du ralentissement, voire de la disparition de certaines filières de vente, y compris l’export à l’international. De plus, dans certains atolls à forte vocation touristique, l’incidence élevée des cas de ciguatéra peut, à terme, avoir un retentissement non négligeable sur les activités touristiques/récréatives et l’économie bleue dans sa globalité [3]. Par ailleurs, l’impact de cette menace doit également être appréhendé d’un point de vue sociologique. En effet, dans les îles éloignées, la dépendance aux produits de la pêche lagonaire comme ressource primaire est souvent inversement proportionnelle à l’accessibilité à d’autres aliments, et donc quasi totale dans les îles où les liaisons maritimes ou aériennes sont limitées. Aussi, pour ces populations, devoir se priver de poisson signifie s’exposer à une carence protéique et en consommer signifie s’exposer au risque d’intoxication. Dans les zones où la ciguatéra sévit de manière permanente, cette situation peut même entraîner indirectement des phénomènes de transition alimentaire forcée et la perte de la transmission de certains savoirs traditionnels relatifs aux pratiques de la pêche lagonaire.

En dehors de la ciguatéra qui représente plus de 90 % des ichtyosarcotoxismes recensés en Pf, d’autresrisques liés à la consommation de produits marins ont été observés mais restent cependant exceptionnels : tétrodotoxisme (diodons/tétrodons, tétrodotoxines), palytoxisme (crabes, palytoxines), chélonytoxisme (tortues marines, cyanobactéries) [95,100,107]. Enfin, compte tenu de la vulnérabilité de son écosystème marin, la Pf a depuis peu élargi son spectre de surveillance dans le but de se préparer à l’apparition de nouveaux risques phycotoxiniques émergeants (Ostreopsis, Azadinium…), comme observé dans d’autres régions du monde.

## Agressions par la faune

**Erwan Oehler**

### La faune terrestre

La diversité de la faune terrestre de la Pf est faible en raison de son éloignement de tout continent, de l’origine volcanique sous-marine des îles et d’une restriction drastique concernant l’introduction d’espèces^10^.

Le principal risque de morsure par des arthropodes est lié aux scolopendres. Le « cent-pieds » ou « *veri* » *(Scolopendra morsitans)* se trouve dans l’herbe, sous les pierres ou les tas de bois extérieurs, parfois dans les maisons. Elles peuvent être responsables de morsures extrêmement douloureuses qui nécessitent parfois des analgésiques dont la morphine ou une analgésie locale (Fig. 30) [91].

10. Arrêté n° 740 CM du 12 juillet 1996 fixant la liste des organismes nuisibles, des végétaux et produits végétaux susceptibles de véhiculer des organismes nuisibles dont l’importation en Polynésie française est interdite ou autorisée sous certaines conditions - http://lexp ol.cloud.pf/LexpolAfficheTexte.php?texte=155827

Seule une espèce d’araignée venimeuse, de la famille des « veuves » *(Latrodectus geometricus,* Fig. 31) [150], et deux scorpions *(Liocheles australasiae* et *Isometrus maculatus)* sont présents en Pf (Fig. 32 et Fig. 33) [229] mais, en raison de leur nature craintive et de leurs habitudes de vie, les morsures ou piqûres sont très rares et aucun cas mortel n’a jusqu’à présent été signalé. Il n’y a pas de serpents en Pf.

**Figure 30:** *Scolopendra morsistans* / Scolopendra morsitans (© E. Oehler) 
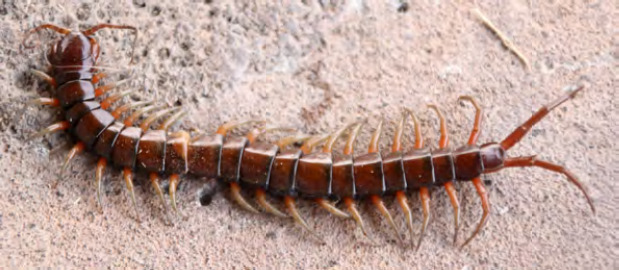

**Figure 31:** *Latrodectus geometricus* / Latrodectus geometricus (© Bernard Dupont, CC BY 2.0) Source: https://fr.wikipedia.Org/wiki/Latrodectus_geometricus#/media/Fichier:Geometric_Button_Spider_(Latrodectus_geometricus)_(6857121274).jpg 
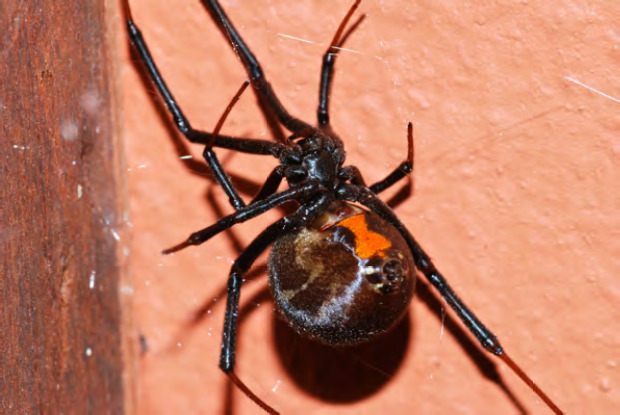

**Figure 32:** *Liocheles australasiae* / Liocheles australasiae (© OpenCage, CC BY-SA 2.5) Source: https://commons.wikimedia.org/wiki/File:Liocheles_australasiae1.jpg 
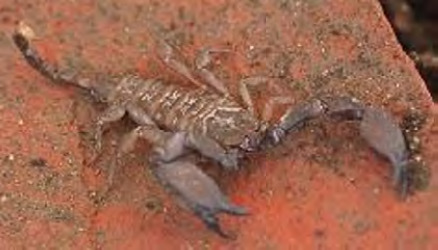

**Figure 33:** *Isometrus maculatus* / Isometrus maculatus (© Edward Bell, CC BY 2.5) Source: https://commons.wikimedia.org/wiki/File:Isometrus_maculatus_56192878.jpg 
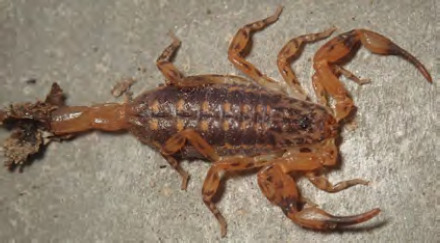

En pratique, ce sont les morsures de chiens domestiques ou errants qui causent le plus de blessures. Il faut cependant noter que la Pf est exempte de la rage grâce à une politique sanitaire vétérinaire stricte appliquée aux animaux avant leur entrée en Pf et de l’absence de chauves-souris^11,12^.

### La faune marine

Les principaux risques, mais relativement rares, dus à la faune marine en Pf sont les morsures de requins et de murènes. Cent-vingt-cinq cas de morsures de requins ont été recensés depuis 1940, la plupart concernant essentiellement l’activité de pêche sous-marine au harpon ou des plongeurs nourrissant les prédateurs. Quatre de ces morsures ont été marquées par le décès de la personne [64]. Un travail sur l’accidentologie des attaques de requins en Pf de 1980 à 2015 recensait 92 attaques représentant une fréquence élevée rapportée au kilomètre côtier, expliquée par un nombre important d’interactions humains/requins et des facteurs de risque comportementaux. Les attaques recensées concernaient en majorité les pêcheurs sous-marins et étaient dans la plupart des cas de faible gravité en lien avec l’implication de requins de petites et moyennes tailles (requins à pointes-noires *Carcharhinus melanopterus-*et requins gris du récif *Carcharhinus amblyrhynchos),* alors que les requins de plus grande taille étaient moins souvent impliqués (requins citron *Negaprion brevirostris,* requins tigre *Galeocerdo cuvier* ou requins à pointes blanches du large *Carcharhinus albimarginatus).* Il est à noter que le grand requin blanc *(Carcharodon carcharias)* et le requin bouledogue *(Carcharhinus leucas)* ne sont pas observés près des côtes polynésiennes (Fig. 34) [76]. Les murènes *(Gymnothorax javanicus*) attaquent généralement en réponse à une agression directe présumée ou en confondant une partie du corps humain avec un poisson. Leurs dents acérées peuvent entraîner des plaies profondes et délabrantes lorsqu’elles effectuent des mouvements de rotation sur elles-mêmes. Les raies armées comme les raies pastenagues (*Dasyatis pastinaca*) peuvent quant à elles infliger des blessures par le biais d’un dard fixé sur la queue. Outre les lésions mécaniques liées à la perforation et aux lacérations causées par les denticules présentes sur l’aiguillon, des lésions nécrotiques et une envenimation peuvent être observées en rapport avec la libération de venin de glandes situées sur les faces latérales de l’aiguillon [146].

**Figure 34:** Requin à pointes noires *Carcharhinus melanopterus* (A), requin citron *Negaprion brevirostris (B) / Blacktip shark* Carcharhinus melanopterus *(A), lemon shark* Negaprion brevirostris *(B)* (© A. Scuiller (A), E. Oehler (B)) 
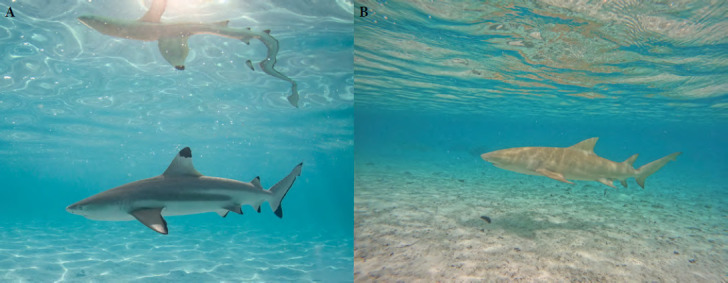

Parmi les autres risques liés à la faune marine, la Pf héberge l’étoile de mer *Acanthaster planci (taramea)* dont la piqûre peut provoquer des nécroses cutanées ainsi que des manifestations systémiques (nausées, vomissements, céphalées et douleurs articulaires jusqu’à un choc anaphylactique ou une paralysie) en lien avec la production de multiples toxines (Fig. 35) [117]. Les *Scorpaenidae (nohu)* qui regroupent le poisson-pierre (*Synanceia* sp), le poisson-scorpion (*Scorpaenopsis* sp) et la rascasse volante *(Pterois* sp) sont des poissons carnivores équipés de dards venimeux (Fig. 36). Rapidement après la piqûre, survient une intense douleur qui peut provoquer un malaise et une noyade si le traumatisé est toujours dans l’eau. Selon l’intensité de l’envenimation qui dépend du nombre d’épines impliquées et de la profondeur des lésions, on peut observer différents signes locaux (douleur, œdème, nécrose) et généraux (fièvre, céphalées, nausées, vomissements, paralysie musculaire, œdème pulmonaire, hypotension, troubles du rythme voire arrêt cardiaque). La prise en charge immédiate comprend l’immersion dans de l’eau chaude pour détruire le venin thermolabile et une antalgie ainsi qu’une antibiothérapie et/ ou une prise en charge chirurgicale en cas de plaie profonde ou nécrotique. Un antivenin produit en Australie peut être utilisé dans les envenimations les plus graves [210].

**Figure 35:** *Acantaster planci* / Acantaster planci (© E. Oehler) 
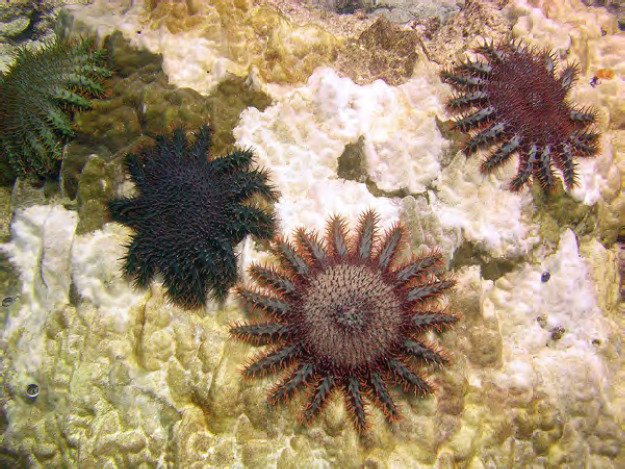

**Figure 36:** *Scorpaenopsis venosa* / Scorpaenopsis venosa (© E. Oehler) 
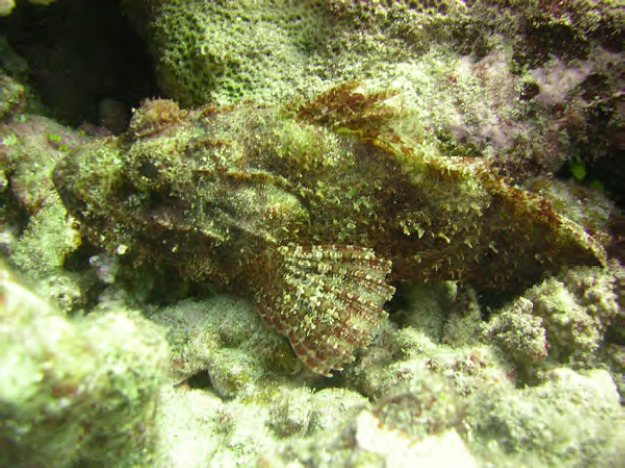

Les cônes (genre *Conus)* sont des coquillages qui utilisent un harpon pour injecter un puissant venin neurotoxique permettant de paralyser leurs proies. Les piqûres humaines sont dans la plupart des cas le résultat de manipulations intempestives. Elles peuvent entraîner une douleur insupportable et, à l’endroit de la piqûre, un engourdissement, une ischémie et une nécrose qui peut s’étendre. Une paralysie musculaire généralisée, une insuffisance respiratoire, un collapsus cardiovasculaire, un coma et la mort peuvent survenir si le patient n’est pas traité rapidement. La prise en charge est essentiellement symptomatique avec, au stade initial, la mise en place d’une bande élastique modérément serrée de l’ensemble du membre piqué pour diminuer la rapidité de diffusion des toxines avant la prise en charge hospitalière [130,131].

Divers autres organismes peuvent être sources de problèmes pour le baigneur tels que les anémones de mer, les méduses et coraux de feu qui peuvent être responsables de lésions de type urticariennes ou des brûlures, ainsi que les oursins et coraux qui peuvent être sources de corps étrangers et blessures diverses. Des cas de dermatites des baigneurs ou dermatites des surfeurs sont également rapportés pendant la saison chaude, causées par des larves de méduses ou d’anémones, et qui entraînent des lésions prurigineuses diffuses, notamment sous les maillots de bain et tee-shirts en lycra. Un séchage soigneux de la peau permet d’éviter ou de limiter les irritations [83,218].

Bien qu’exceptionnelle en Polynésie, la présence du serpent marin *Hydrophis platurus,* seule espèce d’Elapidae marin pélagique, a été décrite par Linné en 1766 et quelques spécimens ont été collectés dans l’archipel de la Société [124]. Leur morsure peut provoquer une envenimation curarisante (type cobra) grâce à un venin composé de neurotoxines se fixant sur les récepteurs neuromusculaires à acétylcholine. Une hypersensibilité, des paresthésies et une paralysie musculaire du membre touché voire une paralyse respiratoire peuvent compliquer la morsure [44].

## Pathologies cardiovasculaires et métaboliques

L’espérance de vie en Pf, inférieure de quelques années à celle de l’Hexagone, s’explique par la forte prévalence des maladies non transmissibles, prévalence en augmentation par rapport aux chiffres déjà élevés de 2010 [25]. Entre 2005 et 2010, le taux standardisé de mortalité par maladies cardiovasculaires en Pf était très significativement supérieur à celui de France métropolitaine et des DOM, notamment chez les femmes pour lesquelles il était 2,5 fois supérieur (1,9 fois chez les hommes) [240]. En 2017, les maladies cardiovasculaires étaient responsables de 27 % des décès : 29 % étaient dus aux cardiopathies ischémiques (infarctus du myocarde et cardiopathies ischémiques chroniques), 26 % aux autres formes de cardiopathies (essentiellement l’insuffisance cardiaque), 24 % aux maladies cérébrovasculaires (accidents vasculaires cérébraux, hémorragiques ou par infarctus), 13 % aux maladies hypertensives et 6 % aux maladies des artères, artérioles et capillaires. Elles étaient la première cause de décès chez les hommes (et première cause de mortalité prématurée) et la deuxième cause chez les femmes après les cancers [239].

## Diabète sucré (ma’i ‘ōmaha tihota)

**Marine Jullien, Sébastien Nunez**

Dans le monde, en 2021, on ne dénombrait pas moins de 530 millions de diabétiques dont 95 % de type 2 et des estimations évaluent que ce chiffre pourrait atteindre 1,3 milliard d’individus d’ici 2050 [183]. Les nations insulaires du Pacifique présentent une prévalence de diabète de type 2 parmi les plus élevées au monde, notamment la République de Nauru où les estimations étaient de 40 % de la population dans les années 19801990 [227]. La dernière étude épidémiologique de prévalence du diabète de type 2 réalisée en Pf estimait celle-ci à 18 % en 1995 chez les plus de 16 ans et, selon l’Atlas du diabète de la Fédération internationale du diabète, la prévalence comparative des pays ajustée en fonction de l’âge l’estimait en 2021 à 25,2 %, soit la deuxième place après le Pakistan [221]. L’enquête STEPS 2019 rapportait quant à elle que 11 % de la population étudiée était suivie pour un diabète, que les deux tiers étaient traités et que la prévalence était estimée à 14 % [82]. Chaque année, 3 000 nouveaux cas sont ainsi enregistrés auprès de la CPS. Cette évolution péjorative de la prévalence est multifactorielle : changement de régime alimentaire vers une alimentation hyperglucidique/lipidique/calorique, sédentarité, progression du surpoids et de l’obésité sur un fond de transmission familiale (tant sur l’éducation hygiéno-diététique que sur la prédisposition génétique du diabète de type 2 en soit et de polymorphismes génétiques) [123]. Dans l’étude *Identification of a candidate genetic variant for the high prevalence of type II diabetes in Polynesians,* Myles *et al.* ont en effet identifié le gène candidat PPARGC1A chez les Polynésiens, impliqué dans la régulation du métabolisme glucidique, pouvant expliquer cette susceptibilité. Ces mêmes auteurs ont également évoqué une théorie selon laquelle les migrations et les périodes de famine en résultant ont exercé une pression de sélection génétique en faveur du stockage énergétique et décrite comme la « *thrifty gene hypothesis* » ou théorie du « gène économe »> [168]. L’hypothèse d’une insulinopénie relative plus marquée que chez les Européens a également été soulevée [216].

De façon générale, en plus de l’augmentation de la prévalence, on constate que l’âge au diagnostic diminue avec un diagnostic de diabète de type 2 posé avant l’âge de 40 ans, dont une partie pourrait être des diabètes MODY *(Maturity Onset Diabetes of the Youth)* dûs à l’implication de variants pathogènes d’un seul gène (le plus souvent impliqué dans la sécrétion d’insuline et/ou le développement pancréatique). Ceux-ci touchent des patients avant 40 ans avec un phénotype pouvant s’apparenter au diabète de type 2 et représenteraient 2 à 3 % de l’ensemble des diabètes [51]. Il n’existe à ce jour aucun chiffre permettant de dire si le MODY est surreprésenté en Pf. Le diabète de type 1 représente en France hexagonale 10 à 15 % de l’ensemble des patients diabétiques. La prévalence en Pf semble nettement moindre mais aucune donnée n’est disponible à ce sujet (200 patients suivis au CHPf). L’accès aux pompes à insuline est possible en Pf et le CHPf, centre initiateur, compte 51 patients diabétiques de type 1 sous pompe dans sa file active. Deux prestataires extérieurs sont présents pour assurer la livraison du matériel et le suivi obligatoire.

Le diabète gestationnel est un diabète présent uniquement durant la grossesse qui disparait par la suite. Il est cependant un facteur de risque de développer plus tard un diabète de type 2 notamment si les règles hygiéno-diététiques ne sont pas respectées et d’autant plus qu’il existe un terrain familial prédisposant. Une étude réalisée au CHPf en 2019 a montré que 2 % des grossesses étaient associées à un diabète de type 2 soit dix fois plus que ce qui est observé en France hexagonale et une prévalence de 16 % du diabète gestationnel (2,5 fois celle de l’Hexagone) contre 11,6 % en 2015. Une augmentation de celle-ci chez les femmes en âge de procréer est donc attendue en raison de la tendance à l’augmentation des cas de diabète de type 2 et d’obésité chez les jeunes. Il est cependant à noter que l’obésité constitue un facteur de risque d’infertilité qui, associée à un diabète déséquilibré (type 1, type 2 ou gestationnel) diminue les chances de conception naturelle et/ou engendre des grossesses à risque tant sur le plan maternel que fœtal : macrosomie, prééclampsie, complications mécaniques à l’accouchement, progression des complications micro-et macroangiopathiques du diabète… [52].

Une étude non publiée réalisée au CHPf en 2007 sur les données PMSI de l’année 2005 montrait un impact important du diabète sur l’ensemble des activités, tant sur les hospitalisations que sur les consultations externes, avec une part importante du diabète gestationnel. Cette même étude soulignait les difficultés de surveillance de la bonne prise en charge du diabète et mettait en évidence que près de la moitié des patients dialysés étaient diabétiques [89]. Parmi les complications du diabète, le pied diabétique qui résulte d’un processus multifactoriel (traumatisme souvent minime sur un lit de déséquilibre glycémique chronique avec une composante de neuropathie périphérique et/ ou de macroangiopathie et/ou d’infection) est couramment rencontré en Pf du fait d’un chaussage inadapté. En effet, dans un climat tropical humide, le port de chaussures ouvertes voire l’absence de chaussage constituent une « mode locale » même dans les actes du quotidien pour le ménage, les courses, le jardinage ou la conduite de deux roues.

Les traitements accessibles en Pf sont similaires à ceux que l’on trouve sur le marché métropolitain (biguanides, sulfamides, glinide, incrétines, iSLGT2 et insulines). Ils sont cependant soumis à des règles de remboursement différentes : seule la présence d’une complication liée au diabète permet au patient de bénéficier d’une prise en charge en affection de longue durée, pour une durée limitée à renouveler. La non prise en charge à 100 % peut donc représenter un frein pour les patients en situation de précarité et favoriser l’arrêt prématuré des traitements. L’observance thérapeutique semble par ailleurs aléatoire avec des raisons parfois intriquées : coût, difficultés pour le renouvellement d’ordonnance, éloignement géographique par rapport au médecin prescripteur ou à la pharmacie, opposition médecine traditionnelle et sa thérapeutique *(raau tahiti)* et médecine occidentale, fausses croyances quant à la dangerosité des médicaments.

Enfin, en termes d’offre de soins, le prorata diabétologues/patients diabétiques est en défaveur du corps médical. Six diabétologues exercent en Pf, tous à Tahiti, 3 au CHPf dans un service de 12 lits avec 2 diététiciennes, 2 infirmières d’éducation thérapeutique et 3 autres en libéral. Ces 6 médecins assurent la prise en charge de la totalité des patients de Pf (environ 45 000 personnes diabétiques) en consultation à Tahiti ou en mission de « consultation spécialisée avancée » dans certaines îles accompagnés d’une diététicienne. Ces missions durent de 3 à 10 jours, à raison de 6 missions par an aux Marquises, 4 aux Australes et 4 aux Tuamotu-Gambier assurées par les médecins du CHPf. Les diabétologues libéraux assurent quant à eux quatre missions annuelles dans chacune des îles Sous-le-Vent. Durant ces déplacements sur les îles, les médecins réalisent des consultations orientées vers la diabétologie mais s’occupent également des pathologies endocriniennes avec la possibilité de réaliser des échographies thyroïdiennes et des cytoponctions de nodules thyroïdiens grâce à un échographe portable. Dans les cas les plus complexes où tout ne peut être géré en « consultation délocalisée », une hospitalisation est programmée au CHPf. Une diététicienne accompagne le médecin durant ces missions et reçoit les patients adultes et enfants pour des consultations en lien avec le diabète, l’obésité ou la dénutrition. Ce sont ainsi en 2022 plus de 700 patients qui ont bénéficié d’une consultation d’endocrinologie lors de ces missions.

## Obésité, hypertension artérielle, tabagisme

**Erwan Oehler, Marine Jullien**

L’« épidémie » d’obésité observée depuis plusieurs années dans le monde n’épargne pas les nations insulaires du Pacifique, bien au contraire, puisque selon l’OMS, en 2016, les 10 premières nations ayant le plus important pourcentage d’obèses se situaient dans la région Pacifique [182]. La Pf n’y est pas représentée en tant que telle mais dans les chiffres de la France et pourtant, d’après l’étude STEPS 2019, 48 % des adultes étaient obèses alors que ce pourcentage était de 40 % en 2010. Le surpoids dans son ensemble passait quant à lui de 70 % en 2010 à 75 % de la population avec une forte augmentation de ces chiffres dans la tranche des 18-29 ans et avec un lien avec un bas niveau d’éducation [82]. L’étude PODIUM montrait déjà qu’entre 2007 et 2008, la prévalence de l’obésité en Pf était supérieure aux autres territoires français ultramarins (Guadeloupe, Martinique et Guyane) à la fois chez les adultes et chez les enfants chez qui la prévalence du surpoids et de l’obésité était respectivement de 17 et 16 % [67]. La prévalence de l’obésité chez les enfants était en 2016 de 25,4 % chez les filles et de 22,4 % chez les garçons [1].

Concernant l’alimentation, plusieurs problématiques sont rencontrées [77] :

- la hausse du coût des aliments liée à l’inflation (moyenne estimée à 4 % en octobre 2023 selon les chiffres de l’ISPF) dans un contexte où 20 % de la population vivait en dessous du seuil de pauvreté monétaire relative en 2015 et où le salaire minimum est inférieur de 13 % à celui de l’Hexagone. Une liste de produits dits « de première nécessité » est ainsi régulièrement dressée^5^ pour permettre aux ménages les plus en difficulté de subvenir à leurs besoins. Beaucoup des produits de cette liste sont cependant des produits transformés salés, gras, des féculents…
- des familles recomposées et élargies (plus de la moitié des Polynésiens vivent dans une famille nombreuse ou dans un ménage comprenant plusieurs familles) dans lesquelles un seul salaire subvient aux besoins de la famille;
- la présence sur le marché d’aliments ultra-transformés (« coca-colonisation ») et de produits sur lesquels les normes européennes sur les teneurs en sucre ne s’appliquent pas (les sodas d’une même marque sont par exemple plus sucrés en Pf comme dans l’ensemble des outre-mer que dans l’Hexagone) avec, depuis peu, la volonté de réduire ces écarts;
- un rapport aux repas qui est différent du modèle métropolitain : l’alimentation en Pf représenterait, selon l’anthropologue C. Serra-Mallol, un comportement hérité de « l’ancienne société » où « bien manger pour un Polynésien, c’est d’abord manger beaucoup, et manger plusieurs fois dans la journée ».

Dans ce contexte d’épidémie de diabète et d’obésité, le Conseil économique, social, environnemental et culturel de la Pf (CESEC) a édité en 2019 un rapport synthétisant l’état des lieux sur la pathologie et exposant les différents niveaux d’action engagés : mise en place de campagnes de santé ciblées (dans les écoles primaires par exemple), programmes d’éducation thérapeutique (programmes télévisés, Maita’i sport santé), campagnes de sensibilisation (journée mondiale du diabète le 14 novembre) et de centres adaptés (Soins de suite et de réadaptation Ora Ora). Ces actions sont mises en place par les pouvoirs publics et les mairies de certaines grandes communes mais ils sont encore trop peu nombreux et se heurtent à de nombreuses difficultés : non pérennisation dans le temps, absence de programmes ciblés sur certaines populations (adolescents, personnes âgées), accessibilité difficile (lieu de vie éloigné du lieu de ces actions, problème de transport), horaires non compatibles, manque d’information. En avril 2023 a été présenté le Plan de transition alimentaire de la Polynésie française qui intègre et renforce des actions et programmes portés par les différents ministères et leur mise en perspective pour les 10 prochaines années. Il comporte des mesures nouvelles permettant de répondre à l’objectif central de l’amélioration de la qualité sanitaire de l’alimentation et de son accessibilité. Ce système alimentaire a pour but de procurer à tous une alimentation saine, abordable et durable utilisant davantage de produits locaux.

Selon une étude de 2014, l’hypertension artérielle (HTA) concernait 24,5 % de la population, discrètement moins qu’aux Antilles mais plus qu’en Guyane. Cette étude montrait une connaissance et un traitement de l’HTA moindres que dans les autres DOM-TOM ainsi qu’un moins bon contrôle de cette HTA. La Pf avait le plus fort taux de prévalence de l’obésité (33,1 %) et la fraction d’hypertension attribuable à l’obésité dans la population la plus élevée (35,5 % contre 23,6 % en Guyane, 13,3 % en Martinique et 12,3 % en Guadeloupe) [9]. L’enquête STEPS 2019 montrait quant à elle une prévalence de l’HTA de 30 % de la population adulte, le principal facteur de risque étant l’obésité (OR à 2,6) et le sexe masculin (OR à 1,6). Il était à noter que 16 % et 33 % des patients entre 18 et 29 ans et 30 et 44 ans respectivement étaient hypertendus, chiffres en augmentation depuis 2010 [82].

L’enquête STEPS 2019 estimait que 37 % des adultes fumaient des produits à base de tabac, en légère diminution par rapport à 2010 où 41 % de la population était fumeur et avec une baisse plus significative chez les femmes de 4569 ans. Dans cette population, 86 % étaient des fumeurs au quotidien fumant en moyenne 11 cigarettes industrielles par jour et 81 % avaient fumé leur première cigarette entre 15 et 20 ans, représentant une augmentation significative depuis 2010 (52 % à cette époque) et indiquant une entrée dans la pratique du tabagisme à un âge plus jeune [82].

## Accidents vasculaires cérébraux

**Raphaël Buon**

La seule unité neurovasculaire (UNV) de Pf est située à Tahiti, au CHPf. L’accès aux soins en urgence dans les délais de thrombolyse (moins de 4h30) est souvent difficile, voire impossible, en fonction de l’île dans laquelle vit le patient, le temps de rapatriement vers Tahiti pouvant dépasser ce délai. Par ailleurs, même si les archipels des îles Sous-le-Vent (Raiatea) et des Marquises (Nuku-Hiva) disposent d’un scanner depuis 2019 et 2021 respectivement, il n’existe pas pour le moment de réseau de télé-AVC permettant des thrombolyses à distance. De même, la sensibilisation des populations à consulter en urgence en cas d’AVC reste insuffisante : il n’est pas rare que les patients attendent plusieurs jours (ou semaines) avant de consulter.

En 2022, 437 patients ont été hospitalisés au CHPf pour un AVC, 87 % étaient d’origine polynésienne. De manière comparable à la métropole ces AVC étaient répartis en 85 % d’infarctus, 14 % d’hématomes cérébraux et 1 % de thrombophlébites cérébrales. Cinquante-deux pourcent des patients étaient des hommes (sex-ratio à 1,1), l’âge moyen était de 63,4 ans (contre 73 ans en métropole) et plus de 26 % avaient moins de 55 ans (10 % environ en métropole). La létalité intra-hospitalière s’établissait à 12 %.

Le nombre de thrombolyses réalisées au CHPf est en constante augmentation (34 en 2019 et 67 en 2023 soit une augmentation de 49 %), à mettre en lien avec une meilleure organisation des soins et une augmentation de l’incidence totale des AVC. Le *gold standard* de la prise en charge de l’AVC en phase aiguë, la thrombectomie mécanique, est malheureusement peu accessible : en 2022-2023 le CHPf ne comptait qu’un seul neuroradiologue interventionnel formé, sans astreinte opérationnelle. Dans ce contexte, seules 16 thrombectomies ont ainsi été réalisées.

Les deux causes les plus fréquentes d’AVC ischémiques en 2022 étaient les causes cardioemboliques (fibrillation auriculaire en majeure partie) puis les causes athéromateuses, avec une forte prévalence de l’athérome intracrânien comparativement à l’athérome carotidien cervical, probablement en lien avec des facteurs métaboliques et génétiques connus dans les populations asiatiques [125].

Comparativement à la France hexagonale, les patients victimes d’AVC sont donc plus jeunes et il existe une prévalence plus élevée des facteurs de risque cardiovasculaires traditionnels. Chez les moins de 55 ans victimes d’AVC ischémique, on relevait 33 % d’obèses, 17 % de diabétiques, 37 % d’hypertendus, 12 % de dyslipidémiques, 29 % de tabagiques actifs et 7 % de consommateurs de toxiques.

Parmi les causes spécifiques d’AVC, on retient les complications multiples des patients opérés d’une cardiopathie rhumatismale et porteurs de prothèses valvulaires mécaniques, en lien avec l’inobservance et/ou les difficultés d’équilibration des traitements anticoagulants et les endocardites infectieuses.

On observe également plus fréquemment qu’en métropole des syndromes de Moyamoya unilatéraux caractérisés par une sténose occlusive de la terminaison carotidienne avec ou sans développement de collatéralités (Fig. 37). L’histoire naturelle de ces tableaux cliniques est très différente des maladies de Moyamoya classiquement décrites avec un faible taux de complications à long terme (récidive ischémique ou hémorragique, troubles cognitifs), l’absence de bilatéralisation des anomalies lors du suivi et l’absence de nécessité d’intervention neurochirurgicale [43].

Enfin, on soulignera la problématique de l’observance thérapeutique et du suivi en général des pathologies chroniques, souvent en cause dans la survenue des AVC ou des récidives.

**Figure 37:** Artériographie cérébrale d’un patient de 22 ans ayant présenté un AVC sylvien gauche. Sténose occlusive de M1 gauche proximale (flèche rouge) avec développement de collatéralités (têtes de flèche noires). Pas d’anomalies sur la circulation à droite (non montrée) */ Cerebral arteriography of a 22-year-old patient with a left sylvian stroke. Occlusive stenosis of the proximal left M1 (red arrow) with development of collaterals (black arrowheads). No anomalies on right-hand traffic (not shown)* (© *R. Buon*) 
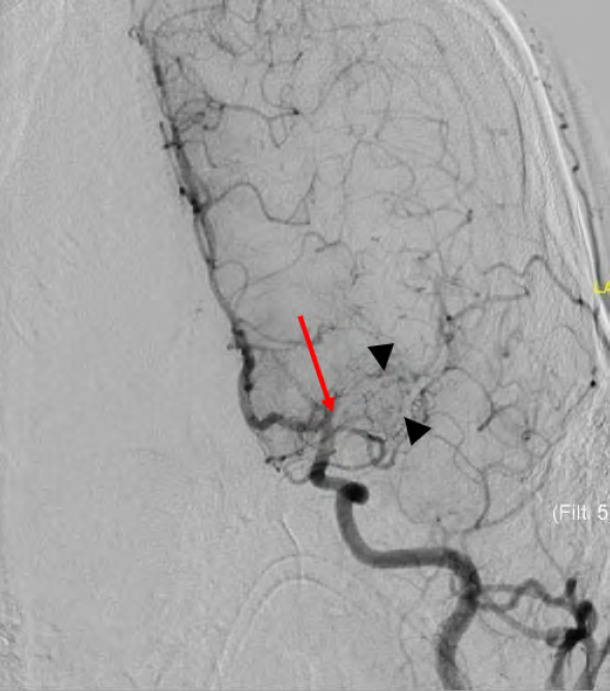

## Cardiopathies

**Rainui Richaud**

Le CHPf est le seul établissement en Pf doté d’une unité de soins intensifs cardiologiques et d’un plateau technique complet associant cardiologie interventionnelle (coronarographie et rythmologie), explorations cardiologiques, IRM et scanner cardiaques et cardiologie nucléaire. Un centre d’angioplastie coronaire a été créé en 2002 avec une équipe interventionnelle présente 24h/24 et une activité en constante augmentation (Fig. 38) [58]. Une très grande majorité des prises en charge spécialisées est donc réalisée au CHPf avec notamment en 2022 : 900 scintigraphies cardiaques, 900 échocardiographies transoesophagiennes, 800 échographies de stress, 1 500 coronarographies et 600 angioplasties, 300 actes de rythmologie interventionnelle, consultations de cardiopédiatrie clinique…

Parmi les actes de coronarographie, on estime à près d’une centaine le nombre de syndromes coronariens aigus (SCA) de type STEMI pris en charge et il y aurait 2 à 4 fois plus de SCA NSTEMI ou STEMI pris en charge hors délais (Rainui Richaud, données non publiées). Par ailleurs, si l’angioplastie reste le traitement de première intention des SCA, la thrombolyse reste malgré tout très utilisée en raison des délais de prise en charge des patients liés aux particularités géographiques de la Pf. De même, outre la clinique et l’ECG, en l’absence de laboratoire de biologie médicale sur chaque île, la stratégie diagnostique d’un SCA repose largement sur l’utilisation de bandelettes de détection rapide immunochromatographique de la troponine.

**Figure 38:** Activité de cardiologie interventionnelle (angioplasties dont urgences) et de rythmologie au CHPf (réalisation : R. Richaud, données non publiées) / *Interventional cardiology (angioplasties including emergencies) and rhythmology activity at the CHPf (production: R. Richaud, unpublished* *data)* 
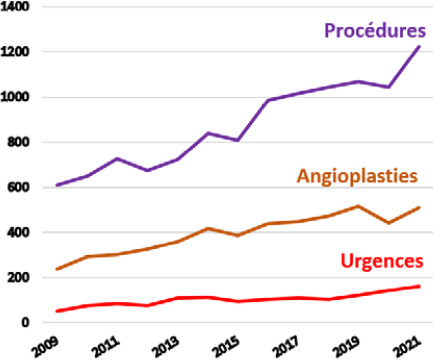

En l’absence de chirurgie cardiaque en Pf, l’activité de cardiologie interventionnelle oblige à une prudence relative dans la réalisation des angioplasties complexes et des procédures structurelles. De même, les urgences cardiochirurgicales (dissections aortiques, atteintes coronaires tritronculaires, valvulopathies et/ou endocardites instables, cardiopathies congénitales sévères, ECMO artérioveineuse…) constituent ainsi un véritable challenge humain et logistique pour une évacuation sanitaire internationale.

La forte prévalence du RAA est responsable de cardiopathies valvulaires et rythmiques nécessitant la mise sous traitement anticoagulant au long cours : on estime ainsi qu’entre 3 500 et 3 600 patients sont traités par antivitamines K et que la moitié est suivie par le système Coagucheck® *via* un financement dédié. Le service de cardiologie du CHPf assure ainsi une activité de « Clinique des anticoagulants » avec une infirmière dédiée à la réception des résultats INR réalisés par prélèvement veineux et/ou Coagucheck®. Supervisée par les praticiens hospitaliers cardiologues, cette infirmière réalise l’interface avec les patients pour l’éducation thérapeutique, le rendu des résultats, la gestion des déséquilibres des anticoagulants et la relation avec les autres services du CHPf et les professionnels libéraux. C’est également la « Clinique des anticoagulants » qui gère la distribution des appareils Coagucheck® et des consommables (bandelettes) pour les professionnels de soins et certains patients éligibles (porteurs de valves mécaniques notamment).

Enfin, grâce aux progrès des technologies connectées, on constate un essor de la télésurveillance, notamment celle des dispositifs implantables de type *pacemakers* et défibrillateurs. Les boitiers implantés télétransmettent ainsi les données de suivi et les alertes graves et/ou vitales au service de cardiologie *via* un télétransmetteur connecté au réseau téléphonique. Ce dispositif permet un allègement du suivi présentiel des patients, une économie par diminution des évasans et une réactivité en cas de dysfonctionnement du dispositif, d’arythmie ou autres évènements cliniques. Différents inconvénients demeurent cependant comme l’impossibilité de modifier les paramètres à distance rendant indispensable la présence des patients en cas de nécessité de réglage du dispositif, la nécessité de former des paramédicaux (équivalents d’infirmiers en pratique avancée) en raison de la quantité de données à traiter (tri des évènements significatifs de ceux ne nécessitant pas d’action médicale par exemple) et la nécessité d’une homogénéité dans les dispositifs pour ne pas multiplier les marques et donc les systèmes logiciels [16].

## Goutte (ma’i ‘u’u)

**Erwan Oehler**

Il est maintenant communément admis que la goutte constitue un facteur de risque cardiovasculaire indépendant et la Pf occupe la première place mondiale au classement en termes de prévalence puisqu’une enquête réalisée en 2021 a montré que 25,5 % des hommes et 3,5 % des femmes étaient touchés, soit une prévalence globale de plus de 14,5 % contre 0,9 % dans l’Hexagone [186]. Les individus jeunes n’en sont par ailleurs pas exempts puisqu’on observe une prévalence de 2,5 % chez les hommes de 18 à 29 ans et de 32 % chez les hommes de 30 à 39 ans. Dans cette même étude, l’hyperuricémie concernait 71,6 % de la population. En analyse multivariée, la goutte était par ailleurs associée au diabète type 2 (OR = 1,81), à des antécédents d’AVC (OR = 2,15) et au pourcentage de graisse viscérale (OR = 1,18). Le régime alimentaire, le syndrome métabolique et les médicaments expliqueraient cette prévalence de même que des facteurs génétiques [7,187].

Le diagnostic est établi par la ponction de l’articulation concernée ou, le plus souvent, l’amélioration rapide sous traitement probabiliste par colchicine ou anti-inflammatoires stéroïdiens ou non. Après confirmation diagnostique, la goutte est traitée selon les recommandations de la Société française de rhumatologie par un traitement de fond par hypo-uricémiant (allo-purinol ou fébuxostat). L’objectif est de diminuer l’hyperuricémie en dessous de 50 mg/l (300 μmol/l), 60 mg/l (360 μmol/l) correspondant au seuil de solubilité de l’urate monoso-dique [137]. Plusieurs études dans le monde ont montré que la prise en charge de ces patients était inadéquate ou insuffisante [149]. L’étude de Pascart *et al.* a également montré cette tendance en Pf puisqu’un tiers seulement des patients était traité par hypo-uricémiant et que moins de 5 % d’entre eux atteignait l’objectif fixé [187]. Un programme d’éducation thérapeutique des patients a été mis en place au CHPf dans le but d’améliorer la prise en charge des goutteux hospitalisés [27].

Ainsi, du fait de la fréquence des arthrites septiques et de la goutte, il n’est pas rare de voir des patients hospitalisés pour des douleurs articulaires avec le challenge diagnostic que cela implique d’autant plus que les atteintes goutteuses polyarticulaires se présentent parfois avec un syndrome fébrile et inflammatoire important (Fig. 39). De même, un patient goutteux hospitalisé pour quelque autre raison peut voir apparaître une crise marquée par l’apparition ou la réapparition d’un syndrome fébrile ou inflammatoire pouvant motiver la prescription ou la modification d’une antibiothérapie si l’hypothèse de la goutte n’est pas évoquée.

**Figure 39:** Multiples tophi plantaires et ulcération malléolaire externe laissant apparaître une bouillie crayeuse d’urate monosodique, Tahiti, 2019 / *Multiple plantar tophi and external malleolar ulcération revealing a chalky slurry of monosodium urate* (© E. Oehler) 
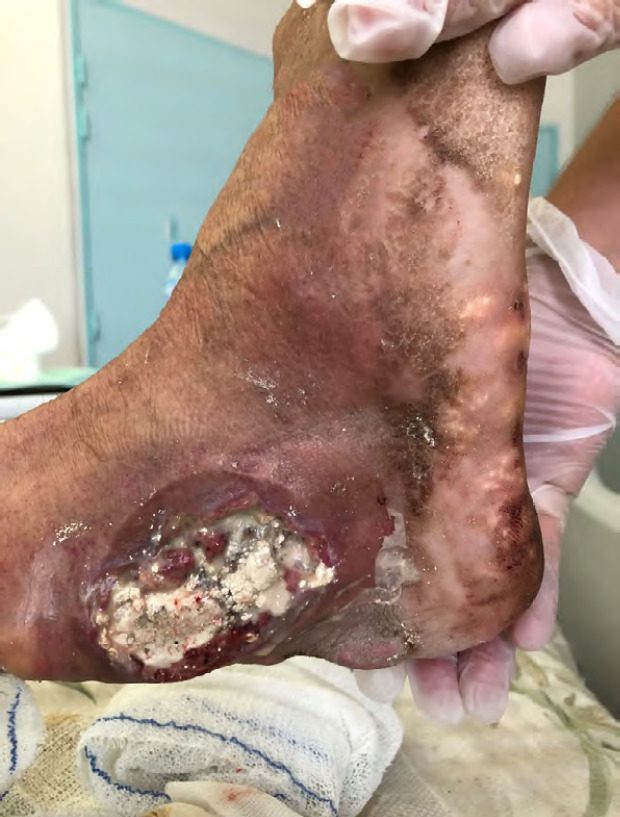

## Pathologies réales

**Ronan Delaval**

Comme dans les autres territoires ultramarins, l’incidence et la prévalence des maladies rénales chroniques (MRC) en Pf est d’évaluation difficile en raison d’un sous-diagnostic et d’une sous-déclaration. En 2021, les patients inscrits en affection de longue durée pour une maladie rénale chronique étaient d’environ 3 700 soit 1,3 % de la population. On peut cependant considérer que la prévalence de la MRC est plus élevée en raison de l’importance croissante des pathologies métaboliques et des données concernant la MRC terminale. L’épidémiologie des MRC au stade terminal est en effet beaucoup mieux connue grâce à la participation aux registres nationaux (Réseau épidémiologie et information en néphrologie, REIN). L’incidence et la prévalence de la MRC terminale traitée est plus de deux fois celle de l’Hexagone avec une incidence de 37/100 000 (contre 16,9) et une prévalence à 296,2/100 000 (contre 136,2). Ces chiffres sont comparables à ceux d’autres territoires insulaires où existe une forte prévalence des pathologies métaboliques telles qu’obésité, diabète ou HTA [202]. Une comparaison de la cohorte polynésienne de néphropathie diabétique biopsiée avec une cohorte de référence métropolitaine (CHU de Nancy), montre un âge à la biopsie inférieur de 10 ans (56 vs 67 ans) et un risque d’insuffisance rénale terminale beaucoup plus rapide (1,6 vs 6 ans). À l’entrée en dialyse, les patients sont en moyenne dix ans plus jeunes qu’en métropole avec une prédominance de patients de moins de 65 ans (données soumises pour publication).

La néphropathie diabétique représente 40 % des causes de MRC terminale chez le dialysé (jusqu’à 65 % au « Centre lourd ») contre 22 % en France toutes régions confondues. Les autres pathologies métaboliques renforcent la tendance avec 12 % de néphropathies supposées d’origine hypertensive. Les glomérulopathies représentent 7,4 % des MRC et les atteintes infectieuses et/ou lithiasiques chroniques 5,7 %. Quinze pour cent des néphropathies sont de cause indéterminée. Enfin, il existe des foyers de pathologies génétiques avec effet fondateur notamment pour le syndrome d’Alport dans l’archipel des Australes, mais également pour des pathologies plus rares avec la plus grosse cohorte décrite de néphropathie caryomégalique (maladie systémique autosomique récessive par mutation de FAN1) et des cas familiaux de hyalinose segmentaire et focale avec variants rares des gènes connus et étudiés [8,113]. Le recours désormais plus systématique à l’exploration génomique permettra de préciser les causes génétiques dans les années à venir [112].

La prise en charge est assurée principalement au CHPf pour les stades pré-suppléance avec une activité de consultation en MRC par les néphrologues hospitaliers, à la fois au CHPf et dans les différents archipels et à la presqu’île de Tahiti. Le service de néphrologie assure donc la prise en charge diagnostique initiale, la prise en charge des insuffisances rénales aiguës, les biopsies diagnostiques, le traitement étiologique et le suivi des MRC. Au stade de suppléance, le CHPf assure exclusivement l’hémodialyse en tant que « Centre lourd » et la transplantation rénale (acte et suivi). Deux structures privées et/ ou associatives prennent en charge l’ensemble des dialyses hors CHPf y compris au domicile (dialyse péritonéale et hémodialyse) et participent aux consultations pour les stades tardifs. Des unités de traitement sont installées à Moorea, Raiatea et Bora Bora alors que les archipels hors îles Sous-le-Vent sont dépourvus de structures de dialyse. La proportion de patients transplantés comparativement aux dialysés n’est que de 30 % alors qu’elle est proche de 50 % en métropole. Ceci est largement expliqué par l’ouverture tardive en 2013 de l’activité de transplantation rénale en Pf et ce malgré une activité comparable à celle de l’Hexagone (environ 50 transplantations annuelles par million d’habitants). Comparativement à la métropole, on observe une proportion plus élevée de patients débutant en urgence la dialyse sur cathéter, reflétant le recours tardif ou la difficulté d’adhésion aux soins. Cette proportion tend néanmoins à se normaliser depuis cinq ans avec le renforcement de l’offre de soins en consultation précoce [202].

À ce jour, les perspectives à court terme sont alarmantes avec un échec des politiques de prévention primaire et une incidence croissante des diabètes compliqués (+7 %/an) qui continuera à impacter l’incidence et la prévalence de la MRC. Le vieillissement de la population représente également un risque épidémiologique à moyen terme. La morbimortalité induite par la pathologie rénale chronique et son impact sur les finances publiques sont des défis de premier ordre en Pf encore plus qu’ailleurs. Ils ne peuvent être combattus qu’en agissant à la fois sur la prévention primaire, en renforçant le tissu de soin aux stades précoces et en planifiant finement les prises en charge en suppléance grâce à une meilleure connaissance épidémiologique.

## Pathologies neurologiques

**Raphaël Buon**

Les pathologies épileptiques constituent une grande partie de l’activité aux Urgences et en consultation. Elles sont rendues complexes par les difficultés d’observance thérapeutique et d’appréhension de la maladie chronique. La consommation de toxiques (alcool, cannabis) est souvent un facteur de fragilité supplémentaire, tout comme la grande précarité sociale.

Les pathologies neurodégénératives liées au vieillissement de la population et la perte d’autonomie qui en découle ne sont pas sans poser de problèmes. Dans une étude de l’INSEE de 2017, 36 % des séniors vivaient dans un ménage composé de plusieurs noyaux familiaux. En Pf, 13 % seulement des séniors vivent seuls (contre 31 % en métropole) : dans la cellule familiale polynésienne, la charge des *matahiapo* (« les anciens ») est en effet souvent dévolue à un jeune sans occupation professionnelle. Culturellement, les troubles cognitifs chez le sujet âgé sont souvent négligés et banalisés, induisant un retard majeur de la première consultation souvent faite au stade démentiel, lors de l’apparition des troubles psycho-comportementaux rendant difficile le maintien à domicile. Au niveau des politiques de santé, malgré l’augmentation du nombre de patients présentant des troubles cognitifs, on note l’absence de Plan Alzheimer, de service de gériatrie au CHPf, d’EHPAD et d’unité cognitivo-comportementale [59]. Ainsi, en l’absence d’infrastructure directement gérée par le Pays, des structures de petite taille appelées « familles d’accueil » se sont développées et prennent en charge les personnes en perte d’autonomie ne pouvant rester à domicile. Il n’existe cependant pas de formation spécifique à la prise en charge des patients déments. Plus récemment se sont créées des structures privées pouvant accueillir une dizaine de pensionnaires, dont le nombre reste encore bien insuffisant au vu des besoins actuels et futurs. Les associations (« Polynésie Alzheimer ») manquent encore de moyens et d’appuis pour soutenir efficacement les patients et leurs familles.

Concernant les pathologies inflammatoires du système nerveux central (SNC), en accord avec le gradient nord-sud bien décrit par ailleurs, aucun diagnostic de sclérose en plaques n’a à ce jour été posé dans la population polynésienne, les patients suivis étant tous originaires de métropole [217]. En revanche, les autres atteintes inflammatoires du SNC sont présentes : pathologies du spectre des neuromyélites optiques (NMOSD) avec positivité des anticorps anti-aquaporine-4, pathologies associées aux anticorps anti-MOG (MOGAD) et encéphalomyélites aiguës disséminées (ADEM). Les encéphalites limbiques (notamment anti-NMDAr) ne sont pas rares, dans des proportions comparables à la métropole. L’éloignement de l’Hexagone rend par ailleurs complexe la prise en charge des patients les plus graves, nécessitant des avis d’experts et des structures non disponibles en Pf comme la neuroréanimation.

Enfin, pour les pathologies neuromusculaires, comme détaillé dans le chapitre concernant les arboviroses, on rappellera l’épidémie de Zika de 2013-2014 responsable d’une explosion du nombre de syndromes de Guillain-Barré (42 patients en six mois) et de chikungunya également accompagnée d’une augmentation des cas de SGB mais dans une moindre proportion [45,176]. L’étude des caractéristiques cliniques et électrophysiologiques ainsi qu’une étude cas-témoin des 42 cas de SGB associés au Zika montraient que les hommes étaient plus souvent concernés (sex-ratio de 2,82) avec un âge moyen de 46 ans et que 88 % avaient présenté une maladie transitoire dans un délai médian de six jours avant l’apparition des signes neurologiques. L’évolution des troubles neurologiques était rapide avec une durée médiane d’installation de 6 jours et de plateau de 4 jours. Plus d’un tiers des patients a nécessité une prise en charge en réanimation, 24 % ont bénéficié d’une trachéotomie, aucun n’est décédé. L’atteinte nerveuse était une neuropathie axonale motrice aiguë dans la totalité des cas. La durée moyenne d’hospitalisation était de 26 jours, la moitié des patients revus en consultation 3 à 4 mois après le début des signes neurologiques marchaient sans aide [45,234].

## Pathologies pulmonaires

**Éric Parrat**

L’asthme est la maladie respiratoire chronique la mieux caractérisée sur le plan épidémiologique en Pf puisqu’elle a bénéficié de trois larges enquêtes en milieu scolaire en 1979, 1984 et 2000. Ces études, basées sur des questionnaires de santé, ont été conduites chez respectivement 3 870, 6 731 et 4 339 élèves du secondaire. L’étude de 1979 montrait une prévalence cumulée d’asthme de 11,5 % chez les adolescents de la communauté urbaine de Tahiti [190]. L’étude de 1984, menée avec la même méthodologie, concluait à une prévalence cumulée de 14,3 %, confirmant les résultats de 1979 et démontrant une augmentation de sa prévalence chez les adolescents de Tahiti [144]. L’enquête ISA AC 2000 *(International Study of Asthma and Allergy in Childhood),* réalisée auprès de 4 339 adolescents sur l’ensemble de la Pf, mettait en évidence une fréquence des sifflements respiratoires de 12,3 %, chiffre situant la Polynésie en zone de forte prévalence d’asthme, dans le tiers supérieur de l’échelle des scores internationaux, au même niveau que les pays d’Europe de l’Ouest [92]. Une autre étude mettait cependant en évidence la sous-estimation induite par la seule considération des sifflements et la réalité bien plus proche des observations de terrain d’une prévalence d’asthme de 20,3 % sur l’estimation obtenue par la compilation d’items cliniques et thérapeutiques [209]. L’asthme est donc une affection très fréquente et en augmentation avec de fortes disparités géographiques, la prévalence allant de 16,7 % aux îles Sous-le-Vent à 32,7 % aux Australes. Du point de vue analytique, l’atopie a été explorée en 1998 sur un groupe de 127 asthmatiques par réalisation de tests cutanés et de dosages des IgE sériques. La sensibilisation cutanée était très élevée (83 %), concordante avec les dosages des IgE. Elle prédominait pour les acariens (80 %), dont *Blomia tropicalis,* acarien spécifique des climats tropicaux (54 %) et à un moindre degré pour la blatte (27 %) [142,185]. Cette étude préliminaire a conduit à la première standardisation internationale de l’allergène *Blomia tropicalis* par un laboratoire pharmaceutique français. La voie de l’atopie a aussi pu être explorée dans le cadre de l’étude ISAAC 2000 par la réalisation de tests cutanés allergologiques chez 603 adolescents. Elle a confirmé que plus des deux tiers (68 %) présentaient un terrain atopique prédisposant et que *B. tropicalis* était le premier allergène concerné (46,7 %). Ces données, au regard de conditions environnementales très favorables, associées à l’amélioration des conditions sanitaires, à l’occidentalisation du mode de vie et à la forte prévalence du tabagisme, mettaient en évidence le très fort potentiel de développement futur de l’asthme en Pf [209]. Ainsi, afin de répondre aux enjeux de santé publique, était créé en 2001 le Centre d’asthmologie du CHPf couplé à un programme de prévention de l’asthme actif entre 2003 et 2008. Ce centre fonctionne selon le modèle des *Asthma training center* nord-américains avec des infirmières cliniciennes spécialement formées à la prise en charge de l’asthme et à son éducation thérapeutique selon le concept des « *Nurses practitioners* » [47,120,156]. Priorité y est donnée à la prise en charge de l’asthme sévère dont les caractéristiques ont été étudiées sur 2 349 consultants entre 2001 et 2003. Les facteurs retrouvés tenaient à l’inflammation bronchique (non observance de la corticothérapie inhalée, asthme hypersécrétant) et à la présence de dilatations des bronches [56]. Aujourd’hui, ce centre participe activement aux études nationales FASE (France Asthme Sévère) du Collège des pneumologues des hôpitaux généraux sur l’asthme sévère afin, notamment, de mieux caractériser cette pathologie et d’en étudier les phénotypes [195,197] FASE-CPHG (France Asthme Sevère -Collège des Pneumologues des Hôpitaux Généraux. Les objectifs sont la mise en place d’une réunion de concertation pluridisciplinaire territoriale des traitements par biothérapies et d’un réseau de santé polynésien d’asthmologie intégrant soin et prévention.

La dilatation des bronches (DDB) est une pathologie respiratoire chronique des bronches également fréquente en Pf. Elle est régulièrement associée à l’asthme et à la bronchopneumopathie chronique obstructive avec lesquels elle partage des symptômes non spécifiques. Dans sa composante pure, elle est estimée à 3,8 % des consultants du Centre d’asthmologie (CHPf, données non publiées) et associée à l’asthme dans 7,1 % des cas [190]. Parmi les étiologies, on trouve régulièrement des DDB post-tuberculeuses et moins fréquemment des déficits immunitaires primitifs de type déficits immunitaires communs variables ou syndrome de Bruton, la mucoviscidose étant quant à elle exceptionnelle en Pf. La très grande majorité des DDB est donc actuellement classée comme idiopathique. Il est de plus possible qu’il s’agisse d’une affection de forte prévalence dans les populations polynésiennes comme le suggèrent des travaux réalisés chez les Maoris de Nouvelle-Zélande [84,228]. L’affection peut évoluer rapidement et conduire à des formes kystiques bilatérales diffuses très sévères. Elle est présente chez des sujets jeunes, pouvant conduire précocement au stade d’insuffisance respiratoire chronique obstructive oxygénodépendante et à ses complications. Plusieurs patients polynésiens sont ainsi engagés dans un projet -ou ont déjà bénéficié-de greffe pulmonaire, uniquement réalisable en France métropolitaine. Vu la complexité du suivi thérapeutique, celui-ci n’est malheureusement pas compatible avec un retour en Pf, ce qui pose d’importants problèmes humains pour les patients et les familles. Des travaux sont actuellement menés en collaboration avec l’équipe universitaire de pneumologie du Centre hospitalier intercommunal de Créteil sur la caractérisation des formes idiopathiques de DDB avec une étude génétique actuellement en cours. En 2021, l’équipe de néphrologie du CHPf a identifié des cas de néphrites interstitielles caryomégaliques avec mutation FAN1 caractérisées par des atteintes multiviscérales, rénales, hépatiques, ORL et pulmonaires. Ces atteintes pulmonaires à type de DDB et/ou de pneumopathie interstitielle paraissent grever le pronostic de l’affection dont l’évolution est souvent redoutable [112].

## Pathologies thyro・iennes

**Sébastien Nunez**

L’incidence du cancer de la thyroïde augmente en Pf comme ailleurs, augmentation liée en partie à un surdiagnostic dû à un recours accru au dépistage échographique. Sur la période allant de 1998 à 2002, cette incidence évaluée à 37,4/100 000 était la plus élevée au monde [28]. En 2014, cette incidence était deux fois plus élevée en Pf qu’en Nouvelle-Zélande où il était observé des différences significatives interethniques avec une atteinte plus fréquente chez les patients insulaires du Pacifique (18,5/100 000) que chez les Maori (Polynésiens) (8,3/100 000) et les patients d’ascendance européenne (5,2/100 000) [157]. Après la guerre d’Algérie, la France établit le Centre d’expérimentation du Pacifique en Polynésie et effectua ses premiers essais nucléaires sur les atolls de Moruroa (aussi transcrit en Mururoa) et Fangataufa (Tuamotu) en 1966. Entre 1966 et 1974, 46 essais aériens furent réalisés puis 147 essais souterrains entre 1975 et 1996. L’hypothèse d’un lien entre ces essais et l’incidence élevée des cancers de la thyroïde a donc naturellement été soulevée. Des études réalisées en Nouvelle-Calédonie ont cependant montré que cette incidence y était plus élevée qu’en Pf alors qu’elle n’avait pas connu d’essais nucléaires [63]. L’incidence des cancers thyroïdiens en Pf est par ailleurs quasi stable entre les groupes de sujets nés avant ou après 1950, plaidant contre une imputabilité majeure de ce sur-risque lié aux retombées radioactives qui n’ont concerné que les sujets nés après 1950. Les retombées radioactives ne seraient ainsi à l’origine que de 29 cas de cancer soit 2,3 % des 1 524 cas attendus entre 1971 et 2070 dans la population polynésienne [74]. En effet, de nombreux autres facteurs de risque sont impliqués, comme les antécédents familiaux ou personnels de pathologie thyroïdienne bénigne ou maligne, la carence iodée avec endémie goitreuse ou l’obésité. L’apport alimentaire en iode, les perturbateurs endocriniens, l’activité physique, un nombre élevé de grossesses et certains facteurs génétiques sont également des facteurs de risque potentiels du cancer de la thyroïde [31,32,33,61,62,74,147,200,237].

Aucune étude spécifique relative aux pathologies thyroïdiennes bénignes n’existe en Pf mais il semble que la fréquence des hyperthyroïdies et des goitres y est bien plus élevée que dans l’Hexagone. Une étude a par ailleurs confirmé que la fréquence de la maladie de Basedow était plus élevée chez les militaires américains originaires de la zone Asie/Pacifique que chez les patients d’origine caucasienne (RR 2 chez les femmes et 4 chez les hommes) [152]. Ces hyperthyroïdies sont par ailleurs souvent compliquées de paralysie périodique thyrotoxique ou de cardiothyréoses possiblement corrélées avec la prévalence de la cardiopathie rhumatismale (observations personnelles).

## Obstétrique

**Stéphane Sauget**

### Épidémiologie des grossesses

Le taux de croissance de la population polynésienne est en baisse (variation de - 0,7 % par an) et le taux de fécondité à 1,7 enfant par femme est également en baisse depuis les 20 dernières années comme dans la plupart des pays industrialisés. Le nombre annuel de naissances a donc ainsi baissé de près de 10 % en 15 ans. Le taux de grossesse chez les femmes mineures est en revanche trois fois supérieur à celui de la métropole avec, selon un rapport publié par la Direction de la santé et le ministère de l’Éducation, près de 40 % des élèves entre 13 et 17 ans qui déclaraient avoir déjà eu des rapports sexuels, 30 % d’entre eux ayant eu le premier rapport avant l’âge de 14 ans. À peine plus de la moitié avait utilisé un préservatif au cours de ce premier rapport sexuel [80,190,213]. Les accouchements sont principalement pratiqués à Tahiti, dans la maternité hospitalière de type 3 du CHPf, les deux maternités privées de type 1 de Papeete et la Maison de naissance de Pirae. En dehors de Tahiti, ils sont également possibles dans la maternité publique de type 2a de Raiatea aux îles Sous-le-Vent et au centre de naissance de Nuku-Hiva aux îles Marquises. L’île de Moorea dispose d’un centre de périnatalité de proximité n’assurant pas les accouchements mais suivant les grossesses et accueillant les accouchées en *post-partum.* Les suivis cliniques et échographiques sont également assurés par les praticiens libéraux (médecins généralistes et sage-femmes), les sages-femmes des centres de protection maternelle de Tahiti, des Australes et des Marquises.

Des consultations spécialisées avancées sur les îles les plus peuplées, organisées par le CHPf et la DS, permettent aux gestantes de bénéficier d’un suivi obstétrical complet et régulier et de ne quitter leur île que pour la fin de la grossesse et l’accouchement. Les patientes des îles ne disposant d’aucune ressource obstétricale fixe ou mobile bénéficient de trois trajets aériens vers Tahiti pour les consultations et échographies de 12 et 22SA et pour l’échographie du troisième trimestre après laquelle elles demeurent sur Tahiti en attendant la naissance.

Cette filière de soin a largement prouvé son efficacité et fait reculer la morbimortalité maternelle pour la ramener aux standards métropolitains où l’accès aux soins obstétricaux ne présente pas les mêmes contraintes. La mortalité néonatale et le taux de prématurité restent cependant 1,5 fois supérieurs à ceux de l’Hexagone [125]. Carences éducatives, violences conjugales et précarité économique et psychologique limitant l’accès aux soins, restent les principaux freins à l’amélioration de ces indicateurs.

## Grossesses pathologiques

### Complications non infectieuses des grossesses

Le taux de menace d’accouchement prématuré (10 %), d’hypertension gravidique (5 %) et de prééclampsie (2-3 %) est sensiblement comparable à celui de l’Hexagone.

Depuis de nombreuses années, on constate une augmentation majeure de la prévalence de l’obésité et du diabète, secondaire aux changements des habitudes alimentaires, ainsi que de leur cortège de complications obstétricales et néonatales qui constituent actuellement un enjeu majeur de prévention en santé publique.

L’anémie gravidique touche plus de 20 % des gestantes. Il s’agit le plus souvent d’anémies ferriprives, parfois très sévères, essentiellement carentielles en raison d’une alimentation inadaptée pendant la grossesse.

Les complications maternelles en cas de cardiopathie rhumatismale apparaissent principalement en cas de valvulopathie méconnue, notamment de rétrécissement mitral sévère (œdèmes pulmonaires inauguraux) qui nécessitent parfois des commissurotomies percutanées en urgence relative en cours de grossesse.

### Grossesses pathologiques : complications infectieuses

La pathologie infectieuse, fréquente en Pf, n’épargne pas les femmes enceintes :

- infections sexuellement transmissibles : on observe une prévalence très importante des infections à *Chlamydia* et à gonocoque dont le dépistage doit être systématique pendant la grossesse [24]. La syphilis est en recrudescence depuis ces dernières années avec, depuis 2021, un cas annuel de fœtopathie syphilitique à haut risque de mort fœtale *in utero* et de décès ou séquelles néonatales [172];
- *Human papillomavirus* (HPV) : il est probable que la prévalence des HPV soit sous-estimée dans cette population fortement tabagique, faute de dépistage systématique tel qu’organisé en métropole;
- arboviroses : les épidémies récurrentes de dengue ont pour conséquences sur les grossesses une augmentation de la prématurité, des morts fœtales et des hémorragies de la délivrance dans les cas graves [19]. L’épidémie de Zika de 2013-2014 a entraîné plusieurs cas de microcéphalies, malformations cérébrales et retards psychomoteurs à long terme [49]. L’épidémie de chikungunya en 2014-2015 s’est quant à elle accompagnée d’un faible nombre d’encéphalopathie néonatale;
- leptospirose : cette infection en cours de grossesse reste peu fréquente mais susceptible d’avoir des conséquences sur celle-ci (fausses couches spontanées, morts fœtales, infections congénitales) [215].

La prise en charge des grossesses en Pf reste donc un défi médical, logistique et financier malgré les moyens déployés et les progrès réalisés, et nécessite la mise en place de protocoles adaptés.

**Figure 40:** Données d’incidence des tumeurs malignes en fonction de la période en Polynésie française [127] / *Data on the incidence of malignant tumors according to the period in French Polynesia [127]* 
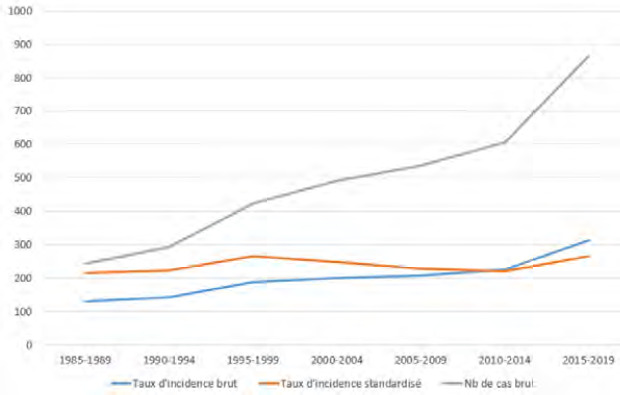

## Cancers et hémopathies malignes

### Généralités

**Pierre Gustin, Philippe Genet**

Jusqu’au début des années 2000, les patients polynésiens atteints de cancer étaient pris en charge en métropole. Désormais, l’activité de soins en cancérologie et hématologie est centralisée au CHPf. En 2015, un Plan cancer polynésien calqué sur le Plan cancer hexagonal a permis de poser les jalons de l’organisation de la prise en charge du cancer en Pf. L’activité de chimiothérapie est réalisée principalement au CHPf mais, depuis 2017, deux sites délocalisés à l’hôpital d’Uturoa (Raiatea, îles Sous-le-Vent) et Taravao (presqu’île de Tahiti) permettent également de réaliser ces traitements au plus près du domicile des patients. Deux nouveaux sites de chimiothérapie délocalisée sont en projet à l’hôpital de Taiohae (Hiva Oa, archipel des Marquises) et Moorea (îles du Vent). La création en 2022 d’un Institut polynésien du cancer (ICPF) a permis d’organiser les activités de recherche clinique, de dépistage, de coordination (Centre de coordination en cancérologie) et de mettre en place un Registre du cancer au sein d’une entité unique spécialement dédiée. L’activité de radiothérapie s’est également développée progressivement permettant, depuis 2021, la prise en charge d’environ 800 patients par an sur les deux accélérateurs linéaires du service d’oncologie-radiothérapie. Les techniques modernes de radiothérapie en modulation d’intensité, radiothérapie guidée par l’imagerie et radiothérapie en condition stéréotaxique y sont réalisées. En 2024, l’ensemble de l’activité d’anatomopathologie de Pf devrait être centralisé au sein d’un centre unique d’anatomopathologie sous l’égide de l’ICPF, une activité de curiethérapie à haut débit de dose devrait démarrer et éviter ainsi aux patients des évacuations sanitaires vers les centres métropolitains et un PET-scan devrait être installé.

Un récent rapport de l’OMS indique que l’incidence du cancer devrait doubler dans le Pacifique insulaire entre 2018 et 2040 et cette tendance est également observée en Pf [222]. Un rapport du Registre du cancer de l’ICPF a récemment montré que l’incidence annuelle moyenne en Pf entre 2015 et 2019 était de 865 cas (806 tumeurs solides et 58 hémopathies malignes) soit un taux d’incidence annuel standardisé de 269,9/100 000 chez les hommes et 261,7/100 000 chez les femmes (Fig. 40). Ce taux reste pour le moment inférieur à celui de la France hexagonale, la différence étant plus marquée chez les hommes, et s’explique par une population globalement plus jeune [162]. Cette incidence augmente cependant d’environ 5 % par an, accompagnant le vieillissement global de la population [127]. La répartition du nombre de cas par archipel sur la période 20152019 comparée à la répartition de la population sur cette même période ne présente pas de différence statistiquement significative.

Avec un total de 414 décès et un taux de mortalité standardisé de 123,8/100 000, les tumeurs malignes étaient la deuxième cause de décès en Pf (26 %) en 2017, à quasi-égalité avec les maladies cardiovasculaires. Cette observation est identique depuis 2005 [240]. Chez les hommes, on comptabilisait 214 décès, soit un taux de mortalité standardisé de 129,9/100 000, représentant la deuxième cause de décès (23,4 %) après les décès par maladies cardiovasculaires (28,1 %). Chez les femmes, on comptabilisait 200 décès, soit un taux de mortalité standardisé de 199,5/100 000 représentant la première cause de décès (29 %) devant les décès par maladies cardiovasculaires (25,5 %). Cette tendance ayant beaucoup fluctué, elle devra être consolidée dans les prochaines années. La mortalité par cancer est plus élevée que dans l’Hexagone en raison d’une prise en charge généralement plus tardive et à un stade plus avancé de la maladie dont les raisons sont multiples : spécificités géographiques, insularité et éloignement des professionnels, faible adhésion au dépistage et/ou au traitement qui entre parfois en concurrence avec les traitements traditionnels… Avec un âge médian de 66 ans chez l’homme et 58 ans chez la femme, le diagnostic de cancer est également plus précoce et pourrait s’expliquer par l’absence de contrôle de certains facteurs de risque comme le tabagisme et/ou le terrain d’hyperœstrogénie induit par l’obésité [127].

## Cancers solides

**Pierre Gustin, Shari-Lane Botche**

Les cancers les plus fréquents sont les cancers du sein, de la prostate et du poumon avec respectivement 164, 128 et 124 cas annuels (Fig. 41). La répartition topographique des cancers est détaillée Figure 42 et Figure 43.

Il existe certaines spécificités territoriales, notamment le carcinome hépatocellulaire qui a fait l’objet d’une description rétrospective de 139 cas diagnostiqués entre 2008 et 2017 avec une incidence globale de 8,5/100 000 (13,5/100 000 chez l’homme) et de 43,1/100 000 aux îles Australes. Ceci fait de cet archipel l’une des régions à plus forte incidence au monde. Il faut préciser que ce cancer était toujours associé à une infection au virus de l’hépatite B [136]. Le cancer de l’endomètre fait quant à lui l’objet d’une attention particulière (cohorte rétrospective EARLY) du fait de sa spécificité territoriale caractérisée par une incidence élevée et un âge au diagnostic de 20 ans plus jeune qu’en métropole *(Endométrial cAncer in fRench polYnesia: the EARLY study.* Epaillard *et al.* WHE-23-0343, on review). Enfin, certains cancers tels que le cancer du côlon sont moins fréquents qu’en métropole [127].

**Figure 41:** Nombre moyen annuel des tumeurs malignes solides par topographies les plus fréquentes, Polynésie française, période 2015-2019 [127] / *Average annual number of solid malignant tumors by the most frequent topographies, French Polynesia, period 2015-2019 [127]* 
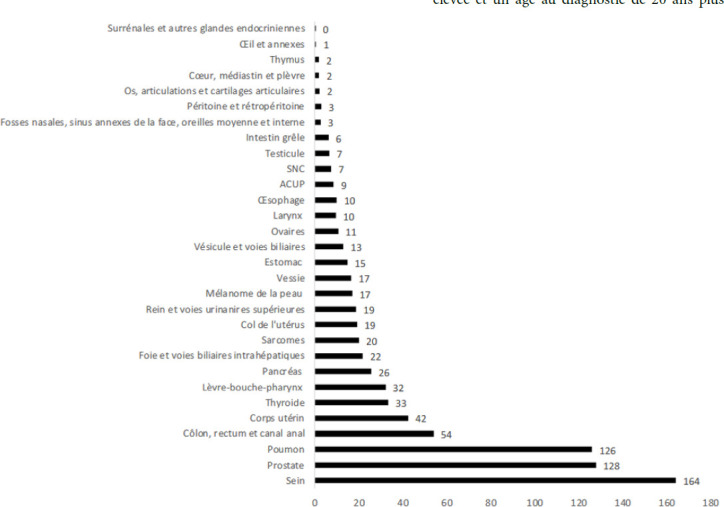

**Figure 42:** Répartition des cas de tumeurs solides par topographie chez la femme, Polynésie française, 2015-2019 [127] / *Distribution of cases of solid tumors by topography in women, French Polynesia, 2015-2019* [127] 
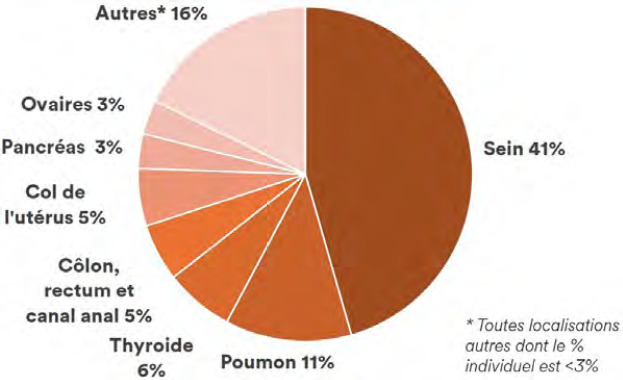

**Figure 43:** Répartition des cas de tumeurs solides par topographie chez l’homme, Polynésie française, 2015-2019 [127] / *Distribution of cases of solid tumors by topography in men, French Polynesia, 2015-2019 [127]* 
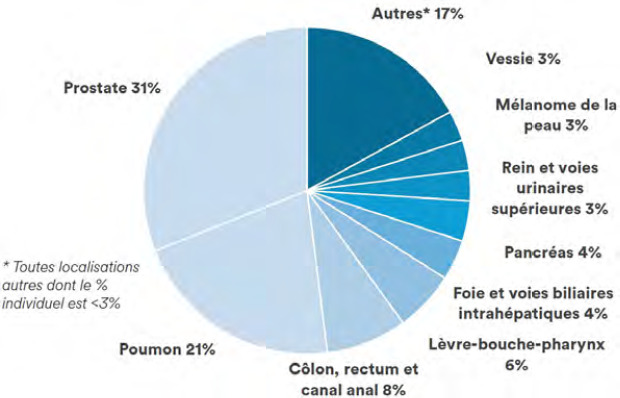

Depuis 2019, une réunion de concertation pluridisciplinaire commune intercontinentale (RCP Pacifique) avec l’Institut Gustave Roussy (Villejuif) et le service d’oncologie de la clinique Kuindo-Magnin de Nouméa (Nouvelle-Calédonie), permet de discuter des dossiers complexes et tumeurs rares et d’organiser si nécessaire une évasan vers un centre de référence en métropole.

## Hémopathies malignes

**Philippe Genet**

Les données épidémiologiques sont relativement parcellaires pour les hémopathies malignes avant le début des années 2000 : pour les leucémies aiguës myéloblastiques (LAM) par exemple, une étude couvrant les années 1998 à 2002, montrait un taux d’incidence standardisé de 6/100 000, constituant à cette époque l’incidence la plus élevée au monde [28]. Une étude réalisée entre 1986 et 2001 montrait quant à elle une incidence des leucémies de la population native polynésienne globalement similaire à celle observée à Hawaï, un taux plus faible de leucémies lymphoïdes chroniques et une forte incidence des LAM chez les Marquisiens laissant envisager l’hypothèse génétique ou de conséquences des essais nucléaires [206]. L’activité du service d’hématologie est un reflet fidèle de l’épidémiologie des hémopathies malignes en Pf puisque l’intégralité de celles-ci y est suivie. Au cours de l’année 2022, 523 patients atteints d’hémopathies malignes ont ainsi été vus au moins une fois dans le service, le diagnostic ayant été porté cette même année pour 121 d’entre eux. L’âge médian de ces patients était de 63,4 ans et le sex-ratio était de 1,1 (251 femmes et 272 hommes). Le Tableau II résume la répartition des différents diagnostics. Dans cette cohorte, 119 patients (22,8 %) habitaient une île éloignée de Tahiti ou Moorea nécessitant ainsi un déplacement en avion pour leur suivi (hospitalisation, consultation et/ou bilan biologique spécifique).

La prise en charge des hémopathies en Pf est soumise à différentes contraintes spécifiques en lien avec un plateau technique limité par rapport aux exigences modernes. Ainsi, si le laboratoire d’hématologie du CHPf réalise la cytologie courante (sang et myélogramme) et depuis peu la cytométrie de flux et la recherche et la quantification du transcrit de fusion bcr/ abl, les examens de cytogénétique et de biologie moléculaire sont quant à eux acheminés une seule fois par semaine vers la métropole, entraînant ainsi une possible perte de qualité des échantillons et des délais diagnostiques très allongés. Le laboratoire d’anatomopathologie du CHPf permet une très bonne orientation diagnostique mais ne peut poser un diagnostic de certitude en raison de l’impossibilité de réaliser un immunomarquage exhaustif (absence de mise à disposition de tous les anticorps nécessaires, essentiellement pour des raisons financières). Un transfert hebdomadaire du matériel de biopsie vers un laboratoire métropolitain est donc nécessaire, augmentant par conséquent les délais de restitution des résultats. Le plateau d’imagerie (CHPf ou libéral) permet la réalisation de l’imagerie standard mais présente des délais d’obtention longs par rapport à la métropole : l’IRM corps entier dans le cadre du bilan initial d’un myélome est par exemple remplacée systématiquement par un scanner du fait de délais pouvant atteindre plusieurs mois, de difficultés de recrutement de radiologues, notamment interventionnels, compliquant la prise en charge diagnostique… Le PET-scan initial recommandé par les standards internationaux pour les lymphomes n’est le plus souvent pas réalisé en raison de son absence en Pf et seul un PET-scan de suivi est effectué. En raison de toutes ces difficultés, certains patients ne peuvent pas être pris en charge localement, notamment les patients jeunes porteurs de LAM qui sont systématiquement évacués en métropole. Seules les LAM3 et les LAM hyperleucocytaires sont traitées sur place, une évacuation sanitaire paraissant trop risquée du fait de la durée du voyage et du risque de coagulation intravasculaire disséminée et de leucostase. Les allogreffes, les autogreffes et les CAR-T cells sont également réalisées en métropole du fait de l’absence de thérapie cellulaire au CHPf. Ce sont ainsi 37 patients qui ont bénéficié d’une évacuation sanitaire vers l’Hexagone en 2022 et, même si ces évacuations restent nécessaires à la bonne prise en charge du patient, elles ont pour conséquence un traumatisme psychologique important pour des personnes contraintes de s’éloigner pour une longue durée de leur entourage et de leur cadre culturel. Certains patients refusent ainsi parfois cette possibilité d’évasan, quitte à ne pas bénéficier d’une prise en charge optimale. Afin de réduire ces difficultés, le service d’hématologie du CHPf a noué des partenariats avec des structures métropolitaines et notamment le service d’hématologie clinique de l’Hôtel-Dieu puis de l’hôpital Saint-Antoine à Paris, permettant d’obtenir des avis ponctuels urgents et d’inscrire en RCP les patients polynésiens qui bénéficient ainsi de l’expertise d’un CHU. De la même façon, un partenariat a été mis en place avec le service de biologie de l’hôpital Saint-Antoine pour la relecture de lames d’interprétation compliquée, pour la cytogénétique et la biologie moléculaire ou avec le service d’anatomopathologie de l’hôpital Necker pour la relecture de biopsies.

**Tableau II:** Répartition des différents diagnostics d’hémopathies malignes (réalisation : P. Genet, données non publiées) / *Distribution of different hématologie malignancies diagnoses (production: P. Genet, unpublished data)*

**Pathologies**	**Vus en 2022 (%)**	**Diagnostiqués en 2022 (%)**
Myélomes et amyloses AL	80 (15,3)	18 (14,7)
Lymphomes de Hodgkin	22 (4,2)	5 (4,1)
Lymphomes non hodgkiniens	119 (22,7)	33 (27)
Leucémies aiguës (myéloïdes et lymphoïdes)	43 (8,2)	14 (11,5)
Leucémies lymphoïdes chroniques	34 (6,5)	9 (7,4)
Leucémies myéloïdes chroniques	39 (7,4)	3 (2,5)
Myélodysplasies	38 (7,3)	16 (13,1)
Syndrome myéloprolifératifs	148 (28,3)	23 (18,9)

## Pathologies psychiatriques

**Johan Sebti, Moerani Rereao**

Par son étendue et son histoire, la Polynésie française concentre sur un unique territoire administratif un grand nombre de variabilités : culturelles, socio-économiques, linguistiques mais aussi environnementales. Ces contrastes sont exacerbés par la constante de l’insularité, doublement caractérisée par l’éloignement géographique et la limitation des ressources disponibles [22]. De manière significative, les déterminants classiques de la santé mentale tels que le niveau de revenu économique, les relations sociales, l’accès à un logement décent et aux services de santé, peuvent subir des modifications radicales d’un environnement à l’autre. Les besoins en santé mentale s’expriment donc de manière différenciée. Un modèle de soin ou une approche pertinente dans une région particulière ne peuvent pas nécessairement être généralisés à l’ensemble du territoire, ce qui conduit à des efforts d’adaptation et d’innovation importants. De plus, la Pf ne dispose pas à ce j our d’observatoire ou de système de collecte des données en santé mentale. *De facto,* les seules informations disponibles sont majoritairement issues de travaux de recherche ponctuels ou de l’activité de soin du CHPf. Ces données partielles constituent une double difficulté, tant sur la capacité à guider les politiques de soin et de prévention que sur l’identification de particularités locales au plan clinico-épidémiologique. Enfin, l’important stigma social associé à la santé mentale semble contribuer à des retards de soins ou de diagnostic souvent préjudiciables qui accentuent la représentation d’une discipline axée vers les situations de crise.

## Conduites suicidaires

Les comportements suicidaires font l’objet d’une surveillance particulière en Pf, du fait de spécificités anthropologiques et culturelles encore mal comprises. Il s’agit, entre autres, d’un enjeu de santé publique croissant sur la dernière décennie. Le pays a notamment bénéficié de l’appui technique de l’OMS dans le vaste programme SUPRE *(SUicide PREvention)* portant sur la santé mentale en population générale et les conduites suicidaires, à l’origine de plusieurs publications.

### Comportements suicidaires non létaux et tentatives de suicide

Les principales données épidémiologiques sur les tentatives de suicide (TS) trouvent leur source dans deux études de méthodologie similaire, sur des échantillons constitués durant les périodes 2008-2010 et 2020-2023 [5,6]. Une augmentation d’incidence des tentatives a été mise en évidence durant l’intervalle de ces deux périodes, passant de 79,4/100 000 habitants en 2010 à 123/100 000 en 2022. Ces deux études avaient le mérite de se baser sur une inclusion des patients directement depuis le service d’accueil des urgences du CHPf, centralisant la plupart des situations en provenance des archipels. L’étude la plus récente, réalisée en contexte Covid-19, mettait en avant une augmentation nette de l’incidence des TS (+20 %) au cours des trois années de crise sanitaire, principalement sur la période de sortie de crise (2022-2023) en comparaison avec la période initiale (2020-2021).

Les tranches d’âge 10-19 et 20-29 ans représentaient la moitié des sujets (49,3 %). Les femmes jeunes apparaissaient deux fois plus exposées que les hommes jeunes (181,7 *versus* 84/100 000 habitants). Le mode de vie urbain ou périurbain (Tahiti principalement) semblait constituer un facteur de risque supplémentaire (Fig. 44) [214].

Concernant les modes de passage à l’acte, l’intoxication médicamenteuse volontaire (anxiolytiques et antalgiques principalement) était le mode de TS le plus représenté (56,8 %) suivi de la pendaison (19,9 %), des phlébotomies (6,8 %) et des TS avec objets tranchants ou pénétrants (3 %) (Fig. 45). La pendaison apparaissait comme un moyen plus fréquent chez les hommes et dans les archipels, alors que l’intoxication médicamenteuse volontaire était plus fréquente à Tahiti et chez les femmes. Les méthodes violentes de suicide comme la pendaison et l’usage d’objets tranchants, apparaissaient comme une tendance de choix parmi les 20-29 ans par rapport aux autres groupes d’âge [214]. En raison de son caractère « accessible », la pendaison est probablement perçue comme une méthode sûre et efficace, en particulier dans les îles où l’accès à des moyens alternatifs (médicaments, véhicules) peut être plus difficile. De plus, des paramètres liés à la culture ou au genre pourraient influencer le recours à ce type de moyen. Par analogie, dans la culture chinoise, une croyance suggère que les esprits des personnes décédées par pendaison reviennent hanter les vivants, souvent à des fins de vengeance [140]. Il est possible que des croyances culturelles similaires existent dans la culture polynésienne (mythe des *tūpāpau),* en particulier dans les communautés plus traditionnelles, mais aucune donnée n’a été répertoriée sur ce sujet spécifique.

**Figure 44:** Incidences normalisées par âge et localité géographique des TS (2020-2023) (réalisation : Sebti et al., 2023) / *Standardized incidences by age and géographie location of TS (2020-2023) (production: Sebti et al., 2023)* 
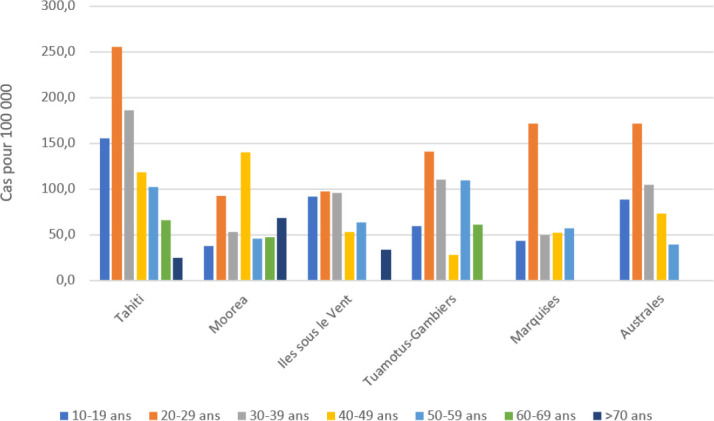

**Figure 45:** Distribution des méthodes de TS par genre 2020-2023 (réalisation : Sebti et al., 2023) / *Distribution of TS methods by gender 2020-2023 (production: Sebti et al., 2023)* 
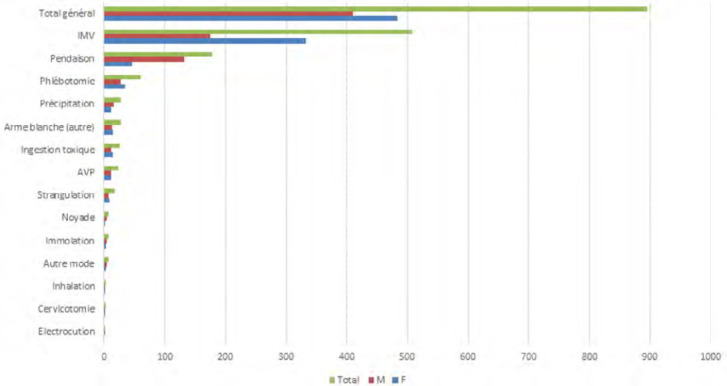

Le taux d’hospitalisation secondaire à une TS était de 36 % sur la période 2020-2023 avec un taux de récidive de 11 % sur les 36 mois de l’étude. La moitié des TS n’était pas rattachée à une comorbidité psychiatrique et, lorsqu’un diagnostic était établi, il s’agissait principalement d’antécédents de comportements suicidaires isolés (15,3 %), d’épisodes dépressifs (9,9 %) et de troubles psychotiques (7,9 %) [214]. Cette dernière étude ne prenait pas en compte les facteurs liés au passage à l’acte, contrairement à celle d’Amadéo *et al.* qui montrait que les problèmes relationnels avec le conjoint (68,6 %), les parents (34 %) ou la présence de symptômes psychiatriques (24 %) jouaient un rôle prépondérant dans l’exécution du scénario suicidaire.

### Suicides complets

Le niveau de risque suicidaire en population générale, à comprendre comme la résultante de plusieurs facteurs de risques spécifiques (présence d’idées auto-agressives, antécédents de passage à l’acte), apparaît anormalement élevé en Pf (26,7 %) en comparaison de l’Hexagone (13,7 %). Les facteurs contributifs à ce risque global sont l’âge jeune (18-29 ans), les séparations ou divorces, la connaissance de légendes sur le suicide et l’exposition à des évènements traumatiques. *A contrario,* la pratique religieuse ainsi que l’accès à des soins traditionnels semblent représenter des facteurs de protection dans l’échantillon étudié [4]. Contrairement à l’Hexagone, la Pf ne dispose pas de système de veille épidémiologique active des décès par suicide. Les seules données disponibles sont celles fournies par une étude épidémiologique sur des données rétrospectives sur les décès par suicide de 1992 à 2016 [5,6,238]. Une sous-estimation des décès par suicide de 20 % en moyenne a par ailleurs été constatée [6].

Un décès par suicide peut entraîner des difficultés supplémentaires (culpabilité, dépression…) chez les proches endeuillés. Selon Wrobleski *et al.,* il touche en moyenne 10 proches mais, en Pf, du fait de la promiscuité, l’impact psychologique sur la population semble plus important [236]. En Pf, on observe en moyenne 32 décès par suicide par an, soit un taux de mortalité de 9,2/100 000 entre 2011 et 2016. L’âge moyen était de 34,4 ans sur la période 2000-2014 avec un sex-ratio à 3,2. La majorité était des personnes mariées ou en concubinage (40,2 %) et 19,3 % étaient célibataires; 6,3 % avaient un emploi au moment du suicide. Le taux de décès par suicide en Pf en 2016 était inférieur à celui de la France métropolitaine (8,3 *versus* 12,1/100 000) (Fig. 46). Les personnes âgées sont peu nombreuses à passer à l’acte (2 suicides chez les 65 ans et plus entre 2005-2010), peut être en lien avec la densité du tissu social qui est encore importante en Pf [6,238].

Parmi les facteurs de risque on trouve les troubles psychiatriques dans 32 % des cas dont les troubles de l’humeur (14,2 %), les addictions (7,3 %), les troubles psychotiques (5,4 %) pour lesquels les hommes sont les plus représentés et les troubles de la personnalité (2,5 %). La méthode la plus utilisée, tous sexes confondus, est la pendaison (87,3 %), largement devant l’intoxication médicamenteuse (3,8 %) [6]. Parmi les facteurs déclencheurs du geste suicidaire, les problèmes conjugaux apparaissaient en tête de liste (30,3 %). Enfin, le caractère prémédité du geste n’était retrouvé que pour 16,5 % des individus.

À plus large échelle, ces études ont permis de suspecter un lien entre mortalité par suicide et situations de crise. Les périodes 2005 et 2008 ont notamment été marquées par une instabilité politique en Pf ainsi qu’une crise économique mondiale, avec une légère augmentation de la mortalité. À l’inverse, la période 2009-2011 qui correspond à l’implantation du Programme de prévention des suicides de l’OMS (SUPRE) s’accompagne d’une diminution des cas. Des actions préventives ont été mises en place avec la création du Centre de prévention du suicide qui propose des suivis psychologiques brefs, des approches traditionnelles et des groupes de soutien aux endeuillés. De ces postulats, il est attendu que la crise sanitaire liée à l’épidémie de Covid-19 ait elle aussi contribué défavorablement à la mortalité par suicide en Polynésie française.

Des colloques et conférences ont été régulièrement organisés à Tahiti en liaison avec des experts de l’OMS et de l’Association internationale de prévention du suicide (AIPS) afin de réfléchir à la mise en place des stratégies de prévention les plus adaptées, à la fois celles qui ont fait leurs preuves dans le monde et celles qui pourraient être plus spécifiques à la situation socioculturelle et psychologique de Pf : le premier Congrès international de psychiatrie (2011), l’atelier OMS sur la surveillance épidémiologique et la prévention du suicide (2013), la Conférence régionale Asie-Pacifique de l’AIPS, la Journée de santé mentale en 2018 en présence de Patricia Speelman, directrice du Centre de prévention du suicide de Los Angeles et du Pr Pierre Thomas, coordinateur national des Centres 3114 (numéro national Prévention du suicide).

Face au constat d’un taux de suicide qui ne diminue pas autant que celui de la France métropolitaine, l’équipe MOODS du Centre de recherche en santé des populations mènera l’étude *Apport de l’autopsie psychologique à la compréhension des conduites suicidaires en Outre-mer (Autopsom)* sur plusieurs sites ultramarins dont la Pf, et un site de la France hexagonale. Cette étude aura pour objectif de mettre en place un système de surveillance épidémiologique du suicide et d’identifier les facteurs de risque de décès spécifiques et communs à ces territoires. Le recueil des données permettra de mieux comprendre les conduites suicidaires et de mettre en place des programmes de prévention adaptés [201].

## Schizophrénies et psychoses apparentées

### Psychoses aiguës et émergentes

La création d’une équipe spécialisée en intervention précoce pour les troubles psychotiques émergents (ESPER) a permis, dès 2020, la collecte des premières données épidémiologiques et cliniques sur les épisodes psychotiques inauguraux en Pf. Pour rappel, l’évolution classique d’un premier épisode psychotique donne lieu dans 40 à 50 % des cas à un trouble psychotique constitué, dans 30 % à un trouble de l’humeur et dans 20 % une évolution favorable sans récidive. Le repérage et l’intervention thérapeutique précoces permettent souvent une bien meilleure trajectoire en termes de pronostic social et fonctionnel, associée à une réduction des rechutes [66].

**Figure 46:** Taux de mortalité par suicide en Pf, Nouvelle-Calédonie et France métropolitaine (2000-2016) (réalisation : Direction de la Santé de Pf, DASS de la NC et CépiDC) / *Suicide mortality rate in Pf, New Caledonia and mainland France (2000-2016) (production: Pf Health Department, DASS of NC and CépiDC)* 
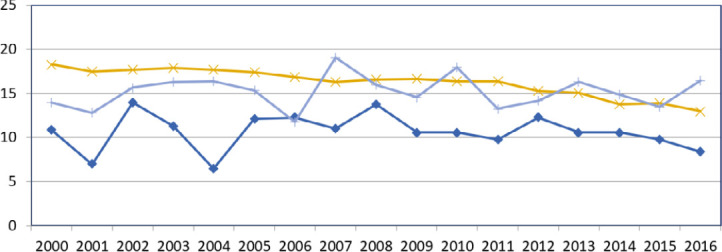

Un total de 130 patients présentant un premier épisode psychotique a pu être inclus sur ce dispositif entre 2020 et 2023. L’incidence annuelle standardisée pour la classe d’âge 1635 ans sur la période 2022-2023 était estimée

à 49/100 000 habitants, comparable à la France métropolitaine. En 2023, environ 30 % des premiers épisodes psychotiques en Pf étaient accessibles à une prise en charge exclusivement ambulatoire lors du premier contact (CHPf, données non publiées). Le sexe masculin semble représenter un facteur de vulnérabilité particulièrement marqué, avec un sex-ratio de 3 en Pf, bien supérieur aux valeurs usuelles de la littérature de 1,4 [2]. L’âge moyen au moment de la prise en charge était sensiblement similaire pour les deux genres (22 ans) avec une durée de psychose non traitée plus faible pour les femmes (8 *versus* 11 mois).

La durée de psychose non traitée, principal facteur pronostique dans le cadre des troubles psychotiques débutants, fait l’objet d’une grande hétérogénéité sur le territoire. La moyenne enregistrée sur Tahiti était de 8,9 mois pour les hommes et 8,5 mois pour les femmes. Dans les archipels, elle peut atteindre des valeurs bien supérieures, 32 mois par exemple pour les îles Sous-le-Vent, liées en grande partie aux difficultés de repérage, d’adressage mais aussi d’une stigmatisation fortement associée à la maladie psychique. L’association, parfois à un âge précoce, à un usage régulier de cannabis, est retrouvée dans plus de 90 % des cas. Les cas de « pharmacopsychose cannabique » spontanément résolutifs, et parfois déclenchée à l’occasion d’un sevrage brutal, ne sont pas rares, mais témoignent souvent d’un niveau de vulnérabilité accru et peuvent donner lieu à l’installation d’un trouble psychotique plus durablement constitué à distance. Il n’existe pas à ce jour en Pf de données précises sur le taux de transition vers un trouble psychotique chronique après un premier épisode faute de recul encore suffisant sur cette activité.

### Psychoses chroniques

Les seules données disponibles sont celles apportées par l’étude *Santé mentale en population générale* (SMPG) qui estimait une prévalence des troubles psychotiques à 3,7 % de la population générale, bien supérieure aux valeurs de la littérature [4,211]. Les schizophrénies restaient les troubles psychotiques chroniques les plus représentés dans cette étude, avec des expressions symptomatiques assez larges. En Pf, les thèmes de délire sont assez éclectiques et souvent liés au mode de vie et à l’identité culturelle du patient. Les délires paranoïaques, de surveillance ou fantastiques seraient davantage retrouvés à Tahiti alors que les archipels seraient un terrain plus favorable à la structuration de thématiques mystiques, mégalomaniaques ou magiques [104]. La réponse aux traitements antipsychotiques a longtemps fait l’objet de spéculations, avec le postulat que les patients d’origine polynésienne présenteraient davantage de critères de résistance pharmacologique. Cette hypothèse tend de nos jours à être réfutée, sur la base de l’analyse des taux plasmatiques résiduels de certaines molécules, comme la clozapine et la N-desméthylclozapine, qui orienteraient au contraire vers une plus large proportion de patients métaboliseurs lents en comparaison des populations européennes (CHPf, données non publiées). L’évolution observée des troubles psychotiques est sensiblement la même qu’en France métropolitaine, mais avec un pronostic social souvent grevé par la pauvreté des solutions d’accompagnement professionnel ou médicosocial.

## Psychotraumatologie

Le psychotraumatisme, défini comme une perturbation aiguë ou durable, plus ou moins intense, de l’équilibre psychique, représente une entité peu documentée en Pf mais qui bénéficie d’un regain d’intérêt certain depuis la crise Covid-19. La confrontation à une situation violente, la peur pour sa propre vie, une menace pour son intégrité ou celle d’un proche, sont les principaux pourvoyeurs de psychotraumatisme. Il représente un facteur de risque spécifique pour le développement d’un trouble de stress posttraumatique, et non spécifique pour les autres troubles psychiques ou les conduites addictives. L’étude SMPG retrouvait une prévalence vie-entière de 44 %, souvent secondaire à des faits de violences sexuelles ou physiques [5].

La pratique clinique en Pf tend à confirmer cette prévalence anormalement élevée des évènements psychotraumatiques, souvent passés sous silence et révélés à l’occasion de complications anxieuses ou dépressives qui amènent à consulter un spécialiste. Outre l’accidentologie classique (accidents de personnes, intempéries…), les violences physiques ou sexuelles répétées, notamment dans le cadre intrafamilial, semblent en constituer le principal substrat et donnent fréquemment lieu à une structuration de type trauma complexe. Les troubles de stress post-traumatique plus « typiques », à l’occasion de traumatismes ponctuels (découverte de personnes suicidées ou accidents de la voie publique notamment) ne sont pas rares, mais empruntent souvent une présentation initiale tronquée (troubles du sommeil, trouble anxieux généralisé ou tableau psychotique) qui, à défaut d’en rechercher l’origine traumatique, conduira souvent le clinicien en erreur.

Des efforts de documentation et de prévention semblent indispensables dans ce domaine qui relève potentiellement d’un problème de santé publique.

## Troubles de l’humeur

On estime que la prévalence de la dépression unipolaire est relativement faible en Pf, évaluée entre 8,5 et 18,2 % dans l’étude SPMG et comparable à celle de la métropole (9,8 à 13,3 % en 2021) [141]. Sur la base de la pratique clinique, ce taux apparaît en revanche plus faible, possiblement par un phénomène de sous-diagnostic ou de réticence à consulter [201]. La faible incidence des syndromes dépressifs enregistrée en consultation pourrait aussi s’expliquer par des aspects culturels et linguistiques. Le mot « dépression » n’ayant pas d’équivalent en langue polynésienne, le *«fiu* » (nonchalance, découragement, lassitude ou détachement causé par l’environnement ou les états d’âme) en serait sa plus proche traduction. Une des hypothèses serait que l’équivalent clinique de la dépression dans la culture polynésienne puisse échapper aux critères diagnostiques classiques (tristesse, anhédonie, temporalité) et se manifester à travers d>autres dimensions, telles que des difficultés à maintenir le contrôle des impulsions ou ses relations sociales. Ce sujet a été exploré dans une autre publication de Amadeo *et al.* sur les représentations de la maladie mentale en Pf qui nous éclaire sur la perception sociale du « dépressif » en Polynésie française : quelqu’un reconnaissable à son apparence, souffrant de problèmes sentimentaux ou relationnels, dont la souffrance s’élargit à la sphère familiale, mais pouvant espérer une guérison (parfois contrainte) [4].

## Troubles liés à l’usage de substances psychotropes hors tabac

En Pf, les principales substances concernées sont l’alcool, le cannabis (« pakalolo »> ou « paka ») et la méthamphétamine (« ice » ou « sana »). L’étude STEPS 2019 mettait en avant la progression de la consommation d’alcool (> 60 g/jour) dans les deux sexes, passant de 4,2 % en 2010 à 7,9 % en 2019. Cette progression se retrouvait également pour les consommations quotidiennes comprises entre 40 et 60 g/jour. Concernant la consommation de cannabis, 39 % des adultes rapportaient au moins une consommation sur les 12 derniers mois, contre 23 % en 2010. Parmi eux, 42 % déclaraient des prises quasi quotidiennes [82].

Concernant les plus jeunes, l’enquête *Ea Taure‘a* de 2020, pratiquée lors de la Journée d’appel de préparation à la défense auprès de 3 930 jeunes participants de 16 à 25 ans portait également sur les habitudes de consommation de toxiques de cette classe d’âge : 92 % des sondés rapportaient au moins une prise d’alcool sur les 30 derniers jours, 33 % au moins une consommation de cannabis et 2 % une consommation d’ice [79]. D’après l’enquête *Ea Taure‘a,* deux profils de jeunes consommateurs pouvaient être établis selon l’agrégation des scores [79] :

- les « non ou monoconsommateurs » (75 %), principalement des jeunes femmes scolarisées âgées de 16 à 17 ans, débutant une consommation vers 16 ans portant surtout sur l’alcool avec peu d’ivresses rapportées, et un entourage décrit comme soutenant et bienveillant;
- les « multiconsommateurs » (24 %) représentés plutôt par des hommes de 18 ans et plus, non scolarisés, vivant dans les archipels éloignés avec des ivresses multiples et fréquentes associées à des troubles du comportement (agressivité et rixes), une consommation débutant avant 16 ans, souvent accompagnée par des amis et décrivant un environnement relationnel peu soutenant voire conflictuel.

## Médecine hyperbare

**Loïc Durand**

En 1966, un caisson hyperbare (COMEX RD 15) a été implanté à Tahiti. Utilisé par les militaires, sa vocation première était de prendre en charge les travailleurs sous-marins œuvrant pour le Centre d’expérimentation du Pacifique. Lors de la fermeture de l’hôpital militaire en 1998, l’activité hyperbare a été reprise par les urgentistes de l’hôpital civil, futur CHPf. Cette activité ne prenait en compte que le traitement des urgences relevant de l’oxygénothérapie hyperbare (accident de décompression, intoxication au monoxyde de carbone, gangrène gazeuse, embolie gazeuse et surdité brusque de haut niveau), représentant environ 150 séances par an (dont une vingtaine d’accidents de plongée). Au début des années 2000, le développement du tourisme a permis de sensibiliser les décideurs politiques, permettant le remplacement de l’ancien caisson par un COMEX 2500 qui est encore utilisé aujourd’hui. Il est composé de deux chambres thérapeutiques contenant chacune six places assises (ou un brancard et deux places assises) avec un sas central entre les deux (Fig. 47).

En 2018, du fait de la forte prévalence des maladies métaboliques et infectieuses, les indications ont été élargies aux autres recommandations de l’HAS de 2007. Ainsi, des pathologies nécessitant une cicatrisation dirigée telle que l’infection de pied diabétique, les lésions radio-induites et les plaies chroniques ischémiques non diabétiques ont pu bénéficier d’un traitement par oxygénothérapie hyperbare. La calciphylaxie, pathologie non listée par les recommandations de l’HAS, fait également l’objet d’une prise en charge au caisson hyperbare puisqu’il a été montré que la cicatrisation de celle-ci était accélérée, dans le cadre d’un protocole néphrologique spécifique associant une séance quotidienne d’oxygénothérapie hyperbare, une hémodialyse quotidienne et l’utilisation d’un chélateur du calcium au cours d’une hospitalisation de deux semaines [7].

**Figure 47:** Caisson hyperbare Comex 2500 / Cornes *2500 hyperbaric chamber* (© S. Girardot) 
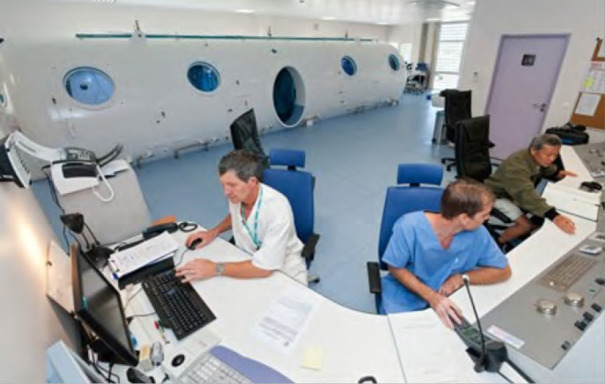

Au CHPf, l’hyperbarie médicale correspond à une activité transversale avec une collaboration active entre les services de chirurgie, de médecine, l’unité de « plaies et cicatrisation » et le caisson hyperbare. Une ou deux séances quotidiennes à la pression de 2,5 ATA sont réalisées cinq jours par semaine ainsi que la réfection de pansements. La majorité des séances concerne des patients hospitalisés et les protocoles effectués sont en adéquation avec les recommandations de l’HAS. Ce sont au total 1 400 séances-patient qui ont été réalisées en 2022 dans le cadre de ces indications médicales et 127 qui concernaient des accidents de plongée.

## Pharmacie

**Philippe Dupire**

La Pf étant compétente en matière de santé du fait de son statut d’autonomie, l’Agence nationale de sécurité du médicament (ANSM), l’Autorité de sûreté nucléaire (ASN) et l’Agence française du sang (AFS) n’y sont pas compétentes. En conséquence, le Pays dispose d’une organisation pilotée par le ministère polynésien de la Santé au travers de l’ARASS chargée de l’organisation des soins, les vigilances, la réglementation sanitaire, les autorisations concernant les médicaments sans autorisation de mise sur le marché (AMM). Elle gère les autorisations administratives associées (lits, places, équipements lourds). À ce titre, cette entité passe des conventions de soutien technique avec les agences ou les autorités métropolitaines telles l’ASN ou l’ANSM disposant des expertises utiles. Les médicaments sans statut d’AMM c’est-à-dire avec Autorisation d’accès compassionnel (AAC) ou Autorisation d’accès précoce (AAP), relèvent des deux juridictions : de la France pour le laboratoire et de la Pf pour l’autorisation d’importation^6^. Comme pour le maillage médical, celui de la pharmacie est organisé en une partie privée (libérale et cliniques), hospitalière spécifique du CHPf disposant de son propre financement, et en un approvisionnement dépendant de la Direction de la santé pour son réseau de dispensaires (soins primaires) et de structures hospitalières^7^. La Pf applique les règles financières et comptables françaises et impose des achats en France hexagonale, les résumés des caractéristiques du produit étant en français et la sécurité sanitaire reposant sur les règles françaises (retrait de lots…). Quelques médicaments spécifiques sont importés mais ils sont sources de difficultés logistiques importantes : paiement en devises étrangères comme pour les immunoglobulines anti-poisson-pierre venant d’Australie ou empreinte carbone de dispositifs médicaux comme des échographes achetés à Singapour et « européanisés » en France pour un marquage CE avant envoi en Pf. Pour les stupéfiants, la Convention de Vienne qui définit des quotas par pays, impose une autorisation d’importation pour la Pf et une autorisation d’exportation pour le laboratoire national. Ces mesures imposent une vigilance importante car il est impossible de les avoir en urgence (au moins cinq semaines par avion) : un jour de consommation au CHPf représentant un mois de consommation de l’ensemble des autres structures de santé, il est ainsi impossible de se dépanner autre part en cas de rupture de stock. Ces contraintes peuvent également se décliner pour les psychotropes, les précurseurs de substances illicites (éphédrine…), les médicaments radioactifs… Par ailleurs, pour le moment, toute substance issue du cannabis est interdite.

L’insularité et l’éloignement géographique imposent de plus des contraintes de transport et de stockage. Le transport est aérien ou maritime, cette voie imposant une anticipation des commandes puisqu’il faut compter en moyenne trois mois de délai entre la commande et l’arrivée, à condition que la mer soit clémente, que des containers ne soient pas perdus en mer ou qu’un retrait de lot ne se fasse pas pendant le trajet, sans compter la problématique des ruptures mondiales. Certains produits de santé ne sont également pas transportables par avion du fait d’un risque aérien ou de contraintes de volume ou poids (les avions cargo ne peuvent pas atterrir en Pf soit par une longueur de piste insuffisante soit par manque de rentabilité pour les affréteurs). Ainsi, le transport des produits de dialyse ou de certains désinfectants est exclusivement maritime et donc à risque de rupture. Un minimum de stock d’environ trois mois est donc nécessaire et imposant (5 000 m^2^ de surface logistique) quand seulement six jours sont suffisants pour une pharmacie à usage intérieur (PUI) comparable en métropole. Le transport aérien est quant à lui utilisé pour les médicaments rares et/ou coûteux (traitement d’oxalurie primitive, maladie de Hunter, hémophilie A, facteur VII.), le transport en température maîtrisée ou les urgences vitales. Celles-ci restent cependant relatives car, en raison du décalage horaire, le vendredi matin en Pf, il est déjà vendredi soir en France métropolitaine et les transitaires et laboratoires pour l’export sont fermés. La gestion de traitements relevant de l’urgence vitale nécessite donc de développer un réseau particulier qui passe par des contacts avec des laboratoires, des PUI nationales (Assistance Publique -Hôpitaux de Paris), le réseau Europ Assistance… Le fret aérien est par ailleurs aléatoire, car il repose sur des vols commerciaux de passagers, qui restent prioritaires. Un débarquement des marchandises peut survenir à l’escale américaine en cas de problème technique. La stabilité des températures pendant le transport des médicaments doit être garantie, ce qui peut retarder la prise en charge des patients. Des ruptures du pont aérien sont également possibles, comme cela a été observé lors d’événements majeurs (11 septembre 2001, éruption volcanique en Islande en 2010, crise du Covid-19…). Enfin, une concurrence peut exister entre les médicaments et les denrées périssables à certaines périodes de l’année. Ainsi, les vols sont généralement fermés au fret les 24 et 30 décembre.

Le CHPf se doit également de garder une certaine autonomie dans la production de médicaments grâce aux compétences techniques et matérielles que lui seul possède en Pf quand le délai d’accès aux thérapeutiques est déraisonnable pour assumer l’arrivée d’un traitement au regard de l’urgence ou de la perte de chance. Ainsi, toutes les chimiothérapies anticancéreuses de Polynésie française sont réalisées à la PUI. Un transfert vers des sites distants, parfois très éloignés, est possible - comme vers Raiatea, située à 300 km de Tahiti - ce qui implique la gestion du transport aérien, de la sécurité bactériologique, du maintien de la température et du retour des déchets. La PUI assure également la préparation de la nutrition parentérale pour l’unité de réanimation néonatale. Elle est dotée d’une production autonome d’oxygène médical *in situ,* certifiée ISO 9001, avec une capacité passée de 400 L/min à 4 000 L/min durant la crise du Covid-19. Enfin, elle prend en charge les préparations aseptiques (notamment les collyres fortifiés) et l’ensemble des préparations hospitalières, en particulier pédiatriques, lorsque les spécialités disponibles ne sont pas adaptées. Cela inclut les cas où l’accès à un médicament sous AAP ou AAC est incompatible avec l’urgence^8^.

**Figure 48:** Hotte blindée dédiée à la synthèse du ^68^Ga-PSMA11, un émetteur de positons utilisé en imagerie TEP pour le suivi des récidives de cancer de la prostate / *Shielded fume hood dedicated to the synthesis of ^68^Ga-PSMA11, a positron-emitting radiotracer used in PET imaging for monitoring prostate cancer recurrence* (© P. Dupire) 
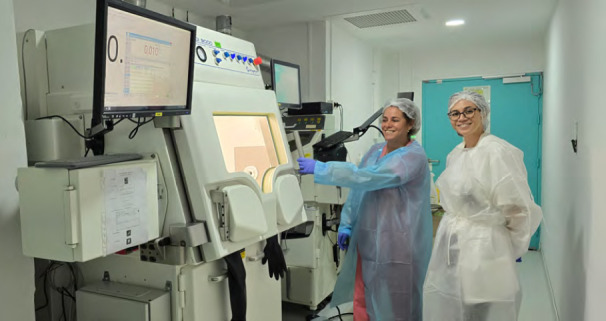

La PUI gère la médecine nucléaire (Fig. 48) et la radiopharmacie qui nécessitent de transporter des sources radioactives par avion sur des long-courriers et d’anticiper l’activité résiduelle à l’arrivée (calcul des activités pour l’^131^I et le ^99m^Tc). Le transport de certains radio-isotopes est cependant non réalisable en raison d’une demi-vie trop courte (^9^F = 109 minutes). Cette situation nécessite l’installation prochaine d’un cyclotron et d’assurer la chaîne complète de fabrication : synthèse du ^18^FDG, contrôle de pureté radiobiologique et radiopharmaceutique par chromatographie en phase liquide à haute performance, dosage des endotoxines et contrôle de stérilité avant injection. En France, le ^68^Ga-PSMA-11, dans l’indication de récidive de cancer de prostate, impose pour le moment une demande d’AAC pour chaque patient après avoir fait une ^18^F-choline; il a également l’autorisation dans le bilan préthérapeutique de l’AAC de cohorte du ^177^Lu-PSMA-617 mais il faut également une imagerie avec la ^18^F-choline. En l’absence de possibilité d’accès au ^18^F-choline en Pf, une transgression au contexte réglementaire devra être réalisée au bénéfice du patient. L’activité de stérilisation (ISO 9001) subit les mêmes contraintes avec une logistique, une maintenance et une diversité sans comparaison avec une stérilisation en France hexagonale (avec la nécessité d’une maintenance de haut niveau, notamment sur les stérilisateurs basse température de type plasma d’H_2_O_2_).

En dehors du CHPf et pour le reste de la Pf, il existe un grossiste répartiteur public, la pharmacie d’approvisionnement, qui alimente en médicaments et dispositifs médicaux une soixantaine de points de santé (médecine ambulatoire) et les trois mini-structures hospitalières.

## Principales plantes toxiques de Polynésie

**Jean-François Butaud**

L’absence de statistiques portant sur les intoxications par les plantes ne nous permet que de discuter des plantes reconnues comme pouvant être toxiques par différents botanistes, ethnobotanistes, chimistes, biologistes ou médecins, l’ensemble de ces spécialistes tenant en grande partie leurs savoirs des connaissances des tradipraticiens et des dires des habitants et utilisateurs des plantes.

Les plantes toxiques sont notamment présentes dans plusieurs familles botaniques bien connues comme les Apocynacées, caractérisées par leur abondant latex blanc ou les Euphorbiacées. Elles regroupent également les plantes ichtyotoxiques à saponines^17^ ou à roténone^18^, cette dernière étant particulièrement active sur les animaux à sang froid. Enfin, plusieurs plantes alimentaires nécessitent une préparation toute particulière (lavage, cuisson…) afin d’éviter une intoxication lors de leur consommation.

En médecine traditionnelle, peu de plantes ont été indiquées comme potentiellement toxiques, la plus connue, car impliquée dans des décès et qui a cristallisé les critiques étant la fougère *metuapua’a (Microsorum grossum),* très employée dans la pharmacopée polynésienne dans le traitement des contusions, entorses, fractures, leucorrhées, coliques hépatiques, brûlures d’estomac, vomissements… Relativement à la médecine traditionnelle polynésienne, plusieurs études ethnobotaniques récentes permettent de l’appréhender et le lecteur pourra s’y référer en tant que de besoin [54,55,106,161,196]. Plusieurs initiatives menées par le Pays, le CHPf, certaines communes et des associations (Haururu, Honoea.) visent depuis quelques années à l’émergence d’une médecine intégrative polynésienne englobant la médecine traditionnelle.

Nous présentons ci-dessous, par ordre alphabétique des familles, les principales plantes toxiques ou potentiellement toxiques rencontrées en Polynésie française ayant notamment été rapportées par plusieurs sources bibliographiques [57,119,169,192,232].

### Anacardiacées

L’anacardier, *Anacardium occidentale* L., originaire d’Amérique du sud, autrement appelé noix de cajou, pomme cajou, acajou ou *‘anatārita* à Tahiti, est un arbre fruitier introduit en 1875 dont on mange la pomme (pédoncule charnu du fruit) ainsi que l’amande rôtie ou cuite contenue dans la noix. Par ailleurs, le péricarpe constituant l’enveloppe de l’amande contient plusieurs substances caustiques et allergisantes qui provoquent de fortes irritations de la peau et des muqueuses. Dans cette famille, il faut également citer l’arbre indigène *Rhus taitensis* Guill., nommé *apape* ou *‘avai* à Tahiti, qui possède dans son écorce un suc irritant et provoquant des éruptions cutanées. Ainsi, le travail de son bois conduit à des irritations de la peau causées par la sciure volatile comme l’indiquait l’ébéniste Daniel Duprat (communication personnelle).

### Apocynacées

Une des plantes les plus toxiques de Pf est *Cerbera manghas* L. (Fig. 49), *reva* et *hotureva* dans la Société et aux Australes, *‘eva* aux Marquises, également appelée tanghin ou faux-manguier en raison de la ressemblance de son fruit avec une mangue. Il s’agit d’un arbre indigène se développant essentiellement sur le littoral, mais qu’il est possible de trouver à l’intérieur des terres où il a été anciennement planté pour ses usages traditionnels. Plusieurs parties de la plante pouvaient être employées dans la pharmacopée (notamment en tant que purgatif^19^) tandis que le fruit servait d’ichtyotoxique, mais également pour les suicides et les empoisonnements criminels. Cette espèce est mondialement connue pour sa toxicité causée par la cerbérine, un hétéroside cardiotonique. Plusieurs arbustes ornementaux introduits appartenant à la même famille sont également réputés pour leur toxicité causée par de tels hétérosides cardiotoniques : *Calotropis gigantea* (L.) W.T. Aiton d’Asie du Sud-Est, *Cascabela thevetia* (L.) Lippold (syn. *Thevetia peruviana* (Pers.) K. Schum.), le laurier-rose à fleurs jaunes d’Amérique tropicale, ou encore *Nerium oleander* L., le laurier-rose ou *tarona* de la région méditerranéenne.

**Figure 49:** *Cerbera manghas* / Cerbera manghas (© JF Butaud) 
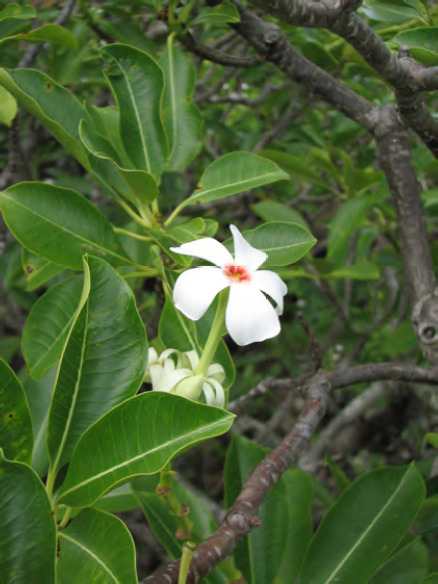

19. Les Polynésiens se purgent régulièrement afin de prévenir certaines maladies. Ces purges ont généralement comme base l’huile tirée de l’amande de la noix de coco. D’autres plantes peuvent entrer dans la composition des purges rendues nécessaires par le traitement d’une maladie, afin d’expulser et la maladie et le remède à base de plantes.

20. Corme : organe de réserve souterrain ayant l’aspect d’un bulbe mais formé d’une tige renflée entourée décailles.

### Aracées

Plusieurs Aracées alimentaires, d’introduction polynésienne en provenance de l’ouest du Pacifique, cultivées ou subspontanées sont connues pour leurs feuilles irritantes ou corrosives et leurs tubercules toxiques à l’état cru : *Alocasia macrorrhizos* (L.) G.Don ou *‘ape, kape* ou oreille d’éléphant, *Colocasia esculenta* (L.) Schott ou *taro, ta‘o,* ainsi que *Amorphophallus paeoniifolius* (Dennst.) Nicolson ou *teve.* Les cormes^20^ contiennent en effet des cristaux d’oxalate de calcium qui sont décomposés suite à la cuisson. Pour certaines espèces, la cuisson longue pouvait être précédée d’un rouissage. La substance toxique renfermée dans les feuilles de taro est éliminée par extraction des principales nervures suivie d’une cuisson soigneuse. Dans la même famille, les diverses espèces ornementales appartenant au genre américain *Dieffenbachia,* introduites depuis le milieu du 20^e^ siècle, sont toutes toxiques et il faut donc se méfier de la sève contenue dans leurs feuilles ou dans leurs stipes.

### Campanulacées

L’étoile de Bethléem, herbacée ornementale introduite durant la première moitié du 20^e^ siècle, originaire de Jamaïque, *Hippobroma longiflora* (L.) G.Don, est bien connue des pâturages où elle n’est pas consommée par les herbivores en raison de sa toxicité. En effet, elle contient une sève très irritante, notamment pour les yeux, mais également des alcaloïdes cardioactifs pouvant être mortels par ingestion.

### Dioscoréacées

Parmi les ignames comestibles, *Dioscorea bulbifera* L., l’igname bulbifère ou *hoi* d’introduction polynésienne, originaire de l’ouest du Pacifique, et présente à l’état sauvage dans les îles hautes, est parfois considérée comme une plante toxique. En effet, ses tubercules souterrains et aériens (pomme-en-l’air) sont toxiques à l’état cru en raison de la présence d’un alcaloïde qui n’est détruit qu’après un lavage (substance soluble dans l’eau) suivi par une cuisson prolongée. *Tacca leontopetaloides* (L.) Kuntze, *pia* ou *arrow-root* de Tahiti, également d’introduction polynésienne et originaire de l’ouest du Pacifique, possède un tubercule alimentaire autrefois largement cultivé pour sa fécule employée dans la confection des desserts nommés *po‘e* ou *poke* en Polynésie. Ces tubercules toxiques à l’état cru, renfermant une substance âcre, deviennent comestibles après lavage et cuisson. Ainsi, le corme est râpé, puis trempé plusieurs jours de suite en changeant régulièrement l’eau avant que la pâte obtenue ne soit prélevée après décantation de la dernière eau.

### Euphorbiacées

Le bancoulier *Aleurites moluccanus* (L.) Willd. nommé *ti‘a‘iri* et anciennement *tutui* dans la Société et aux Australes, *‘ama* aux Marquises ou encore *rama* aux Gambier, est un arbre originaire de l’ouest du Pacifique, d’introduction polynésienne et doté de multiples usages. Son écorce est ainsi employée en médecine traditionnelle pour traiter les plaies infectées, les angines et les calculs rénaux tandis que ses amandes sont employées pour la fabrication de chandelles ou pour la confection de l’encre des tatouages. Il produit par ailleurs des amandes aux propriétés purgatives lorsque trois ou quatre sont ingérées successivement. Selon certains auteurs, la torréfaction atténue cette propriété tandis que Nadeaud (1864) notait qu’il pouvait consommer sans modération ces amandes une fois l’embryon ôté. Parmi les autres Euphorbiacées, il est possible de citer le ricin, *Ricinus communis* L. ou *titiuna*, qui est un arbuste des bords de route, dépotoirs et terrains vagues dont la toxicité des graines liée à la ricine est bien connue. Originaire d’Afrique de l’Est, le ricin a été introduit avant 1823 à Tahiti. Plusieurs espèces ornementales introduites appartenant aux genres *Euphorbia* et *Jatropha* produisent des latex et fruits toxiques. Enfin, il faut signaler que les cultivars polynésiens de manioc, *Manihot esculenta* Crantz, sont dits « doux » et ne contiennent pas d’acide cyanhydrique ou uniquement dans l’épiderme éliminé avant consommation. Originaire d’Amérique du Sud, le manioc a été introduit en 1850 à Tahiti.

### Fabacées

Les graines rouges et noires de la liane d’introduction polynésienne *Abrus precatorius* L. subsp. *precatorius,* appelée *pitipiti‘o* dans la Société ou *poniu* aux Marquises, sont très souvent employées dans la confection de colliers (Fig. 50). Elles contiennent une toxalbumine très toxique, l’abrine, qui peut provoquer des empoisonnements mortels si les graines, très dures par ailleurs, sont brisées puis ingérées. Cette famille comprend également deux espèces très connues comme poisons de pêche/ichtyotoxiques en raison de leur teneur en roténoïdes : la liane malésienne introduite *Derris montana* (Benth.) Prain (syn. *D. malaccensis* Benth.) appelée *hora pāpua,* dont les racines sont riches en roténone, et **l’arbrisseau d’introduction polynésienne** ***Tephrosia purpurea*** **(L.) Pers. subsp.** ***purpurea*** **appelé** ***hora*** **ou** ***kohuhu*** **dont la plupart des organes contiennent de la téphrosine. Même si ces principes actifs ciblent les animaux à sang froid, ils semblent pouvoir être toxiques pour les humains dans certaines conditions.**

**Figure 50:** *Abrus precatorius* / Abrus precatorius (© JF Butaud) 
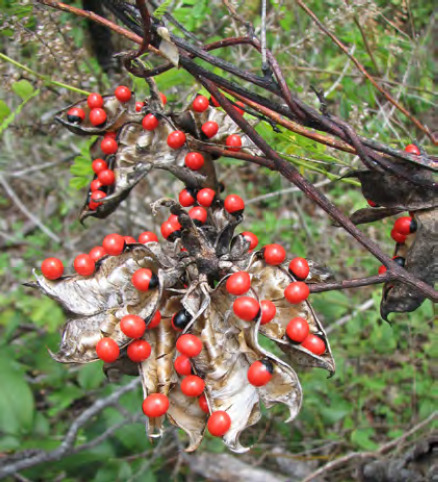

### Lécythidacées

Les amandes du grand arbre indigène *Barringtonia asiatica* (L.) Kurz, appelé *hotu* ou *hutu* dans l’ensemble de la Polynésie et parfois appelé « Bonnet d’évêque » en français, en raison de la forme du fruit, étaient communément employées en tant qu’ichtyotoxiques par le passé. Ces amandes sont considérées comme vénéneuses par les tradipraticiens, cette toxicité provenant probablement de sa richesse en saponines.

### Oxalidacées

Récemment, la consommation de caramboles (Fig. 51) ou *raparapa,* issues de l’arbre introduit et cultivé originaire d’Indonésie, *Averrhoa carambola* L., a été démontrée comme néfaste pour les insuffisants rénaux et dialysés, car pouvant induire des atteintes rénales et neurologiques, en raison de la toxine caramboxine contenue dans son fruit.

### Polypodiacées

La fougère indigène *Microsorum grossum* (Langsd. & Fisch.) S.B.Andrews (syn. *Phymatosorus grossus* (Langsd. & Fisch.) Brownlie) (Fig. 52), connue sous les noms polynésiens *metuapua‘a* et *atuapua‘a* dans la Société, *papamoko* et *papamo‘o* aux Marquises, *kikipa* aux Tuamotu, *mo‘omo‘o* aux Australes ou encore *moku papa* aux Gambier, fait partie des ingrédients les plus utilisés en pharmacopée polynésienne. Elle est employée notamment pour traiter les «*fati »»* (chocs, contusions, fractures…), mais également comme purgatif et vermifuge chez les enfants, et a été impliquée dans des intoxications chez des nouveau-nés et de jeunes enfants depuis les années 1960 et encore jusqu’à récemment. Ces intoxications, parfois mortelles, pourraient être liées, selon Pétard (2019), à des erreurs de cueillette (confusion avec d’autres fougères), à de trop fortes doses (quantité, fronde ou rhizome, saison, écologie.) ou encore à des préparations défectueuses (autres ingrédients, eau, ustensiles, conservation.). Depuis, des études chimiques des différentes espèces de *Microsorum* polynésiennes ont été conduites à Tahiti et ont démontré la présence de phyto-ecdystéroïdes dans les frondes et rhizomes, molécules connues pour avoir divers effets pharmacologiques sur les humains et autres mammifères (Ho *et al.,* 2007) : anabolisant, hypoglycémiant, hypocholestérolémiant, tonique, hépatoprotecteur, antidépresseur et purgatif.

**Figure 51:** *Averrhoa carambola* / Averrhoa carambola (© JF Butaud) 
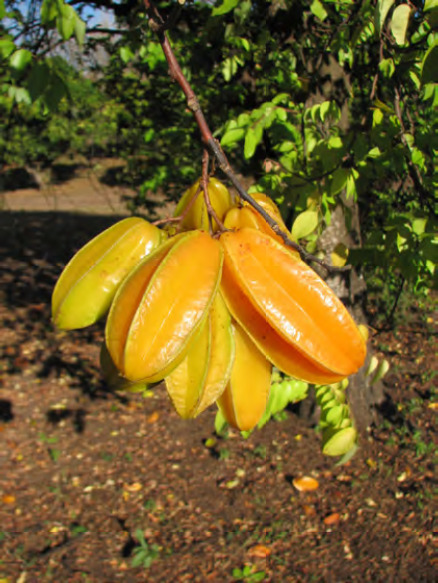

**Figure 52:** *Microsorum grossum* / Microsorum grossum (© JF Butaud) 
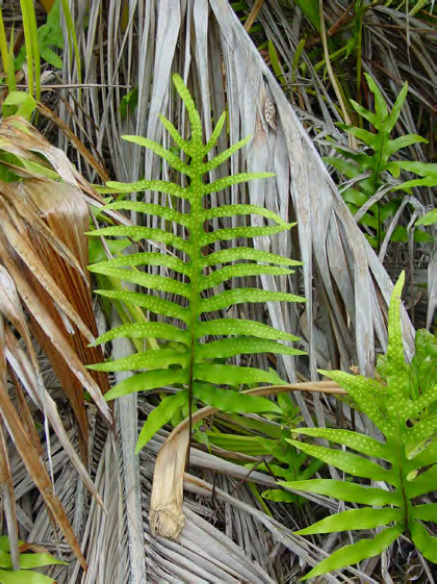

### Solanacées

Trois Solanacées américaines particulièrement toxiques ont été introduites en Polynésie, certaines dès le milieu du 19^e^ siècle, et sont connues sous les noms de datura, trompette du diable, *tatura, pūreo* et *pū*

*tautau* dans la Société ou encore *pua tīoi fenua* aux Marquises : l’arbrisseau cultivé et parfois naturalisé *Datura metel* L. ainsi que deux *Brugmansia* arbustifs ornementaux, cultivés et parfois subspontanés, *Brugmansia suaveolens* (Humb. & Bonpl. ex Willd.) Sweet et *Brugmansia x candida* Pers. Bien que toutes les parties de ces plantes soient toxiques, elles sont encore assez régulièrement cultivées pour leurs grandes fleurs ornementales et, malgré cela, elles sont employées par les amateurs de psychotropes pour leurs propriétés hallucinogènes, leur consommation pouvant entraîner des séquelles neurologiques permanentes, voire la mort.

### Thyméléacées

Le petit arbre endémique de Polynésie orientale *Wikstroemia coriacea* Seem. (Fig. 53) nommé *‘o‘ovao* ou *‘avao* dans la Société et *ka‘apihi, katea* ou *hihea* aux Marquises est employé en pharmacopée traditionnelle, notamment en tant que purgatif dans les cas d’empoisonnement, mais également pour la confection de *tapa* (étoffe végétale) avec son écorce interne. Sur ce dernier point, il est réputé pour irriter la peau et les yeux lors du battage de son écorce. En pharmacopée, ses feuilles, ses racines et son écorce sont réputées vomitives, purgatives et vésicantes; Pétard (2019) indiquait dans son ouvrage que « *la médication entraînait un état de prostration et d’adynamie qui pouvait persister plusieurs heures* ».

### Urticacées

**Figure 53:** *Wikstroemia coriacea* / Wikstroemia coriacea (© JF Butaud) 
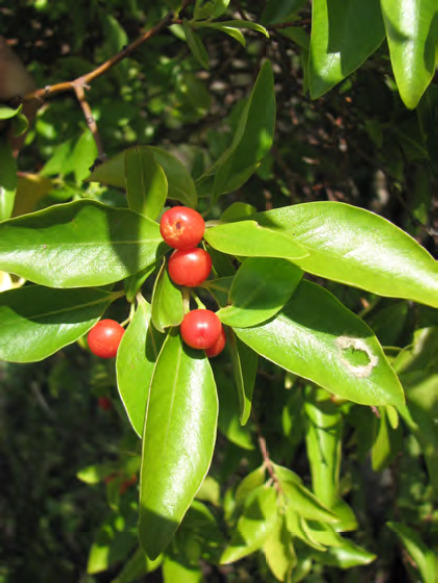

Quelques orties européennes appartenant au genre *Urtica* ont été récemment introduites à des fins agricoles (engrais et répulsif contre les ravageurs), mais leur usage demeure confidentiel. Il est néanmoins possible de citer une ortie arborescente indigène rarissime, car non retrouvée depuis le 19^e^ siècle, *Dendrocnide harveyi* (Seem.) Chew, appelée *haratô* en tahitien (cuisant, qui démange) et bien connue pour ses propriétés urticantes dans tout le Pacifique. Si quelqu’un en était victime, il serait pertinent que l’information puisse remonter jusqu’aux botanistes qui sont à la recherche de cette plante « disparue » !

### Éléments culturels dans le contexte de soins polynésien

**Christel Thomas**

En Polynésie française, le contexte de soins peut être comparé à une microsociété où chaque intervenant est face à l’altérité culturelle, terrain de rencontre contraint pour certains, choisi pour d’autres où interagissent les différences. À l’inverse de les isoler et de les affronter, il est indispensable d’identifier les facteurs humains et culturels qui interfèrent dans les soins afin de répondre au plus près aux besoins de santé des patients.

Le français est la seule langue officielle en Pf comme dans tout le territoire de la République, il est compris et parlé par une grande majorité de la population. Sept langues vernaculaires polynésiennes (tahitien, austral, ra‘ivavae, rapa, pa‘umotu, mangarévien et marquisien) et une langue d’origine chinoise (hakka) sont utilisées couramment dans les actes du quotidien et surtout lorsqu’il s’agit d’évoquer le registre des émotions et de l’intime. Le tahitien (reo *Tahiti)* a le double statut de langue vernaculaire^10^ et de langue véhiculaire^11^ [233]. L’utilisation d’un remplacement lexical en français dialectal sous l’influence de la langue substrat en situation de contact est de nos jours plutôt commun au locuteur. Il n’est donc pas rare par exemple d’entendre « se baigner » pour « se doucher » ou encore « j’ai bien bu mes graines » pour « j’ai bien pris mes médicaments ». Les difficultés de compréhension, mais aussi les malentendus ou quiproquos, ne sont pas rares dans la relation soignant/soigné.

La médecine contemporaine est exercée par des médecins (*taote*^12^) majoritairement métropolitains. Elle utilise un vocabulaire et un cadre de réflexion propres à sa discipline, ainsi qu’un ensemble de moyens (thérapeutiques, relationnels, juridiques) souvent méconnus par le patient polynésien. Ce dernier adoptera plutôt une attitude de retrait, mettant sous silence son incompréhension, et laissant place à une forme de réserve voire de méfiance pouvant aller jusqu’à la critique ou l’invalidation. À l’inverse, on peut retrouver des attitudes plus « passives » où le patient se contentera de dire « c’est toi qui sais *taote* » et s’installera dans une alliance thérapeutique de surface [170,230].

Très peu d’éléments de la conception polynésienne des maladies sont parvenus jusqu’à nous. L’on sait notamment que dans la médecine traditionnelle polynésienne, les pathologies étaient classifiées selon l’origine attribuée du trouble. Selon Orsmond Walker, Tiurai, qui était un *tahu’a* (guérisseur) renommé de Tahiti né au début du 19^e^ siècle, guérissait les maladies du corps *(ma‘i tino)* et les maladies de la pensée *(ma‘i mana‘o).* Tiurai admettait cependant comme une maladie réelle de la pensée la maladie de l’esprit *(ma‘i varua)* et la maladie de l’âme *(ma‘i vaite).* Cette dernière n’était jamais la maladie de l’âme du vivant qui en souffrait, mais de celle d’un ancêtre ou d’un parent défunt, tandis que dans la maladie de l’esprit c’était l’esprit du vivant même qui souffrait [231]. Les études de la médecine traditionnelle récentes ne se sont intéressées qu’aux maladies du corps, le plus souvent à soigner avec des plantes (parfois des animaux ou des roches), les autres sujets n’ont pas été abordés. Il conviendrait par ailleurs de mener des études spécifiques dans la Société et les autres archipels pour éventuellement recueillir ce qui reste de ces savoirs. Il n’existe rien d’autre dans la bibliographie sur les différents types de maladies que ce qu’a mentionné Tiurai et que Simone Grand a repris [109,110].

Le cadre de référence des Polynésiens, évolutif dans le parcours de vie et de soins du patient, peut s’avérer être une ressource ou un frein dans sa prise en charge. Il intègre la structure familiale et l’éloignement géographique :

- dans la Polynésie ancestrale, le terme *‘utuāfare* était utilisé pour désigner une communauté familiale de cinq à vingt personnes se reconnaissant par les liens familiaux, de mariage ou d’adoption [180]. Aujourd’hui, dans ce groupe résidentiel de base, la personne qui a l’autorité juridique sur l’enfant (bien souvent le géniteur), n’est pas forcément la personne qui s’occupe de lui. En 2012, l’ISPF estimait que 39 % des enfants vivaient au sein d’une famille élargie et cette réalité est encore présente aujourd’hui. Les études récentes portant sur les dynamiques familiales et identitaires des familles polynésiennes (notamment les choix familiaux, les choix individuels, la nature des couples -polynésiens, mixtes, métropolitains -, les discours, la mobilité géographique) notent que la tendance actuelle de l’organisation familiale est caractérisée par un glissement de l’organisation en familles élargies vers des familles nucléaires, tant par la reproduction des modèles familiaux occidentaux que par l’urbanisation [18,212]. Si, traditionnellement, cette particularité polynésienne impliquait le renforcement des liens familiaux, de nos jours, elle peut apparaître comme problématique pour le patient. Sa difficulté à se situer au sein de la structure familiale génère une instabilité, délétère dans le processus de soins;
- l’éloignement géographique est un paramètre inévitable à prendre en compte au sein du territoire polynésien vaste comme l’Europe. Pour exemple, entre Tahiti, lieu de soins, et l’archipel des Marquises, lieu de vie de certains patients, la distance est d’environ 1 500 km. Si une indication d’hospitalisation est posée, il sera nécessaire de programmer une évacuation sanitaire par voie aérienne ou maritime. Outre l’aspect logistique, cela pose la question du lien et de son maintien : les relations fiables et sûres sont mises à distance, créant de l’insécurité psychique, l’attachement au groupe repérant est rompu amenant à un sentiment d’anxiété jusqu’à des perturbations dans les autres relations sociales. L’hospitalisation peut être perçue comme menaçante ou excluante et met à mal la capacité décisionnaire souvent portée par le groupe familial ou son représentant. Dans ce contexte de séparation, le renoncement aux soins peut être favorisé.

Face à la maladie et la vulnérabilité qui en découle, la religion et le recours aux soins traditionnels sont des pratiques courantes associées à la réponse médicale occidentale. Elle se manifeste très régulièrement par des lectures de Bible ou des temps de prières lors de visites de l’entourage. La religion chrétienne demeure aujourd’hui très présente dans la société polynésienne et occupe une fonction sociale importante. Deux confessions sont prédominantes, l’Église protestante *mā’ohi* et le catholicisme. D’autres Églises se sont développées mais restent minoritaires : Église de Jésus-Christ des Saints des Derniers Jours,

Témoins de Jéhova, Église Sanito (Église de la Communauté du Christ), Église adventiste du septième jour. La foi et la prière s’inscrivent dans le temps d’hospitalisation du patient et dans le processus de guérison.

La médecine traditionnelle ne restreint pas son champ d’action à l’individu et intègre l’entourage familial dans l’intérêt d’un bien-être commun. Elle ne doit pas être mise en concurrence ou en opposition avec la médecine moderne, mais plutôt associée pour les rendre complémentaires. De nos jours, les soins traditionnels pratiqués sont à base de plantes médicinales appelées *rā‘au mā’ohi* ou de soins corporels à type de massages *(taurumi)* ou de bains *(hopu).* Des solutions buvables *(inu)* peuvent être également consommées. L’accent est mis sur la nécessité de considérer ces approches, contemporaines et traditionnelles, comme complémentaires plutôt que divergentes.

Au regard des différents facteurs propres à la population polynésienne que sont la langue, le rapport au soignant, la représentation de la maladie et le cadre de référence, les pathologies rencontrées sur le territoire et le modèle de soins métropolitain proposé doivent s’inscrire dans un contexte sanitaire d’interculturalité afin d’optimiser leurs prises en charge et le processus de guérison.

## Conseils aux voyageurs

**Erwan Oehler**

La Polynésie française est une destination relativement sûre et peu risquée à condition de respecter certaines règles.

Les vaccins doivent être mis à jour selon le calendrier vaccinal en vigueur. La vaccination contre l’hépatite B est fortement recommandée en raison de la prévalence élevée de celle-ci. La Pf est exempte de fièvre jaune mais ce vaccin est obligatoire pour les voyageurs en provenance de pays endémiques.

Le voyage de Paris vers Tahiti est long : environ 10h30 de Paris vers Los Angeles (LAX) ou San Francisco (SFO) puis environ 9h entre LAX/ SFO et Papeete après 2 à 3 heures d’escale aux États-Unis. Les vols long-courriers sont un facteur de risque de thrombose veineuse et peuvent nécessiter le port d’une contention élastique et/ou d’une thromboprophylaxie en fonction des facteurs de risque individuels.

Une bonne hydratation et la mobilisation des membres pendant le vol permettent de limiter ce risque. Un décalage de 12h (11h en hiver) existe entre l’Hexagone et la Pf. Il faut donc anticiper la gestion des médicaments au long cours du fait de la longueur du vol.

La Pf a un fort ensoleillement avec un indice UV élevé qui nécessite l’utilisation d’une protection solaire. Le climat, chaud et humide, est également propice aux coups de chaleur et requiert une hydratation suffisante et une acclimatation progressive à l’effort des nouveaux arrivants tout en évitant les efforts violents et prolongés aux heures les plus chaudes de la journée.

Une protection antivectorielle est souhaitable et passe par l’utilisation de répulsifs à dosage suffisant et le port de vêtements longs. Les moustiques du genre *Aedes* sont de potentiels vecteurs d’arboviroses (Ae. *aegypti)* ou de filaires (Ae. *polynesiensis)* et piquent essentiellement le jour et au coucher du soleil. Les moustiques du genre *Culex* notamment *Cx. quinquefasciatus* ne transmettent aucun agent pathogène et piquent seulement la nuit. Les *nono* (simulies et cératopogonidés) sont des moucherons hématophages qui ne sont nuisibles que par le nombre de piqures et l’intense prurit dont ils sont responsables. Outre le risque viral lié aux moustiques, les piqures peuvent être responsables d’infections cutanées liées au prurit.

L’eau de distribution dans la plupart des communes urbaines de Tahiti et Moorea est potable de même qu’à Bora Bora ou à Uturoa (Raiatea). Il convient de se renseigner sur la potabilité de l’eau des autres communes.

Les randonnées en montagne sont mal ou peu balisées avec des risques en cas de fortes pluies : éboulements, crues de rivières… Il est nécessaire de se renseigner sur les conditions météorologiques et de prévenir un tiers avant le départ. Le risque de noyade est à considérer en cas de baignade ou de plongée. Pour limiter le risque lié à la faune marine, il convient de ne pas toucher à l’environnement et aux organismes marins. La consommation de poisson du lagon est à risque de ciguatéra qu’il faudra savoir évoquer en cas d’apparition de signes digestifs, cardiovasculaires ou neurologiques.

En raison de l’augmentation de l’incidence de l’infection VIH et des IST, les rapports sexuels protégés sont recommandés. Le traitement post-exposition est disponible aux urgences du CHPf.

En cas de fièvre chez une personne revenant de Pf, il convient d’éliminer une infection cosmopolite (infection respiratoire, urinaire, cutanée, pathologie chirurgicale) ainsi qu’une arbovirose ou une leptospirose si la date de retour est inférieure à dix jours.

## Conclusion

La Polynésie, territoire français aux antipodes de l’Hexagone, est autant un régal pour les yeux grâce aux magnifiques paysages et plages paradisiaques qu’une source de curiosité médicale par la richesse et la diversité des pathologies rencontrées. Même si son caractère insulaire l’a quelque peu préservée de nombre de pathologies tropicales et animaux dangereux, il n’en reste pas moins que les pathologies infectieuses y sont fréquentes. De plus, l’importante prévalence des maladies métaboliques est associée à de nombreuses complications que l’on rencontre habituellement dans l’Hexagone chez des patients plus âgés. Les spécificités culturelles, omniprésentes, font que le praticien peut se heurter à des difficultés de prise en charge ou de compréhension de ses patients. Ce panorama des pathologies infectieuses et non infectieuses de Polynésie française a pour vocation de dresser un tableau du système de santé polynésien et des pathologies que l’on peut y rencontrer afin de renseigner le lecteur ou les soignants exerçant ou souhaitant exercer en Pf ainsi que les soignants de l’Hexagone prenant en charge un patient en provenance du Pacifique. Ses ressources de niveau européen permettent une prise en charge diagnostique et thérapeutique inexistantes dans les autres nations insulaires du Pacifique sud (îles Cook, île de Pâques, Samoa, Tonga, Kiribati, Palau…). Les nombreux auteurs de ce document, du Centre hospitalier de Polynésie française, des différentes structures de la Direction de la santé ou d’ailleurs espèrent que celui-ci intéressera le lecteur grâce à la diversité des pathologies exposées et donnera envie aux cliniciens de l’Hexagone de venir visiter ou exercer en Polynésie française.

## Remerciements

Nous remercions chaleureusement Daniel Dalet de d-maps.com pour la réalisation des cartes géographiques de la Polynésie française illustrant les Figures 1 et 2.

Nous remercions également celles et ceux qui ont, de près ou de loin, contribué à la réalisation de ce panorama, par leurs conseils, publications et données chiffrées concernant les pathologies rencontrées en Polynésie française.

## Financement

Ce travail n’a bénéficié d’aucun financement.

## Conflits d’intérêts

Aucun lien d’intérêts lié à ce travail n’a été déclaré.
